# Visualizing metabolic network dynamics through time-series metabolomic data

**DOI:** 10.1186/s12859-020-3415-z

**Published:** 2020-07-07

**Authors:** Lea F. Buchweitz, James T. Yurkovich, Christoph Blessing, Veronika Kohler, Fabian Schwarzkopf, Zachary A. King, Laurence Yang, Freyr Jóhannsson, Ólafur E. Sigurjónsson, Óttar Rolfsson, Julian Heinrich, Andreas Dräger

**Affiliations:** 1 Computational Systems Biology of Infection and Antimicrobial-Resistant Pathogens, Institute for Biomedical Informatics (IBMI), Sand 14, Tübingen, 72076 Germany; 2 Institute for Systems Biology, 401 Terry Ave. N., Seattle, 98109 WA United States; 3 Department of Computer Science, University of Tübingen, Sand 14, Tübingen, 72076 Germany; 4 yWorks GmbH, Vor dem Kreuzberg 28, Tübingen, 72070 Germany; 5 Systems Biology Research Group, Department of Bioengineering, University of California, San Diego, 9500 Gilman Drive, La Jolla, CA 92093-0412 United States; 6 Novo Nordisk Foundation Center for Biosustainability, Technical University of Denmark, Building 220, Kemitorvet, Kgs.Lyngby, 2800 Denmark; 7 Department of Chemical Engineering, Queen’s University, Kingston, ON K7L 3N6 Canada; 8 Center for Systems Biology, University of Iceland, Sturlugata 8, Reykjavík, 101 Iceland; 9 The Blood Bank, Landspítali-University Hospital, Reykjavík, 101 Iceland; 10 School of Science and Engineering, Reykjavík University, Menntavegi 1, Reykjavík, 101 Iceland; 11 German Center for Infection Research (DZIF), partner site Tübingen, Tübingen, 72076 Germany

**Keywords:** Data visualization, Metabolism, Metabolomics, Platelet, Red blood cell

## Abstract

**Background:**

New technologies have given rise to an abundance of -omics data, particularly metabolomic data. The scale of these data introduces new challenges for the interpretation and extraction of knowledge, requiring the development of innovative computational visualization methodologies. Here, we present GEM-Vis, an original method for the visualization of time-course metabolomic data within the context of metabolic network maps. We demonstrate the utility of the GEM-Vis method by examining previously published data for two cellular systems—the human platelet and erythrocyte under cold storage for use in transfusion medicine.

**Results:**

The results comprise two animated videos that allow for new insights into the metabolic state of both cell types. In the case study of the platelet metabolome during storage, the new visualization technique elucidates a nicotinamide accumulation that mirrors that of hypoxanthine and might, therefore, reflect similar pathway usage. This visual analysis provides a possible explanation for why the salvage reactions in purine metabolism exhibit lower activity during the first few days of the storage period. The second case study displays drastic changes in specific erythrocyte metabolite pools at different times during storage at different temperatures.

**Conclusions:**

The new visualization technique GEM-Vis introduced in this article constitutes a well-suitable approach for large-scale network exploration and advances hypothesis generation. This method can be applied to any system with data and a metabolic map to promote visualization and understand physiology at the network level. More broadly, we hope that our approach will provide the blueprints for new visualizations of other longitudinal -omics data types. The supplement includes a comprehensive user’s guide and links to a series of tutorial videos that explain how to prepare model and data files, and how to use the software SBMLsimulator in combination with further tools to create similar animations as highlighted in the case studies.

## Background

Over the last few decades, new technological developments have enabled the generation of vast amounts of “-omics” data [1]. These various -omic data types have helped bring new insights to a vast array of biological questions [2–4]. As more and more data are generated, however, researchers are faced with the enormous challenge of integrating, interpreting, and visualizing these data. The community has recognized these needs, focusing efforts on data visualization as a way to maximize the utility of biological data [5]. Data visualization is particularly crucial for a systems-level perspective of metabolic networks and pathways. Several excellent software tools were made available for drawing and exploring biological network graphs [6–10]. These tools provide impressive descriptions of the network and support for diverse analyses, including the mapping of omics data to networks. In this study, we present GEM-Vis as a new approach for the visualization of time-course metabolomic data in the context of large-scale metabolic network maps.

Metabolomic data provide snapshots of cellular biochemistry, presenting essential insights into a cell’s metabolic state [11, 12]. Visualization tools often allow users to overlay pathway maps with static data sets [6]. Recently, time-course metabolomic data sets that detail cellular changes over time are becoming more prevalent [13–16], leading to the need for dynamic visualizations that can capture the aspect of time [17]—an essential aspect of understanding complex processes such as changes in metabolic activity, concentration, or availability. Many visualization tools [18–21], however, do not yet provide support for the representation of dynamic content. Those visualization tools whose features do include time series visualization [5, 17, 20, 22–24] only provide static depictions of the data. Some progress has been made to provide a stepwise temporal representation of metabolomic data [25], but a robust and smooth dynamic solution for mapping time series data to networks has yet to be presented.

One reason for the current lack of convincing visual analysis methods for dynamically changing data sets is that time-dependent data add additional layers of complexity to the already difficult problem of visual network exploration. First of all, genome-scale metabolic networks (GEMs) can have enormous sizes: Some published metabolic network maps comprise several thousand biochemical reactions [26, 27], of which human beholders can simultaneously only grasp a very small fraction [28].

With the development of new experimental technologies and the subsequent generation of -omics data sets, life scientists are faced with the challenge of extracting actionable knowledge. New visualization methods are a critical way that the community can make strides toward making the most of complex data. Here, we present a new method for the visualization of longitudinal metabolomic data in the context of the metabolic network. We provide two case studies that examine (1) a baseline characterization of a physiological process and (2) a set of experimental perturbations that allowed for a side-by-side comparison of different experimental conditions. The introduction of this new visualization method has two significant implications.

The method introduced in this article provides a dynamic visualization of cellular processes. Tools such as Cytoscape [29] provide visual analysis of networks and supports plugins like TiCoNE [18] and CyDataSeries [30] for the visualization of time-course data. However, tools such as these or VANTED [20] only offer static representations of dynamic data. To our knowledge, only KEGGanim [25] offers a dynamic visualization of time-course data. The method presented here builds on KEGGanim by offering a smooth interpolation between time points and offers the further advantage of customization concerning the display of both data and the network itself. The method presented outlines an original development for visualizing complex biological data based in a way that a cognition study has found to be useful and support perception [31].

With a steadily increasing number of carefully prepared metabolic network layouts being published, we here assume a map to be available for the system of interest. If this is not yet the case, a map can be easily drawn using software such as Escher [6]. This paper focuses on the problem of displaying dynamically changing quantitative data of network components. The aim is to answer the question: How to create expressive visual displays of dynamic metabolic networks? Needed are strategies to visually present the data in a way that beholders can best perceive and estimate quantities of network individual components and that at the same time enable them to conceptually narrow down parts of interest even within large networks.

In the next sections, we present the new method GEM-Vis (*Genome-Scale Metabolic model Visualization*) for the visualization and contextualization of longitudinal metabolomic data in metabolic networks. We developed three different graphical representations of metabolic concentration that allow for different interpretations of metabolomic data through a smooth animation. The method is implemented in the freely available software SBMLsimulator. The supplementary material of this article includes links to a series of four short tutorial videos that explain all aspects needed for creating a GEM-Vis: (1) where to obtain SBMLsimulator, how to run it, (2) where to obtain systems biology models and how to load them into the application, and how to create a simulated time-course data set from the model that can be mapped to an automatically generated pathway map, (3) where to obtain a manually drawn pathway map as well as published time-course data of the yeast *Saccharomyces cerevisiae* and how to prepare the data set for the import to SBMLsimulator and how to embed this layout information [32] in an SBML file [33], (4) how to load model and data into SBMLsimulator to create a GEM-Vis animation video including variation of several visual attribues and to save it to a movie file. Finally, we present two case studies using this method that examine two different cellular systems—the human platelet and the human red blood cell (RBC)—to show how visualizing existing data can provide new insights into cellular metabolism. The result are two animated videos that give detailed information about the systems under study and highlight new insights that were not previously apparent. We hope that this method will aid researchers in visualizing, perceiving, and interpreting complex data sets.

## Results

The idea of GEM-Vis is that time series can be adequately observed in the form of an animated sequence of a dynamically changing network map when using an appropriate representation of metabolic quantities. To this end, our technique exploits the repeatedly observed ability of human beholders to estimate quantities most precisely when these are mapped to a lengths scale [31]. Since metabolic maps commonly represent nodes with circles [6, 34], we suggest using the fill level of each node as a visual element to represent its amount at each time point. We experimented with visualization of data in several different ways, based on node size, color, a combination of size and color, or fill level (Supplementary Figure S1). Each of these visual representations provides some advantages over the others, but according to [31] the notion of the fill level of a node can be the most intuitive as it allows for the user to understand and gauge its minimum or maximum value quickly (see Discussion).

**Fig. 1:**
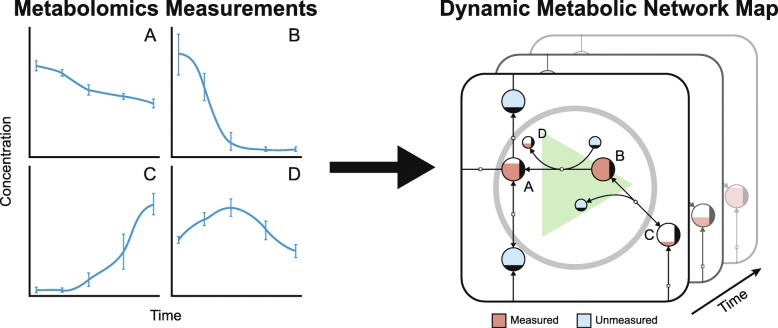
Dynamic visualization of metabolomic data. We take metabolomic data as input and generates a dynamic animation of the data over time which enables the visualization of pool sizes for individually measured metabolites. Several different options are discussed in this article for the visualization of the data based on node size, color, and fill level. The method has been implemented in SBMLsimulator including an export function to save the resulting output in a video file. For creation of animation videos highlighted in Tables 1 and 2 post-processing steps are needed as descibed in the Supplementary Information

Using this technique, we created such an animation for given longitudinal metabolomic data and a metabolic network map that corresponds to the observed cell type (Fig. 1). To provide a smooth animation, additional time points are interpolated in the provided time series. Further details regarding the development and use of the implementation of the method can be found in the Supplementary Information.

**Table 1:** Visualization of biochemical processes – storage of platelets  8 min 26 s

This video introduces a new method for visualizing metabolic processes in the context of a full biochemical network. Representing the metabolic network as a graph where metabolites are nodes and reactions are edges can help elucidate complex relationships within the network. While viewing a network in this manner is not new, overlaying -omics data onto the map allows for an accurate integration of disparate data types. By visually interpreting the information in this dynamic, graphical format, we can more easily distinguish important characteristics of the network. This video utilizes the metabolomic data from the study “Comprehensive metabolomic study of platelets reveals the expression of discrete metabolic phenotypes during storage” [13]. https://youtu.be/ GQuT7R-ldS4

**Table 2:** Visualization of biochemical processes – temperature dependence of red blood cells  1 min 33 s

This video visually compares the biochemical effects of increasing the storage temperature from (4^∘^C to 13^∘^C) of stored RBCs on metabolic processes. The relative node size shows changes in metabolite concentrations for each measured metabolite. Zooming in on various parts of the network helps visualize how specific metabolite pools undergo more drastic changes at different points during storage. This video utilizes the metabolomic data from the study “Quantitative time-course metabolomic in human red blood cells reveal the temperature dependence of human metabolic networks” [14]. https://youtu.be/0INItST4FQc	

To demonstrate the utility of this method, we applied these visualization methods to four different cellular systems—human hepatocytes [35], platelets [36] and RBCs [37], as well as to yeast [38]. For the two human blood cell types and for yeast longitudinal quantitative data sets were available in the literature [13, 14, 39]. Consequently, all four models provide very different use-case scenarios. Since the hepatocyte model [35] is a fully-specified kinetic model and available in SBML format from BioModels database [40], it is well suitable to demonstrate how simulated data can be generated and visualized in the context of an algorithmically generated network (see Additional file 11). The genome-scale model of yeast [38] can be downloaded in SBML and JSON format from BiGG Models Database [41], where it comes with a manually drawn network of the organism’s central carbon metabolism. It is, therefore, usable to demonstrate mapping a published time-course metabolomic data set [39] in the context of a hand-made pathway map (see Additional file 13).

After gaining experience in working with the visualization method, the focus will be applying the GEM-Vis method to study human blood cells in more detail. Transfusion medicine plays a vital role in modern healthcare, making the storage of different blood components important physiological processes to understand. In particular, platelets and RBCs represent relatively simple human cell types that can be intensely studied in the well-defined, static environment provided by blood storage (packed in plastic bags and stored at 22^∘^C and 4^∘^C for platelets and RBCs, respectively). While the cells are stored in these conditions, biochemical and morphological changes occur (the “storage lesion”) that are well-studied through the use of metabolomic data [12, 42]. Metabolic models were previously available for both the platelet [36] and RBC [37], enabling the creation of network maps for both reconstructions. Thus, these data could be visualized in the context of the entire metabolic network.

**Fig. 2:**
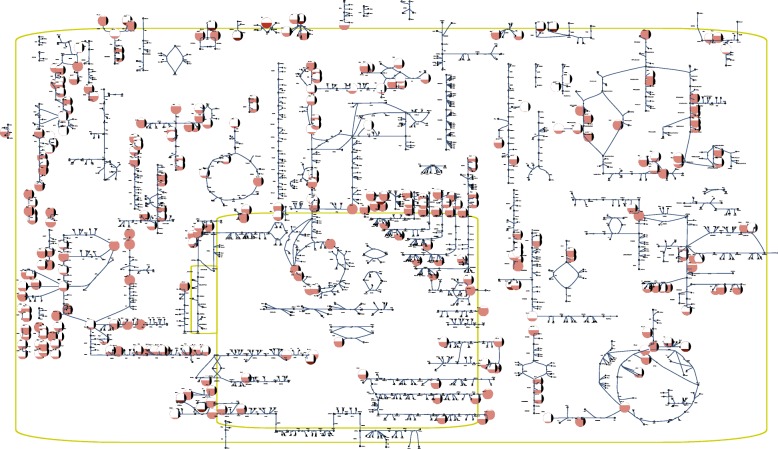
Network map in SBGN style [43] for the human platelet with metabolomic data [13] overlaid. This figure represents a visualization in which the fill level of a node represents the relative size of the corresponding metabolite pool

*Case study: human platelets under storage conditions* Our first case study examined the storage of platelets. We manually created a metabolic map for the complete metabolic network of the platelet using Escher [6]. We then overlaid metabolomic data which characterized the baseline storage conditions with eight time points over ten days of storage [13] to produce a network-level visualization of the data (Fig. 2). Using this network-level visualization, we examined the dynamics of the platelet metabolome.

During the first part of storage, stress due to the non-physiological conditions of storage (i.e., packed in a plastic bag at 22^∘^C) slows metabolic activity through glycolysis, the pentose phosphate pathway, and purine salvage pathways [13]. Several metabolites are secreted by the cells and accumulate in the storage media, such as hypoxanthine. The metabolite 5-Phospho- *α*-D-ribose 1-diphosphate (PRPP) is produced from the pentose phosphate pathway and is a cofactor in the salvage reactions that break down hypoxanthine. Because flux through the pentose phosphate pathway is lower, the cells have less capacity to recycle hypoxanthine using the salvage pathways.

By viewing all of the data simultaneously at the network level, we were able to discover that the concentration profile of nicotinamide mirrors that of hypoxanthine. This observation suggests a similar rationale for the accumulation of nicotinamide, providing a hypothesis as to why the salvage pathway within purine metabolism has lower activity during the first few days of storage. These findings are demonstrated in the video highlighted in (Table 1), helping show how network-level visualization allows for improved extraction of biological insight from large, complex data sets.

**Fig. 3:**
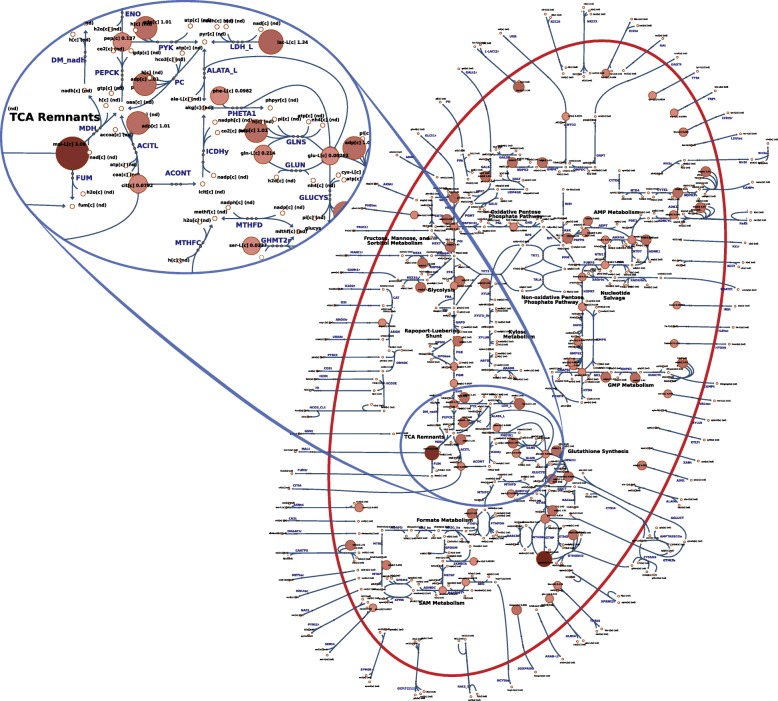
Overview of the RBC metabolic network under storage conditions at 4^∘^C. The size and color of the nodes reflects their absolute abundance. The oval area on the top magnifies a region in the center of the map that appears in the style of Escher [6] in contrast to the SBGN style shown in Fig. 2

*Case study: human red blood cells under storage conditions* Our second case study examined the storage of RBCs. A metabolic map was already available for the RBC [44] and captures the complete metabolic network [37]. Here, we sought to examine a data set that provided the opportunity to visualize different experimental conditions for the same network. Recently, a study was published [14] that used quantitative longitudinal metabolomic data to examine the state of the RBC metabolome under four different storage temperatures: 4^∘^C (storage temperature), 13^∘^C, 22^∘^C, and 37^∘^C (body temperature). For this system, we opted to visualize the dynamics of the metabolite concentrations as nodes with variable size where smaller nodes represent smaller pool sizes, and larger nodes represent larger pool sizes (Fig. 3).

To highlight the differences between the experimental conditions, we examined two of the conditions side-by-side (see the video highlighted in Table 2). This visualization helps supplement the type of statistical and modeling analyses performed previously and helps contextualize the effects of the temperature change across different parts of the network. In particular, it is obvious from a network-level view of the system that certain parts of the network are more active at different points in the time-course. A side-by-side comparison helped emphasize that the availability of reduced glutathione is different with increased temperature, an important physiological feature due to the role of glutathione in neutralizing reactive oxygen species [45] that accumulate during storage and contribute to the storage lesion [46]. Finally, it can be seen that hypoxanthine—a known toxic metabolite whose concentration has been shown to inversely correlate with the post-transfusion recovery rates of transfusion patients [47]—accumulates faster at higher temperatures. Like in the other case study presented above, the new insights into complex processes (which are not yet fully understood) provide evidence that this method can be beneficial for the simplification and understanding of large, complex data analyses.

## Discussion

In this article, we proposed GEM-Vis as a new method for visualizing time-course metabolomic data in the context of large-scale metabolic networks. The approach was evaluated with a range of different use-cases, ranging from the display of simulated data on automatically generated network layouts to experimentally obtained metabolite concentration data on manually drawn network maps. All experiments were described in elaborate tutorial videos (see supplementary material). Subsequently, the method was applied to study two different cases of human blood cells (platelets and erythrocytes) in more detail.

As a result, a network-level representation of large metabolomic data sets presents a more holistic view of the data than does statistical analysis alone. While visual inspection of data is indeed not a replacement for more detailed statistical or modeling analyses, this method provides an important supplement to existing data analysis pipelines. We demonstrate its utility in such an analysis pipeline by highlighting findings from existing data sets [13, 14]. Visualizing the metabolomic data in the context of the full metabolic network allowed for new insights into existing data sets. A potential explanation why the salvage pathway lowers its activity during the first few days of platelet storage could be deduced for the network of the human platelet. In the RBC network, it could easily be seen that concentrations in certain parts of the network (e.g., nucleotide metabolism) accumulated or depleted together. These findings illustrate the promising potential of visualized time-course data and—combined with in-depth computational data analysis—can help perceiving information and elucidate physiological processes.

The simplification of experimental data interpretation became extremely relevant in the age of high-throughput technologies. The visualization concept presented here offers a systems-level interpretation of metabolomic data. Combined with other data analytics, this method helps provide a holistic view of a data set, moving us closer to being able to realize the full potential of a given data set. More broadly, we hope that the method presented here will provide the starting point for further visualization improvements not only for metabolomic data but for the visualization and contextualization of other data types. Future work may include combining a dynamic representation with static concentration graphs that will continue to improve the capabilities of such software to fully meet the needs of life science researchers.

## Methods

The method described in this paper utilizes existing software libraries to visually represent metabolomic data in the context of a metabolic network map.

**Fig. 4:**
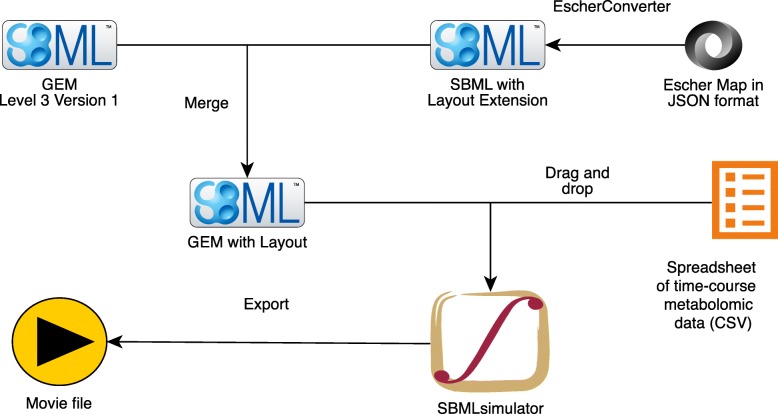
Creation of an animated video from an SBML file, a pathway map, and a time-course metabolomic data set. EscherConverter converts the manually drawn pathway map in Escher’s JSON format [6] to SBML [33] with Layout extension [32]. The resulting SBML file is merged with the corresponding GEM (in SBML Level 3 Version 1 format, e.g., using the Java code from Additional file 14). After opening the merged SBML file in SBMLsimulator [48], a time-course metabolomic data set in CSV format (character-separated values) is also loaded to SBMLsimulator. An export function is provided in SBMLsimulator to generate a dynamic time-course animation

In brief, the metabolic map must be embedded as SBML Layout extension (version 1) into an SBML Level 3 Version 1 file that is provided to the software SBMLsimulator [48]. In this study, the design of metabolic network maps was created using the web-based software Escher [6] within the web browser Safari 11 and stored in JSON format, resulting in Additional file 4 for *i*AT-PLT-636 and Additional file 8 for *i*AB-RBC-283. Subsequently, the generated maps have been converted to SBML using the software EscherConverter (available at https://github.com/draeger-lab/EscherConverter/) and embedded into the metabolic model using a custom Java™ program (Additional file 14). The resulting SBML file with embedded layout for *i*AT-PLT-636 can be found in Additional file 5, and in Additional file 9 for *i*AB-RBC-283 (both files are compressed using GZIP). The metabolomic time-course data are provided to SBMLsimulator in a *.csv file format with identifiers matching those of the map (Additional file 3 for *i*AT-PLT-636 and Additional file 7 for *i*AB-RBC-283, the latter is a compressed ZIP archive). SBMLsimulator reads in the SBML file with embedded layout and the time-course data. Subsequently, SBMLsimulator uses splines to interpolate the data over time with input from the user. Other features are selected, such as the speed of animation and how metabolite concentrations are represented (e.g., fill level). An optional *.csv file can be provided to SBMLsimulator to define a moving camera animation. To this end, this CSV file needs to contain as the first value the zoom level of the camera followed by a tab-separated list of corner points along the way of the moving camera (these points are the top left corners of the camera’s view port). The result is a smooth animation that allows features such as zooming and panning across different areas of the map, which the user can safe to a video file in one of the supported formats, e.g., AVI, MP4, MPG, WMV, FLV, or MOV. The procedure is depicted in Fig. 4 and demonstrated in detail in Additional files 10, 11, 12 and 13.

Additional file 10This tutorial video (36.3 MB) demonstrates how to download, installation, and launch the software SBMLsimulator. The video is available at https://youtu.be/Eu4uSPmNXVI.

Additional file 11This tutorial video (61.8 MB) demonstrates how to load model files in SBML format and how to run a simulation using the software SBMLsimulator. The video is available at https://youtu.be/CVzp_XtIaHU.

Additional file 12This tutorial video (66.7 MB) demonstrates how to embedding a model layout in an SBML file and how to prepare experimental data for loading it into the software SBMLsimulator. The video is available at https://youtu.be/CoeOh2sFFSQ.

Additional file 13This tutorial video (52.9 MB) demonstrates how to visualize manually created layouts using the software SBMLsimulator. The video is available at https://youtu.be/qv3qPyzofhI.

SBMLsimulator is implemented in Java™ SE 8 under macOS High Sierra on a MacBook Pro 15”, 2016. All computation for the animation videos has been performed under macOS High Sierra version 10.13.2. The animation videos for the two case studies were created using Windows 10.

Audio recording was performed using a ZOOM Handy Recorder H4 and a Steinberg UR22 mkII USB Audio Interface 24 bit/192 kHz (Steinberg Media Technologies GmbH, Hamburg, Germany) in combination with a Røde NT1-A (Røde, Silverwater, NSW, Australia), and the recording software Quicktime (Apple Inc., Cuppertino, CA, USA). Sony VEGAS Pro (version 12) was used for video post-processing, resulting in Additional files 2 and 6.

Full details for the implementation and use of the software are provided in the Supplemental Material (Additional files 1 and 15).

## Supplementary information

**Additional file 1** Supplementary information about details of the method and implementation can be found in file 12859_2020_3415_MOESM1_ESM.pdf.

**Additional file 2** The animated movie iAT-PLT-636.mp4 (547.4 MB) about *i*AT-PLT-636 available at https://youtu.be/GQuT7R-ldS4.

**Additional file 3** The data set iAT-PLT-636_Data.csv used in the *i*AT-PLT-636 animation (15 kB).

**Additional file 4** The pathway map in Escher format iAT-PLT-636_Map.json.gz compressed with gzip (200 kB).

**Additional file 5** The pathway map in SBML Level 3 Version 1 format with Layout and FBC (flux balance constraints) packages iAT-PLT-636_Map.xml.gz compressed with gzip (457 kB).

**Additional file 6** The animated movie iAB-RBC-283.mp4 (118.9 MB) about *i*AB-RBC-283 available at https://youtu.be/0INItST4FQc.

**Additional file 7** The data set iAB-RBC-283_Data.zip used in the *i*AB-RBC-283 animation (13 kB).

**Additional file 8** The pathway map in Escher format iAB-RBC-283_Map_cell-outline.json.gz (94 kB).

**Additional file 9** The pathway map iAB-RBC-283_Map_cell-outline.xml.gz in SBML Level 3 Version 1 format with Layout and FBC packages compressed with gzip (153 kB). Please note that the core model in this file has been reduced to the content of the pathway map and does not comprise all reactions and metabolites of the original model by [37].

**Additional file 14** A brief Java™ program that embeds a metabolic map in the format of SBML Layout extensions into an SBML Level 3 Version 1 file.

**Additional file 15** This document describes the features of the application SBMLsimulator and explains how to use them.

## Data Availability

Data and materials required to reproduce the findings in this article are freely available. See the appendix in Additional file 1 for details. For post-processing generated animation files, a commercial video editing software, such as Sony VEGAS Pro (version 12) that was used for creating Additional files 2 and 6, can be obtained for purchase. Computers and audio recording devices need to be purchased.

## Abbreviations

AVI: Audio video interleave
BiGG: Biochemically, genetically, genomically structured
CSV: Character-separated values
FLV: Flash video
GEM: Genome-scale metabolic model
GEM-Vis: GEM-visualization
GZIP: GNU zip
JSON: JavaScript object notation
MOV: QuickTime file format
MPG: Moving pictures expert group
MP4: Multimedia file format
PRPP: 5-phospho- *α*-d-ribose 1-diphosphate
RBC: Red blood cell
SBML: Systems biology markup language
WMV: Windows media video
