# Supplementary material for: Visualizing metabolic network dynamics through time-series metabolomic data
Source: BMC Bioinformatics. 2020 Jul 7;21:130. doi: 10.1186/s12859-020-3415-z (PMC7119163; doi:10.1186/s12859-020-3415-z)
Supplement: Supplementary file 5 — Additional file 1 Supplementary information about details of the method and implementation can be found in file 12859_2020_3415_MOESM1_ESM.pdf. [file 12859_2020_3415_MOESM1_ESM.pdf]

Supplementary Material

Visualizing metabolic network dynamics through time-series metabolomic data

Lea F. Buchweitz[1,*], James T. Yurkovich[2,*], Christoph M. Blessing[1,3], Veronika Kohler[1,3], Fabian Schwarzkopf[4], Zachary A. King[5,6], Laurence Yang[7], Freyr Jóhannsson[8], Ólafur E. Sigurjónsson[9,10], Óttar Rolfsson[8], Julian Heinrich[3], and Andreas Dräger[1,3,11,*]

[1]Computational Systems Biology of Infection and Antimicrobial-Resistant Pathogens, Institute for Biomedical Informatics (IBMI), Sand 14, 72076 Tübingen, Germany. [2]Institute for Systems Biology, 401 Terry Ave. N., WA 98109 Seattle, United States [3]Department of Computer Science, University of Tübingen, Sand 14, 72076 Tübingen, Germany [4]yWorks GmbH, Vor dem Kreuzberg 28, 72070 Tübingen, Germany [5]Systems Biology Research Group, Department of Bioengineering, University of California, San Diego, 9500 Gilman Drive, CA 92093-0412 La Jolla, United States [6]Novo Nordisk Foundation Center for Biosustainability, Technical University of Denmark, Building 220, Kemitorvet, 2800 Kgs.Lyngby, Denmark [7]Department of Chemical Engineering, Queen's University, ON K7L 3N6 Kingston, Canada [8]Center for Systems Biology, University of Iceland, Sturlugata 8, 101 Reykjavík, Iceland [9]The Blood Bank, Landspítali-University Hospital, 101 Reykjavík, Iceland [10]School of Science and Engineering, Reykjavík University, Menntavegi 1, 101 Reykjavík, Iceland [11]German Center for Infection Research (DZIF), partner site Tübingen [*]Authors with equal contribution

January 4, 2020

Supplementary Methods

Changing node size

Metabolites are graphically represented as circles—a standard practice in Systems Biology Graphical Notation (SBGN) (Le Novère et al., 2009)—but with variable size according to the quantitative value of a data point in the experimental data. The maximum and minimum size of a metabolite is determined automatically by the maximum and minimum measured value in the given experimental data. Based on these numbers, the quantity of the metabolite at each specific time point is taken to calculate the size of the circle. This approach has been found to be intuitive because a larger area can be readily associated with more abundant quantity (Cleveland and McGrill, 1984).

The size of the metabolites can represent either the absolute quantity of one metabolite itself or the relative quantity of this metabolite compared to all others. For more in-depth analysis the absolute quantity seems to be more helpful, however. For instance, if the data points represent metabolic concentration, small changes in concentration would not impart massive changes in node size over time, thereby aiding visualization by allowing significant changes in metabolites' concentration to be more easily identified within the network.

Changing node color

The visualization mode of changing node colors encodes the quantitative values of the experimental data in a color gradient. Either three different colors or three different shadings of one or two colors can be pre-defined to represent the maximum, middle, and minimum measured value of a metabolite. The algorithm calculates the color of all values in-between from the color gradients. Compared to the visualization mode mentioned before, this avoids the problem of overlapping metabolites if the network is not dynamically and simultaneously rearranged.

As already mentioned for the visualization mode of changing node sizes, either the absolute or the relative value of a metabolite can be represented in this mode. Depending on the analysis task both versions can be useful, but the mainly used version will be the representation of the absolute value of a metabolite as the node color.

Combination of changing node size with changing node color

There is also the possibility to combine both visualization modes, i.e., one mode for displaying global differences and one for individual changes. Either combination of relative or absolute visualization of the color or size can be utilized at the same time for the investigation of metabolic changes. This offers a more holistic insight into the data set used and can highlight dynamics that may be overseen otherwise. This enables the investigation of relative and absolute metabolic changes at the same time, which offers a more holistic insight into the used data set.

Changing fill levels

In the third visualization type, metabolites are visualized as round "aquariums" showing the corresponding data points as a "fill level of water." Compared to the other two visualization modes, this mode combines the representation of absolute and relative values in one graphical presentation. While the fill levels show the absolute measured data points of one metabolite, the color of the fill level representation refers to the relatively measured data points of this metabolite compared to all others.

As depicted in Supplementary Figure S1e a fully colored metabolite describes the maximum measured value (full fill level), in contrast, an empty metabolite shows the minimum measured value (fill level is zero). In supplementary Figures S1e to S1f also the different colors of the involved metabolites are shown. The darker the color, the higher is the measured value of this metabolite in comparison to all others. For a lighter shading, the average value is lower (Supplementary Figure S1f). In contrast to the other types, this visualization mode offers the possibility of getting a reference of the current depicted data point to the maximum and the minimum measured value of this metabolite. For instance, by showing the absolute concentration of the metabolite as a fill level, the estimation of how close the depicted concentration is to its metabolic specific maximum or minimum is more apparent than for the other two visualization modes. According to Cleveland and McGrill (1984), the extraction of the information is also more natural than estimating area sizes or colors.

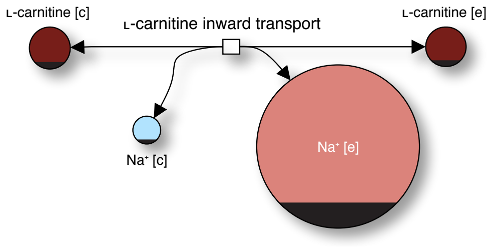

(a) Absolute color, relative size at time point 1

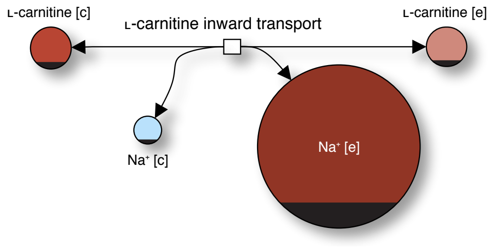

(b) Absolute color, relative size at time point 8

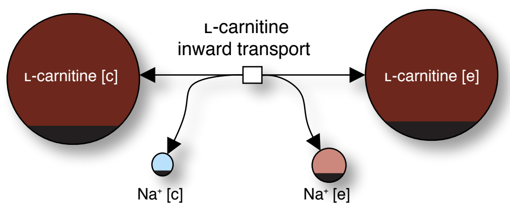

(c) Relative color, absolute size at time point 1

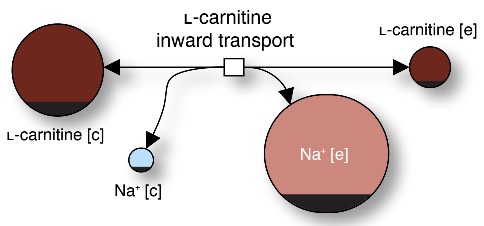

(d) Relative color, absolute size at time point 8

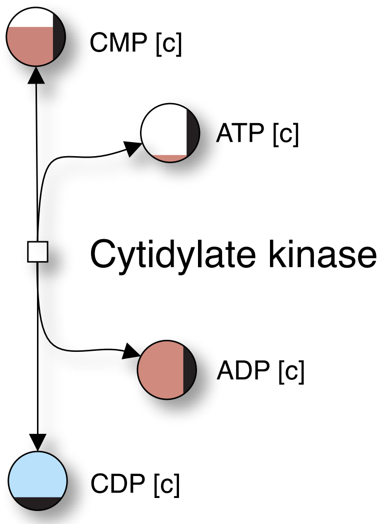

(e) Absolute concentration by fill level

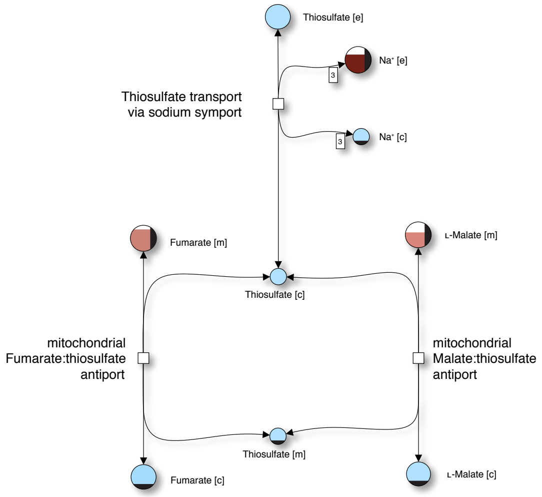

(f) Absolute fill levels combined with relative color

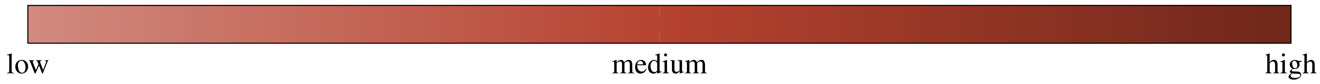

Concentration color code

Figure S1 | Combinations of color and size for representing relative and absolute concentration changes. This figure contrasts three combinations of color and size changes to represent relative concentration values and absolute quantity at two different time points of the experiment. Metabolites for which no data is available, appear in the configurable default color, here light blue. The color bar at the bottom shows the mapping between high, medium, and low concentration values of the relative metabolite concentrations.

Video-like animation

Although the experimental data only offers discrete time points with specific quantitative values, time is a continuous, dynamic entity and, therefore, an appropriate representation should be created. Hence, the discrete data points should be displayed continuously in a smooth video-like animation. This is afforded by a linear interpolation which regularly calculates the current depicted value from the given interval of the possible maximum and minimum value. Each time, the interpolation calculates a new value, the metabolites are redrawn accordingly. This permanent redrawing of the metabolites with different size, color or fill level results in a smooth, video-like movement imitating an animation. Additionally, the interval of how close the time points of the experimental data follow each other can be adjusted, which shortens or extends the duration of the entire 'animation.' It also has to be noted that one standard defined by SBGN (Le Novère et al., 2009), called *clone markers*, was changed. Species occurring more than once in a network were initially marked by a dark band along the bottom of the species. However, because these bands can be easily confused with a fill level, the dark bands were just rotated by 90° counterclockwise (see Supplementary Figures S1e to S1f). This change was only made to the metabolites displaying a fill level, e.g., metabolites which offer experimental data (see Supplementary Figures S2a to S2b).

Escher

Escher is a web-based software for drawing, manipulating, and viewing metabolic networks, implemented in JavaScript (King et al., 2015). In this work, Escher's feature for overlaying these networks with data has been extended to cover time series data (Figure S3). The source-code of this modified version of Escher is available at `/christophblessing/escher/`. Supported features include mapping of time series data to a pair of absolute node sizes and absolute colors as well as the display of animations. The current implementation maps high values to large dark nodes and low values to small bright nodes. Escher naively encodes network maps in a tool-specific JSON format.

SBMLsimulator

The software tool with which the new visualization concept was realized is called SBMLsimulator (Dörr et al., 2014) and is an open source project available at `/draeger-lab/SBMLsimulator/`. SBMLsimulator is a Java Archive file (JAR), based on Java™ 1.8, which can be run on every Java™ Virtual Machine (JVM). It is based on the open-source library JSBML (Dräger et al., 2011; Rodriguez et al., 2015) and was created for dynamic simulation and heuristic optimization of parameters of models which are written in SBML format. SBMLsimulator combines EvA2 (Streichert and Ulmer, 2005) with the Systems Biology Simulation Core Library (SBSCL) (Keller et al., 2013) for simulation and parameter estimation in one graphical user interface. See the screenshot in supplementary Figure S4.

yFiles

To visualize the given SBML format (Hucka et al., 2003) of systems biology networks the commercial Java™ library yFiles[1] version 3.0 was used. YFiles is made by yWorks and is provided for several platforms. It offers a wide range of different algorithms to analyze and visualize graphs or networks. There is also the possibility to arrange the layout of a network diagram automatically. In SBMLsimulator (Dörr et al., 2014) yFiles is used to display a given SBML model as a graph with metabolites as nodes, edges as reactions and compartments, like mitochondria, as yellow lines. For the realization of graph visualization in SBMLsimulator, the Swing implementation of yFiles was used.

Xuggle

Xuggle is an open source library which allows video and audio stream editing. This software tool is used to export the visualized animation of a network and its experimental data. For SBMLsimulator (Dörr et al., 2014) version 5.4 of Xuggle is used which encodes a video from a series of screenshots of the animation in SBMLsimulator.

[1] `https://www.yworks.com/products/yfiles-for-java`

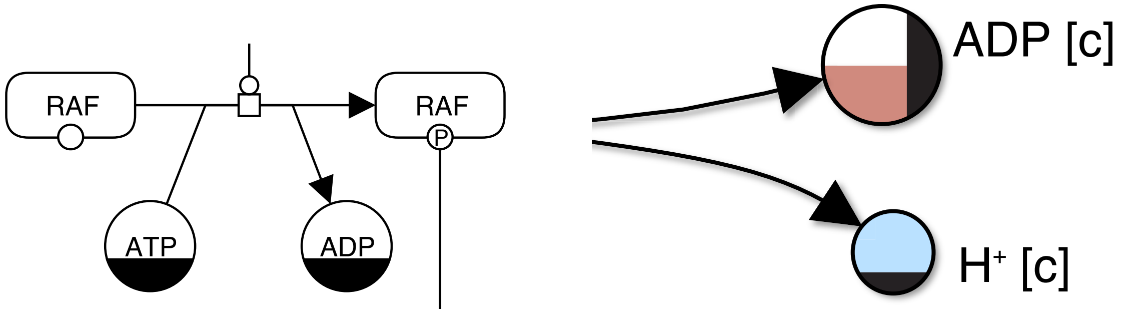

(a) SBGN standard; taken from Le Novère et al. (2009) **(b)** Changed version in this work

Figure S2 | Different clone markers. Clone markers in SBGN standard and the counterclockwise rotated version used in this study, because of the similarity of the clone markers to a filling level. The rotation of the clone marker is only performed in metabolites offering time-course data and only during the animation with filling levels. Metabolites which are not offering experimental data or animations in any other representation mode keep their clone markers according to the SBGN standard.

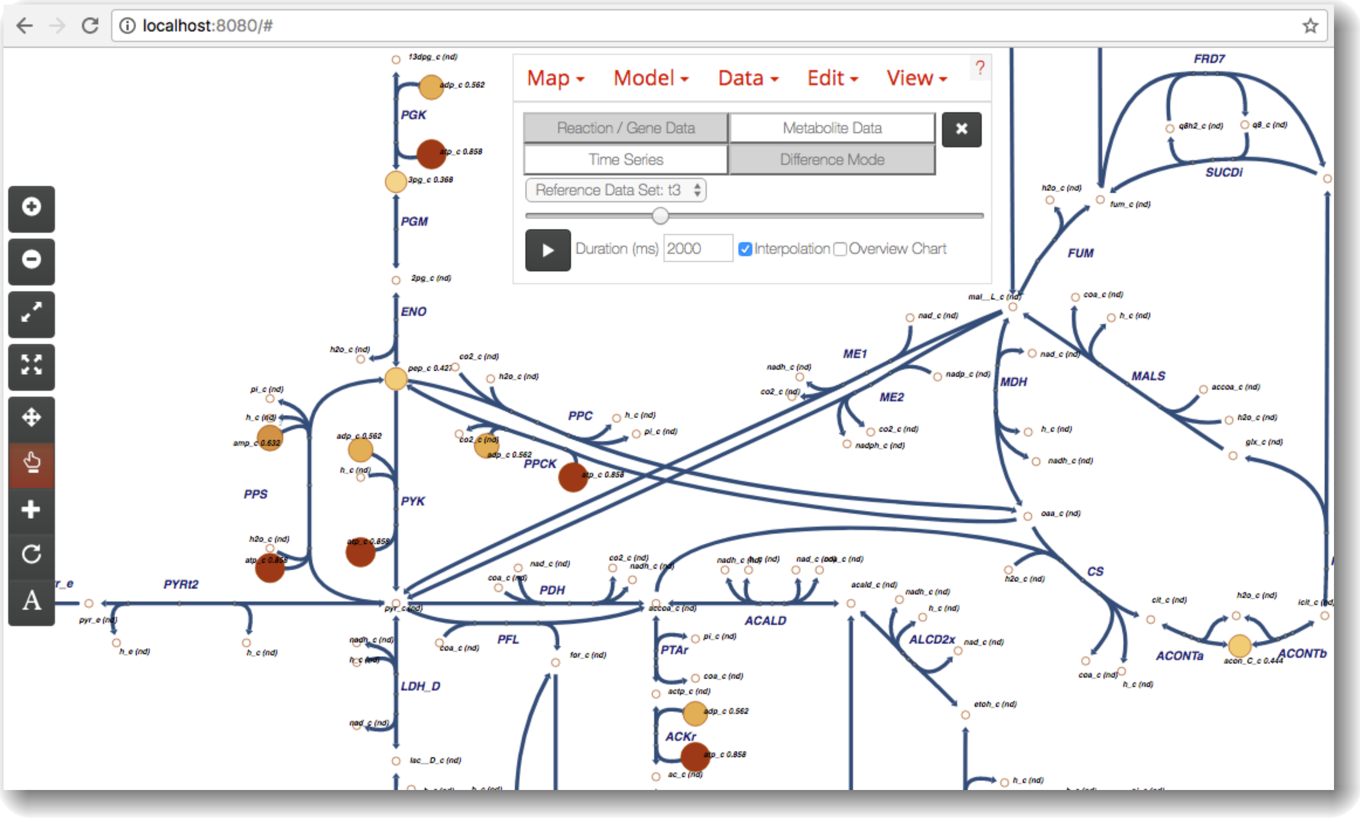

Figure S3 | Screenshot of dynamic data visualization using the web-based Escher application (King et al., 2015)

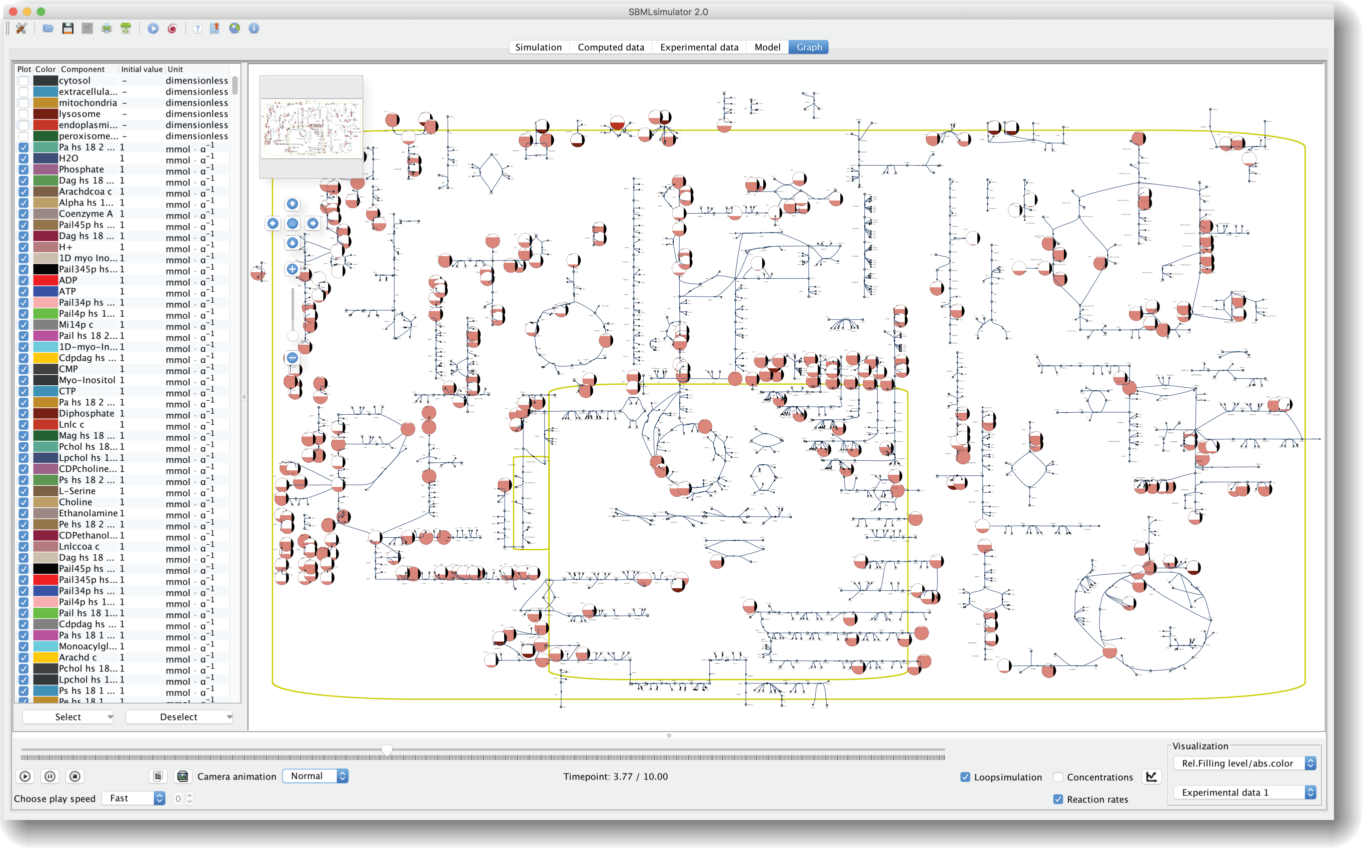

Figure S4 | Screenshot of SBMLsimulator in “Graph” tab. SBMLsimulator (Dörr et al., 2014) was the primary tool used in this work. Similarly, dynamic visualizations can now also be produced with Escher (see Figure S3).

Sony VEGAS Pro 12

Sony VEGAS Pro 12.0 is a professional video and audio editing program provided by Sony. It is a standalone commercial setup for Windows, here used under Windows 10. Its design is easy to grasp for novices but also has a wide range of powerful features which are also used by experienced users. Sony VEGAS Pro accompanies the whole creation process of a video. The currently latest version is Sony VEGAS Pro 14.0. Sony VEGAS Pro 12.0 has been exclusively used to create the videos of this study (see Supplementary Figure S5).

ZOOM Handy Recorder H4

The ZOOM Handy Recorder H4 is an all-in-one recorder which combines a handy size with a high-performance stereo condenser microphone, a Secure Digital (SD) card recorder, a mixer, an effect section and some more features. It is possible to switch between the stereo mode and a 4-track mode which provides the playback of four simultaneous tracks and the recording of two tracks. There are several sampling rates to choose, the tracks are recordable in WAV or MP3 format, and the device is easily connected to the computer via SD. All audio tracks used in the videos are recorded with the ZOOM Handy Recorder H4.

Network models

*i*AT-PLT-636

The first model used as one application example is a cell-scale, charge, and mass balanced biochemical network reconstruction of the human platelet with 1008 reactions (including biochemical transformations as well as intra-cellular and extracellular transporters), 225 proteins, 636 genes, and 738 compartment-specific metabolites, de-

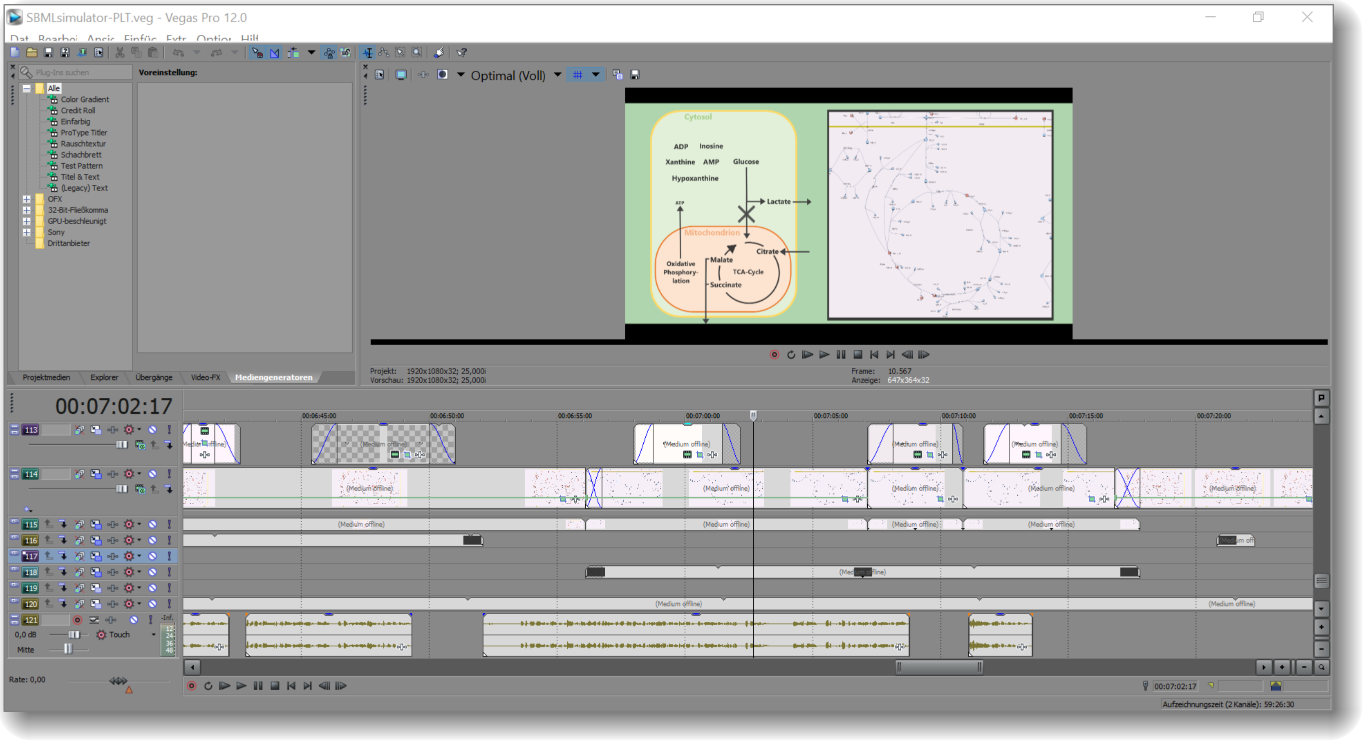

Figure S5 | Screenshot of Sony VEGAS Pro 12.0. This program was used exclusively for video and audio editing

picted in this network. The model was constructed with the primary aim to offer a scaffold with which further data-driven examination could bring deeper insight into the influence of platelets on human diseases. The exact layout used for this work was uniquely created for this study and is available as supplementary material of this article. The model is freely available on the BiGG Models database (King et al., 2016).

*i*AB-RBC-283

For the red blood cell visualization, we used a modified version of the erythrocyte metabolic reconstruction *i*AB-RBC-283 (Bordbar et al., 2011), which had been previously used for building personalized kinetic models (Bordbar et al., 2015). The model *i*AB-RBC-283 is a full bottom-up created reconstruction of the human red blood cell with 292 intracellular reactions, 77 transporters, 267 unique metabolites and accounts for 283 metabolic genes, which suggest that the metabolic role of erythrocytes is much more diverse and expansive than previously presumed. An existing layout of the (human) red blood cell (RBC) metabolic network was used (Yurkovich et al., 2017a). The model is freely available on the BiGG Models database (King et al., 2016).

Preparation of network layouts

All systems biology networks used for the input of this study maps were manually drawn using Escher (King et al., 2015) and stored the native JSON-based format of this software. The stand-alone program EscherConverter[2] converted these files maps to SBML format (Hucka et al., 2003) Level 3 Version 1 Release 2 (Hucka et al., 2015) with layout information (Gauges et al., 2006, 2015) and flux balance constraints (Ollivier and Bergmann, 2018). The annotation application ModelPolisher[3] merged the resulting maps with knowledge from BiGG Models Database (King et al., 2016; Römer et al., 2016).

[2] `/draeger-lab/EscherConverter/`
[3] `/draeger-lab/ModelPolisher/`

Experimental data

The experimental data which was used to create the videos of the new time visualization modes were in CSV format structured as a table. The first column defines the time, and all others are labeled with the identifier of the corresponding metabolite. The rows identify the different measured time points.

Experimental data used for *i*AT-PLT-636

As experimental data of human platelets, which can be visually analyzed, a metabolomics study was chosen in which various quantitative, but no graphical visualization methods were used for examination. Paglia et al. (2014) investigated the over-time change in metabolism of stored platelet concentrations. Because of several beneficial clinical applications, the storage of Platelet Concentration (PC) at the highest quality possible is worthwhile. However, during storage, the (human) platelet (PLT) quickly suffer from a high-quality loss. This process of metabolic decay is called the Platelet Storage Lesion (PSL). As their primary result, Paglia et al. could distinguish three different metabolic states during PSL. The state of short-term stored PCs (day 0-3) is mainly characterized by the appearance of a different microenvironment compared to the initial compound. This is caused by the accumulation of different acidic metabolites secreted into the storage solution, i.e., succinate and malate. Consequential to the accumulation of acidic substances a decrease in pH value follows which marks a significant transition point in platelet metabolism. The second state (day 4-6) is apparent in medium-term stored PCs which show moderate mitochondrial metabolism activity and increased ATP production to sustain PLT metabolism as long as possible. In the extreme state of long-term stored platelets (day 7-10) probably irreversible changes occur which then lead to a faster decay and total cell lysis. Considering all phenotypes the decline of platelet metabolism is a nonlinear process consisting of successive metabolic shifts.

Experimental data used for *i*AB-RBC-283

We used the quantitative metabolomic data from Yurkovich et al. (2017b) that examines the dynamics of RBCs under storage conditions at different temperatures. In this study, systems biology is used to fill the gap between temperature dependency studies on a biochemical and on a physiological level. The goal of Yurkovich et al. was to examine temperature dependencies of metabolism in red blood cells. They used a deep-coverage, quantitative, time-course metabolomics of human red blood cells in storage and investigated metabolic changes under four different temperature conditions: 4 °C (storage temperature), 13 °C, 22 °C, and 37 °C (body temperature). Studying temperatures in this range provides a baseline data with no transcriptional or translational regulation (e.g., red blood cells do not have genetic material), which offers insights into temperature dependence in a broader context.

Abbreviations

ATP Adenosine triphosphate

BiGG Biochemically, genetically, and genomically structured

CSV Character-Separated Value

JAR Java Archive file

JVM Java™ Virtual Machine

JSON JavaScript Object Notation

PC Platelet Concentration

PLT (human) platelet

PSL Platelet Storage Lesion

RBC (human) red blood cell

SBGN Systems Biology Graphical Notation

SBML Systems Biology Markup Language

SBSCL Systems Biology Simulation Core Library

SD Secure Digital

WAV Waveform Audio File Format

References

Aarash Bordbar, Neema Jamshidi, and Bernhard O. Palsson. *i*AB-RBC-283: A proteomically derived knowledge-base of erythrocyte metabolism that can be used to simulate its physiological and patho-physiological states. *BMC Systems Biology*, 5(1):110, July 2011. ISSN 1752-0509. doi:10.1186/1752-0509-5-110. URL `http://www.biomedcentral.com/1752-0509/5/110`.

Aarash Bordbar, Douglas McCloskey, Daniel Zielinski, Nikolaus Sonnenschein, Neema Jamshidi, and Bernhard O. Palsson. Personalized Whole-Cell Kinetic Models of Metabolism for Discovery in Genomics and Pharmacodynamics. *Cell Systems*, 1(4):283–292, October 2015. ISSN 24054712. doi:10.1016/j.cels.2015.10.003. URL `http://linkinghub.elsevier.com/retrieve/pii/S2405471215001490`.

William S. Cleveland and Robert McGrill. Graphical Perception: Theory, Experimentation and Application to the Development of Graphical Methods. *Journal of the American Statistical Association*, 79(387):531–554, September 1984. URL `http://www.jstor.org/stable/2288400`.

Alexander Dörr, Roland Keller, Andreas Zell, and Andreas Dräger. SBMLsimulator: A Java Tool for Model Simulation and Parameter Estimation in Systems Biology. *Computation*, 2(4):246–257, 2014. ISSN 2079-3197. doi:10.3390/computation2040246. URL `http://www.mdpi.com/2079-3197/2/4/246`.

Andreas Dräger, Nicolas Rodriguez, Marine Dumousseau, Alexander Dörr, Clemens Wrzodek, Nicolas Le Novère, Andreas Zell, and Michael Hucka. JSBML: a flexible Java library for working with SBML. *Bioinformatics*, 27(15):2167–2168, June 2011. doi:10.1093/bioinformatics/btr361. URL `http://bioinformatics.oxfordjournals.org/content/27/15/2167`.

Ralph Gauges, Ursula Rost, Sven Sahle, and Katja Wegner. A model diagram layout extension for SBML. *Bioinformatics*, 22(15):1879–1885, May 2006. doi:10.1093/bioinformatics/btl195. URL `http://bioinformatics.oxfordjournals.org/cgi/content/abstract/22/15/1879`.

Ralph Gauges, Ursula Rost, Sven Sahle, Katja Wengler, and Frank T. Bergmann. The Systems Biology Markup Language (SBML) Level 3 Package: Layout, Version 1 Core. *Journal of Integrated Bioinformatics*, pages –, 2015. doi:10.2390/biecoll-jib-2015-267.

Michael Hucka, Andrew Finney, Herbert M Sauro, Hamid Bolouri, John C Doyle, Hiroaki Kitano, Adam P Arkin, Benjamin J Bornstein, Dennis Bray, Athel Cornish-Bowden, Autumn A Cuellar, Sergey Dronov, Ernst Dieter Gilles, Martin Ginkel, Victoria Gor, Igor I Goryanin, Warren J Hedley, T Charles Hodgman, Jan-Hendrik S Hofmeyr, Peter J Hunter, Nick S Juty, Jay L Kasberger, Andreas Kremling, Ursula Kummer, Nicolas Le Novère, Leslie M Loew, Daniel Lucio, Pedro Mendes, Eric Minch, Eric D Mjolsness, Yoichi Nakayama, Melanie R Nelson, Poul F Nielsen, Takeshi Sakurada, James C Schaff, Bruce E Shapiro, Thomas Simon Shimizu, Hugh D Spence, Jörg Stelling, Koichi Takahashi, Masaru Tomita, John M Wagner, Jian Wang, and the rest of the SBML Forum. The systems biology markup language (SBML): a medium for representation and exchange of biochemical network models. *Bioinformatics*, 19(4):524–531, March 2003. doi:10.1093/bioinformatics/btg015. URL `http://bioinformatics.oxfordjournals.org/cgi/content/abstract/19/4/524`.

Michael Hucka, Frank T. Bergmann, Andreas Dräger, Stefan Hoops, Sarah M. Keating, Nicolas Le Novère, Chris J. Myers, Brett G. Olivier, Sven Sahle, James C. Schaff, Lucian P. Smith, Dagmar Waltemath, and Darren J. Wilkinson. Systems Biology Markup Language (SBML) Level 2 Version 5: Structures and Facilities for Model Definitions. *Journal of Integrated Bioinformatics*, page 271, 2015. doi:10.2390/biecoll-jib-2015-271. URL `http://journal.imbio.de/article.php?aid=271`.

Roland Keller, Alexander Dörr, Akito Tabira, Akira Funahashi, Michael J. Ziller, Richard Adams, Nicolas Rodriguez, Nicolas Le Novère, Noriko Hiroi, Hannes Planatscher, Andreas Zell, and Andreas Dräger. The systems biology simulation core algorithm. *BMC Systems Biology*, 7(55):55, July 2013. doi:10.1186/1752-0509-7-55. URL `http://www.biomedcentral.com/1752-0509/7/55`.

Zachary A King, Andreas Dräger, Ali Ebrahim, Nikolaus Sonnenschein, Nathan E Lewis, and Bernhard O Palsson. Escher: A web application for building, sharing, and embedding Data-Rich visualizations of biological pathways. *PLoS Comput. Biol.*, 11(8):e1004321, August 2015.

Zachary A. King, Justin S. Lu, Andreas Dräger, Philip C. Miller, Stephen Federowicz, Joshua A Lerman, Ali Ebrahim, Bernhard O. Palsson, and Nathan E. Lewis. BiGG Models: A platform for integrating, standardizing and sharing genome-scale models. *Nucleic Acids Research*, 44(D1):D515–D522, January 2016. doi:10.1093/nar/gkv1049. URL `http://nar.oxfordjournals.org/content/44/D1/D515`.

Nicolas Le Novère, Michael Hucka, Huaiyu Mi, Stuart Moodie, Falk Schreiber, Anatoly Sorokin, Emek Demir, Katja Wegner, Mirit I. Aladjem, Sarala M. Wimalaratne, Frank T. Bergmann, Ralph Gauges, Peter Ghazal, Hideya Kawaji, Lu Li, Yukiko Matsuoka, Alice Villéger, Sarah E. Boyd, Laurence Calzone, Melanie Courtot, Ugur Dogrusoz, Tom C. Freeman, Akira Funahashi, Samik Ghosh, Akiya Jouraku, Sohyoung Kim, Fedor Kolpakov, Augustin Luna, Sven Sahle, Esther Schmidt, Steven Watterson, Guanming Wu, Igor Goryanin, Douglas B. Kell, Chris Sander, Herbert Sauro, Jacky L. Snoep, Kurt Kohn, and Hiroaki Kitano. The Systems Biology Graphical Notation. *Nature Biotechnology*, 27(8):735–741, 2009. ISSN 1087-0156. doi:10.1038/nbt.1558. URL `https://www.nature.com/articles/nbt.1558`.

Brett G. Ollivier and Frank T. Bergmann. SBML Level 3 Package: Flux Balance Constraints version 2. *Journal of Integrative Bioinformatics*, March 2018. doi:10.1515/jib-2017-0082. URL `https://www.degruyter.com/view/j/jib.ahead-of-print/jib-2017-0082/jib-2017-0082.xml`.

Giuseppe Paglia, Ólafur E. Sigurjónsson, Óttar Rolfsson, Soley Valgeirsdottir, Morten Bagge Hansen, Sigurdur Brynjólfsson, Sveinn Gudmundsson, and Bernhard O. Palsson. Comprehensive metabolomic study of platelets reveals the expression of discrete metabolic phenotypes during storage. *Transfusion*, 54:2911–2923, May 2014. doi:10.1111/trf.12710. URL `http://onlinelibrary.wiley.com/doi/10.1111/trf.12710/abstract`.

Nicolas Rodriguez, Alex Thomas, Leandro Watanabe, Ibrahim Y. Vazirabad, Victor Kofia, Harold F. Gómez, Florian Mittag, Jakob Matthes, Jan D. Rudolph, Finja Wrzodek, Eugen Netz, Alexander Diamantikos, Johannes Eichner, Roland Keller, Clemens Wrzodek, Sebastian Fröhlich, Nathan E. Lewis, Chris J. Myers, Nicolas Le Novère, Bernhard Ø. Palsson, Michael Hucka, and Andreas Dräger. JSBML 1.0: providing a smorgasbord of options to encode systems biology models. *Bioinformatics (Oxford, England)*, 31:3383–3386, October 2015. ISSN 1367-4811. doi:10.1093/bioinformatics/btv341. URL `http://bioinformatics.oxfordjournals.org/content/31/20/3383`.

Michael Römer, Johannes Eichner, Andreas Dräger, Clemens Wrzodek, Finja Wrzodek, and Andreas Zell. ZBIT Bioinformatics Toolbox: a Web-Platform for Systems Biology and Expression Data Analysis. *PLoS ONE*, 11(2):e0149263, February 2016. doi:10.1371/journal.pone.0149263. URL `http://dx.doi.org/10.1371%2Fjournal.pone.0149263`.

Felix Streichert and Holger Ulmer. JavaEvA : a Java based framework for Evolutionary Algorithms. Technical Report WSI-2005-06, Centre for Bioinformatics Tübingen, University of Tübingen, January 2005.

James T. Yurkovich, Benjamin J. Yurkovich, Andreas Dräger, Bernhard O. Palsson, and Zachary A. King. A padawan programmer's guide to developing software libraries. *Cell Systems*, 5(5):431–437, nov 2017a. doi:10.1016/j.cels.2017.08.003. URL `https://doi.org/10.1016/j.cels.2017.08.003`.

James T. Yurkovich, Daniel C. Zielinski, Lauence Yang, G. Paglia, O. Rolfsson, Ó. E. Sigurjónsson, J. T. Broddrick, A. Bordbar, K. Wichuk, S. Brynjólfsson, S. Palsson, S. Gudmundsson, and B. O. Palsson. Quantitative time-course metabolomics in human red blood cells reveal the temperature dependence of human metabolic networks. *Journal of Biological Chemistry*, 292:jbc.M117.804914, October 2017b. ISSN 0021-9258. doi:10.1074/jbc.M117.804914. URL `http://www.jbc.org/lookup/doi/10.1074/jbc.M117.804914`.
