# Supplementary material for: Visualizing metabolic network dynamics through time-series metabolomic data
Source: BMC Bioinformatics. 2020 Jul 7;21:130. doi: 10.1186/s12859-020-3415-z (PMC7119163; doi:10.1186/s12859-020-3415-z)
Supplement: Supplementary file 13 — Additional file 15 This document describes the features of the application SBMLsimulator and explains how to use them. [file 12859_2020_3415_MOESM15_ESM.pdf]

EBERHARD KARLS UNIVERSITÄT TÜBINGEN

MATHEMATISCH-NATURWISSENSCHAFTLICHE FAKULTÄT

**Users' Guide**

# SBMLsimulator

## A graphical user interface for efficiently simulating and visualizing computational models in biology

Andreas Dräger[1,2,3,*]

January 1, 2020

Institutional affiliations:

[1] Computational Systems Biology of Infection and Antimicrobial-Resistant Pathogens, Institute for Biomedical Informatics (IBMI), University of Tübingen, 72076 Tübingen, Germany

[2] Department of Computer Science, University of Tübingen, 72076 Tübingen, Germany

[3] German Center for Infection Research (DZIF), partner site Tübingen, Germany

*Corresponding author: draeger@informatik.uni-tuebingen.de

SBMLsimulator is a fast, accurate, and intuitive program for dynamic simulation and heuristic parameter optimization of models encoded in the Systems Biology Markup Language (SBML). To ensure the high reliability of this software, its internal simulation library had been benchmarked against the entire SBML Test Suite and all models from the BioModels Database database. SBMLsimulator includes the extensive collection of nature-inspired heuristic optimization procedures for efficient model calibration from the framework EvA2. SBMLsimulator provides an easily usable Graphical User Interface (GUI) and several command-line options to be suitable for large-scale batch processing and model calibration. SBMLsimulator runs on all platforms that provide a standard Java™ Virtual Machine (JVM) for a desktop environment. SBMLsimulator is an open-source program, which is based on the open-source libraries JSBML and Systems Biology Simulation Core Library (SBSCL), both of which can be obtained separately and used via their Application Programming Interface (API).

Version 2.0 of SBMLsimulator comes with a variety of additional features for the visualization of biological networks in the form of Systems Biology Graphical Notation (SBGN) Process Diagrams (PD) powered by the yFiles library for graph drawing. Upon loading SBML files, SBMLsimulator either draws the layout of the biological network as given in the file or (if the SBML file does not contain any graph layout information), it automatically generates SBGN-style diagrams. In either case, the network can be accessed in the graph view. Simulation results, as well as loaded experimental measurement data from Character-Separated Value (CSV) files, can be mapped to the networks to display the data in their full biological context. SBMLsimulator can generate animations for time-series data that can then be exported to various movie file formats or as snapshot images for selected time points.

For a more intuitive description of the features described in this users' guide, a series of video tutorials are available at /c/systemsbiology/. For many chapters in this tutorial, corresponding video tutorials are available online. You can find the direct links to those videos in the text.

This document is based on an earlier version of a users' guide to SBMLsimulator by Dörr, Keller, Zell, and Dräger (2014).

# Contents


**1 Introduction** **1**
1.1 Main program features . . . . . 1

**2 Installation** **3**
2.1 Requirements . . . . . 3
2.2 Starting the application . . . . . 4

**3 Loading model files and running a simulation** **5**
3.1 Obtaining an example model . . . . . 5
3.2 Loading, exploring, and simulating a kinetic model . . . . . 6
3.3 Dynamic simulation of a model . . . . . 8
3.4 Next steps . . . . . 12

**4 Parameter estimation** **14**
4.1 Preparing and loading measured data . . . . . 14
4.2 Defining a quality function . . . . . 16
4.3 Launching the heuristic optimization workbench EvA2 . . . . . 17
4.4 Save modifications of the model . . . . . 19

**5 Embedding a model layout in an SBML file and preparation of experimental data** **24**
5.1 Embedding a graph layout within a constraints-based model . . . . . 24
5.2 Preparation of experimental data . . . . . 28
5.3 Summary . . . . . 30

**6 Visualization of manually created layouts** **31**
6.1 Opening a constraint-based model with a graph layout . . . . . 31
6.2 Preparation of experimentally measured data . . . . . 32
6.3 Interactive visualization of data on a network map . . . . . 32
6.4 Summary . . . . . 33

**7 Command-line arguments and preferences** **35**
7.1 Simulator input/output options . . . . . 35

7.2 Simulation options . . . . . . . . . . . . . . . . . . . . . . . . . . . 35
7.3 Estimation Options . . . . . . . . . . . . . . . . . . . . . . . . . . . 37
7.4 Options for the graphical user interface . . . . . . . . . . . . . . . . . . . . 39
7.5 Plot Options . . . . . . . . . . . . . . . . . . . . . . . . . . . . . 39
7.6 CSV options . . . . . . . . . . . . . . . . . . . . . . . . . . . . . 40
7.7 Garuda options . . . . . . . . . . . . . . . . . . . . . . . . . . . . 40

**8 License** **41**
8.1 Included third-party libraries and packages . . . . . . . . . . . . . . . . . . 41

**9 FAQ / Troubleshooting** **42**

**A Acknowledgments** **44**
A.1 Alumni . . . . . . . . . . . . . . . . . . . . . . . . . . . . . . . 44
A.2 Collaborators . . . . . . . . . . . . . . . . . . . . . . . . . . . . . 44
A.3 Contributors . . . . . . . . . . . . . . . . . . . . . . . . . . . . . 44
A.4 Special thanks . . . . . . . . . . . . . . . . . . . . . . . . . . . . 45
A.5 Funding . . . . . . . . . . . . . . . . . . . . . . . . . . . . . . . 45

**B Release notes** **46**
B.1 Version 1.0 . . . . . . . . . . . . . . . . . . . . . . . . . . . . . 46
B.2 Version 1.1 . . . . . . . . . . . . . . . . . . . . . . . . . . . . . 46
B.3 Version 1.2 . . . . . . . . . . . . . . . . . . . . . . . . . . . . . 46
B.4 Version 1.2.1 . . . . . . . . . . . . . . . . . . . . . . . . . . . . 47
B.5 Version 2.0 . . . . . . . . . . . . . . . . . . . . . . . . . . . . . 47

**C Acronyms** **49**

**Bibliography** **51**

**Index** **56**

# Figures

3.1 Loading of a model . . . . . . . . . . . . . . . . . . . . . . . . . . 6
3.2 SBMLsimulator with a loaded model . . . . . . . . . . . . . . . . . . . . 7
3.3 Example of an automatically generated layout of a metabolic network model . . . . 8
3.4 Display of model tree . . . . . . . . . . . . . . . . . . . . . . . . . 9
3.5 Selection of quantities for plotting . . . . . . . . . . . . . . . . . . . . 10
3.6 Starting a simulation in SBMLsimulator . . . . . . . . . . . . . . . . . . 11
3.7 Simulation results plotted in the right panel of the window . . . . . . . . . . . 13
3.8 Table with simulation data . . . . . . . . . . . . . . . . . . . . . . . 13

4.1 Example of the data import dialog . . . 15
4.2 Expanded “CSV Options” allow giving details for the input file . . . 16
4.3 SBMLsimulator after simulation and loading of measured data . . . 17
4.4 Selection of quantities to estimate . . . 20
4.5 EvA2 settings window . . . 21
4.6 EvA2 calibrating a model . . . 22
4.7 Configuring the optimization procedure . . . 23
4.8 Intermediate estimation result . . . 23

6.1 Warning when loading a model with undefined initial values . . . 31
6.2 Graph animation . . . 34
6.3 Animated plot . . . 34

# Tables

3.1 Available ODE solver implementations . . . 11
3.1 Available Ordinary Differential Equation (ODE) solver implementations (continued) 12

# Listings

2.1 Launching SBMLsimulator from the command-line with additional memory . . . 4

4.1 Input file example for experimental data . . . 15
4.2 Input file example for parameter estimation file . . . 19

5.1 Extract from the converted SBML layout file for iMM904 . . . 25
5.2 Example script for merging an SBML layout into a model . . . 26
5.3 First reaction from the merged SBML file with a layout for iMM904 . . . 29
5.4 Part of the example yeast data set in CSV format . . . 29

6.1 Definition of a moving camera animation for a *Ken Burns effect* . . . 32

# 1 Introduction

SBMLsimulator (Dörr et al., 2014) is an easily usable, portable, and powerful simulation, visualization, and parameter optimization engine for biochemical network models in SBML format (Hucka et al., 2003).

SBML is a file format that stores systems biology models in a structured way. The main idea behind SBML is that a model should always yield identical results, no matter which software was used to build the model. SBMLsimulator got its name from this file format.

SBMLsimulator uses the Systems Biology Simulation Core Library (SBSCL) (Keller et al., 2013), which is based on JSBML (Dräger et al., 2011; Rodriguez et al., 2015), as its computational core and combines its functions with EvA2 (Kronfeld et al., 2010), a Java™ framework for nature-inspired heuristic optimization procedures. In this context, optimization means the estimation of model parameters, i.e., the calibration of a model to given experimentally measured data (Dräger et al., 2009).

As the name suggests, SBMLsimulator was initially developed to provide an intuitive graphical user interface for loading SBML models, running simulations, and plotting the results. With version 2.0, its capabilities greatly expanded: SBMLsimulator comes with a comprehensive visualization implementation. This feature makes it possible to dynamically visualize experimental and simulated data in the context of biological networks in the form of SBGN process diagrams (Rougny et al., 2019).

Just like SBML that has been developed for encoding models of biological systems in a structured way, SBGN brought essential design principles to the models. The aim of SBGN is that biological networks can be drawn in a standardized fashion, thereby removing any potential ambiguity.

This document is intended to guide you through the function and use of the SBMLsimulator. We will start by explaining how to install and launch the program and how to use its more advanced features.

Now, we briefly discuss the main features of the SBMLsimulator before the next chapter gets us started by installing the program on your computer.

## 1.1 Main program features

SBMLsimulator has been designed to

- ✔ parse SBML files and display its content in a GUI for exploration.
- ✔ insert missing values into the model that are required to perform a dynamic simulation.

- ✔ interpret metabolical, gene regulatory, and signal transduction models in terms of an Ordinary Differential Equation (ODE) systems and solve those with numerical integration methods.
- ✔ fit the model to given experimental data and save the optimization results in the model.
- ✔ plot experimental and simulation data, i.e., temporal changes of all model components.
- ✔ export simulation results as CSV or image files.
- ✔ save user preferences and restore all your settings when launched next time.
- ✔ be launched as a Garuda gadget from the Garuda dashboard (Ghosh et al., 2011).
- ✔ be used in a command-line mode without GUI.
- ✔ be embedded as an API in a third-party program, allowing access to all of its functions in more complex scripts and procedures.
- ✔ draw network layouts based on the information provided in loaded SBML files or to automatically generate SBGN process diagrams in cases where the SBML files do not contain any layout information.
- ✔ to map data that may originate from biological experiments or from computer simulation onto SBGN network diagrams
- ✔ to allow users zooming and panning while dynamic animations are proceeding.
- ✔ to generate snapshot images or animated videos from the data mapping, including implementation of a *Ken Burns effect* (sliding viewport window).

# 2 Installation

To obtain a copy of the SBMLsimulator, we open a web browser and navigate to the Github page of the Dräger research group at /draeger-lab/ and go to SBMLsimulator. When we click on "releases," we can find the latest release on top, currently SBMLsimulator 2.0.

☞ Watch this example online at /Eu4uSPmNXVI.

SBMLsimulator comes in two different versions: As a stand-alone Java™ Archive (JAR) file or as a gadget for users of the Garuda platform that is available from garuda-alliance.org. Once we embed the correct version of the SBMLsimulator in our Garuda installation folder, we can launch the application from the Garuda dashboard or from any other Garuda-enabled software.

Let us download the JAR file and launch the application with a double click within our downloads folder. To run SBMLsimulator, we need to have the Java™ Virtual Machine (JVM) installed on our computer, which Oracle provides for personal or commercial use[1], or its open-source pendant[2] for all other purposes.

## 2.1 Requirements

### 2.1.1 Software

SBMLsimulator is entirely written in Java™ and runs on any desktop operating system where a suitable Java™ Virtual Machine (JVM) is installed (Java™ Development Kit (JDK) version 8 or newer), including Microsoft Windows, Linux, and macOS. Version 2.0 of SBMLsimulator has been tested on Microsoft Windows 10, OpenSuSE Tumbleweed (release 20191228), and macOS versions 10.14.6 (Mojave) and 10.15.2 (Catalina). See, for example, the Java™ SE download page[2]. If you encounter any issues with your operating system, chapter 9 might provide answers to you. To report problems or bugs, it is recommended to use the project's issue tracker at /draeger-lab/SBMLsimulator/issues/.

### 2.1.2 Hardware

With at least 1 GB main memory, we should be able to perform most tasks without any problem. SBMLsimulator runs without an active internet connection.

[1] https://www.java.com
[2] https://openjdk.java.net

## 2.2 Starting the application

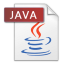

If we downloaded a ZIP-file, we need to unzip it before starting the application. In the most simple case, we can launch SBMLsimulator by double-clicking at the Java™ application icon (see image next to this text). SBMLsimulator can run out-of-the-box on all systems where a Java™ Virtual Machine (JVM) is installed and does not require any further installations. When launching the application, it opens with a splash screen indicating the version number.

We can also start the application on all desktop operating system by typing the following on the command prompt:

```
java -jar -Xms128m -Xmx1024m SBMLsimulator_v<version.number>.jar
```

Please note that we might have to change <version.number> to the actual name of the JAR file we downloaded, e.g., SBMLsimulator_v2.0.jar. In this example, we pass arguments to the Java™ Virtual Machine (JVM) to make a minimum of 128 MB and a maximum of 1024 MB of memory available to the program. In most cases, SBMLsimulator needs more than 128 MB of memory, so it might be convenient to create a shortcut and start the application with as much memory as available. If we have 2 GB of Random-Access Memory (RAM), for example, we might want to start the application with the command shown in listing 2.1. This small example already indicates that launching the program from the command-line has some advantages compared to double-clicking the icon. On the command-line, we can pass so-called “command-line arguments” not only to the Java™ Virtual Machine (JVM) but also directly to the program. These allow us, for instance, to directly open a specific model file when launching the application or customize many other settings. SBMLsimulator shows us an overview of all available command-line options when adding -? to the start command (see chapter 7 for details).

To simplify matters, we could create a start-scripts to run the application with as much memory as possible. How much memory we actually need strongly depends on the size of our input datasets.

SBMLsimulator is a bi-lingual program, which has been translated to German in addition to its English user interface. When launching the application, it uses English, unless our operating system has a German environment. We can select the language of the program by passing the parameter -Duser.language=en to the Java™ Virtual Machine (JVM) upon the start of the program if we like to have an English user interface or de for the German interface.

Now that we got SBMLsimulator installed and running on our computer, we will explore its use in the following chapters.

**Listing 2.1** | Launching SBMLsimulator from the command-line with additional memory

```
java -Xms128m -Xmx1400M -jar SBMLsimulator_v<version.number>.jar
```

# 3 Loading model files and running a simulation

In this chapter, we will discuss a selection of features of the software SBMLsimulator. First, we will start by explaining where to find systems biology models, how to load them into the application, and how to run a simulation.

☞ Watch this example online at YouTube/CVzp_XtIaHU.

We will then discuss a more advanced feature, namely the calibration of model parameters to experimental data.

## 3.1 Obtaining an example model

Several online repositories exist that house systems biology models. Let us explore how we can get the dynamic model of human hepatocytes that Bucher et al. published in 2011 in *BMC Systems Biology* from an online database in SBML format. We open a web browser and enter `biomodels.net` in the address bar, which redirects us to BioModels Database (Malik-Sheriff et al., 2019) hosted at the European Bioinformatics Institute, or EBI.

Since we are interested in a specific model, we can simply enter some keywords in the search field, such as "Bucher hepatocytes," and click at the magnifying glass. And here is the result with the model identifier № 328. The tab "Files" provides several downloads to us, of which the model file in SBML format is most interesting to us for now, which is listed at the top. Note that SBML files can have various file extensions. Most common are `.sbml` or, as it is here, `.xml`. Just one click, and we should have the model in our 🗀 `Downloads` folder.

Many other published models available from the BioModels Database website[1] have been curated by a team of experts. If we like to download a model, click the respective Identifier (Id), which brings us to a page containing a description of the model and a button "Download SBML." We position the cursor at this button, and then we see the possible SBML levels and versions to download. If we click on the respective combination of level and version, the desired SBML file will be downloaded.

Alternatively, we can also create an SBML model ourselves. To this end, dedicated software solutions, such as CellDesigner (Funahashi et al., 2008), can be used. For downloading CellDesigner, please go to the project's website[2].

[1] `http://www.ebi.ac.uk/biomodels-main/publmodels`
[2] `http://www.celldesigner.org`

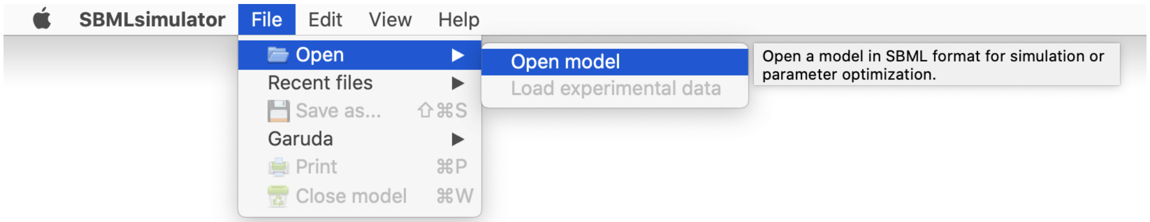


**Figure 3.1** | Loading of a model. One possibility to open a model is to select File › Open › Open model. Depending on our operating system, we can also use one of the following keystroke combinations ⌘ + O (macOS) or Ctrl + O (Linux and Microsoft Windows). To open one of the ten recently opened models, we can select File › Recent files.

## 3.2 Loading, exploring, and simulating a kinetic model

To perform model simulations, we first need to load an SBML model. If we additionally want to compare the simulation results to experimental data or run a parameter estimation, it is required to also load the experimental data into SBMLsimulator. In our example, we generate simulated data from the example model. We can obtain simulated data, too, by running a simulation with SBMLsimulator and exporting the numerical solution to a CSV file. This procedure is explained in detail below.

### 3.2.1 Open a model file

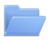

We can simply drag & drop the model file (an SBML document) into the application or select File › Open › Open model to load a model (see fig. 3.1). Another option would be to click at the folder icon in the toolbar, which is displayed next to this text. Depending on our operating system, we can also use the keystroke combinations ⌘ + O (macOS) or Ctrl + O (Linux and Microsoft Windows). SBMLsimulator memorizes up to ten previously opened SBML documents in the menu under File › Recent files, where the most recently used file will appear at the topmost position. To open one of the ten previously loaded models, we can also use the keystroke combinations ⌥ or Alt + 0 to 9 (operating system-dependent).

### 3.2.2 Graphically exploring a model

Now, the model is loaded, and the window's appearance changes as fig. 3.2 shows:

- ➢ The right part of the window is for plotting simulation results and experimental data points (if experimental data has been loaded). In the bottom part of the window, we can determine simulation preferences like the solver or the number of simulation steps and the quality function for comparing experimental data (if present) to simulation results.
- ➢ In the center left part of the window the compartment values, the initial values of the reactive species, and parameter values in the model can be changed. Please note that after typing a

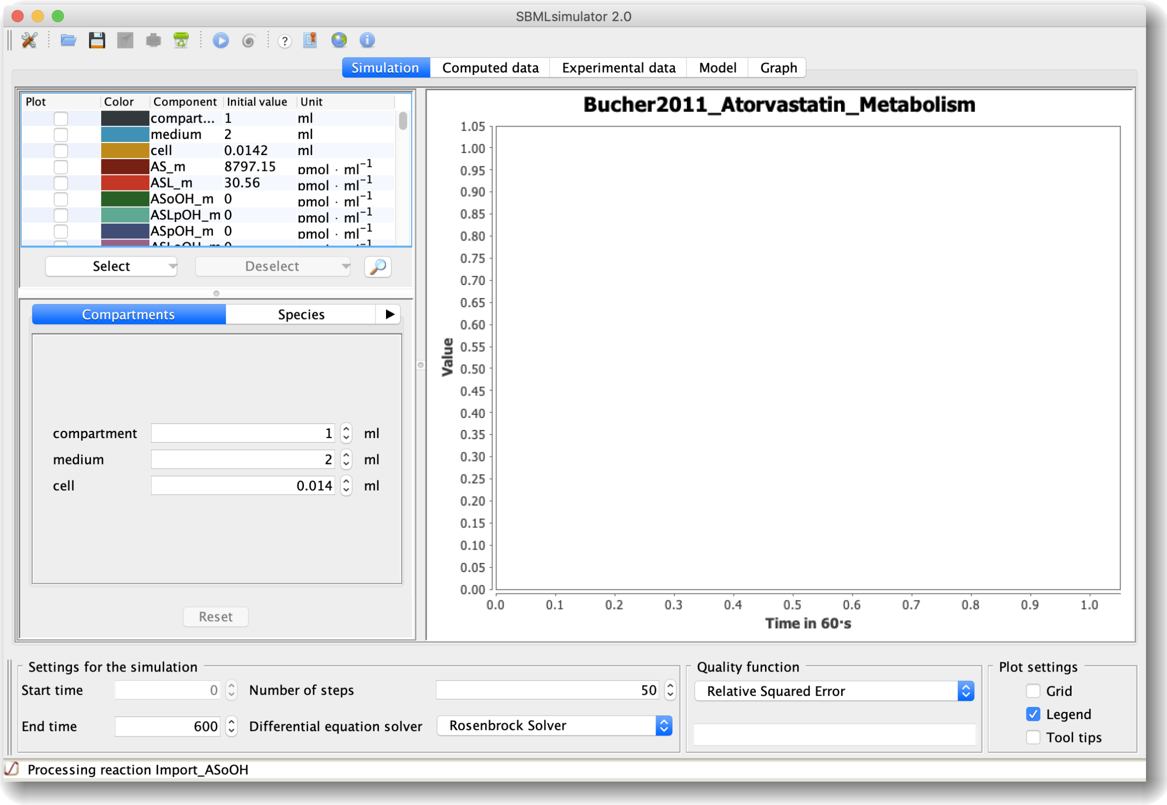


**Figure 3.2** | SBMLsimulator with a loaded model. In this window, we can choose the simulation settings (bottom), the parameter values (center left), and the quantities to plot (upper left). The plot section is on the right.

new value in, the change only takes effect after we either press the return key [⏎] or the tabulator key [⇥].

➢ The upper left part is for choosing the quantities that should appear in the plot.

The user interface of the SBMLsimulator comprises several tabs. In the Model tab, we can explore the structure of the model in a tree view, including all of its components, such as compartments, species, parameters, or reactions. Section 3.2.2 discusses the content of this tab in detail. The Graph tab visually displays the model, which may look like fig. 3.3. In case of the example model by Bucher et al., the authors did not embed any layout information into their model, and SBML-simulator has hence to automatically create a display following the SBGN guidelines. Chapter 6 discusses this feature in more detail.

We can explore the tree structure of the model if we choose the tab Model, where we see the SBML document in a tree structure that we can examine (fig. 3.4). A search function at the bottom allows us to browse the model for any of its components. Here we can search for

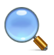

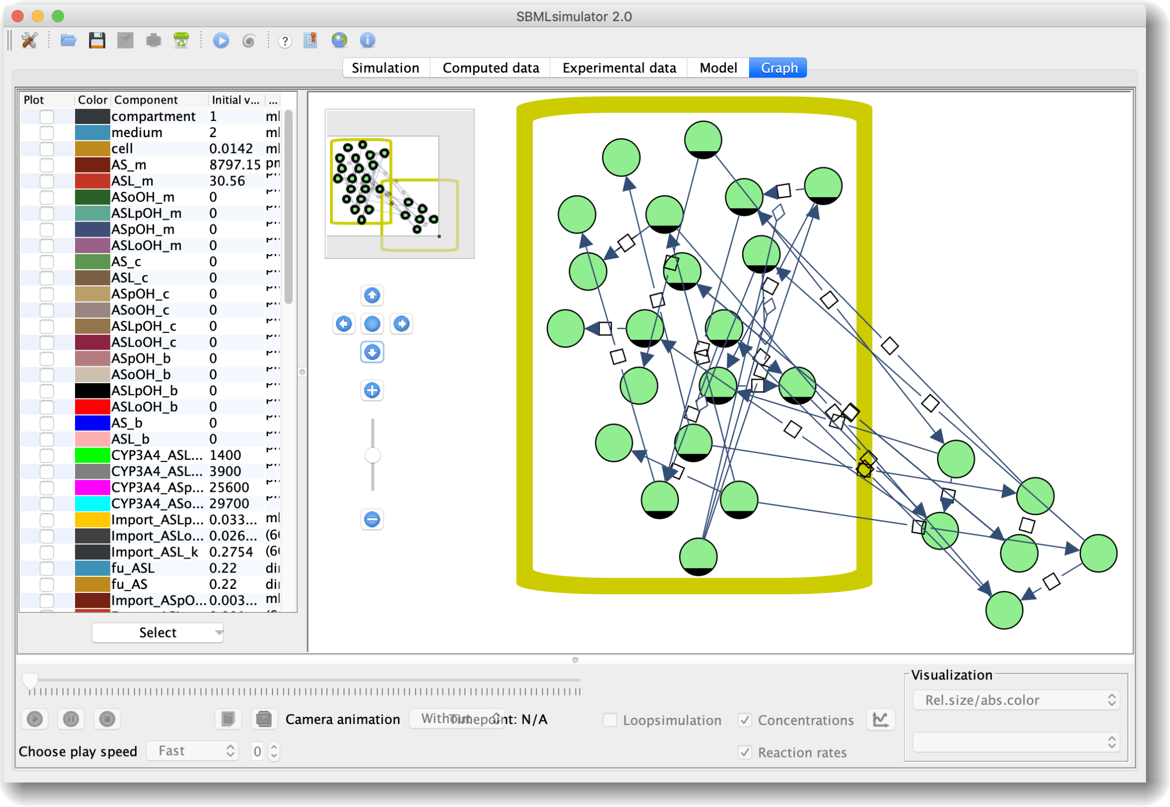


**Figure 3.3** | Example of an automatically generated layout of a metabolic network model. When loading an SBML file without embedded layout information, SBMLsimulator tries to automatically generate a graph display in the style of an SBGN process diagram. This example shows one such layout generated from the model by Bucher et al.

Ids or names of components, for instance, all Michaelis constants ($K_M$ values) in the model. The tree will be expanded, and elements that do not match our filter criteria will be removed from the view. When clicking at individual model parts, the program displays detailed information about the selected element.

## 3.3 Dynamic simulation of a model

Next, let us run a dynamic simulation, i.e., interpret the model in terms of an Ordinary Differential Equation (ODE) system. At the bottom is a control panel where we can adjust how the simulation should be performed, for instance, with 300 steps. We can also select the solver that calculates the result for us. SBMLsimulator offers a large variety of integration routines for this purpose. The default solver, "Rosenbrock," is the most precise method and solves the broadest

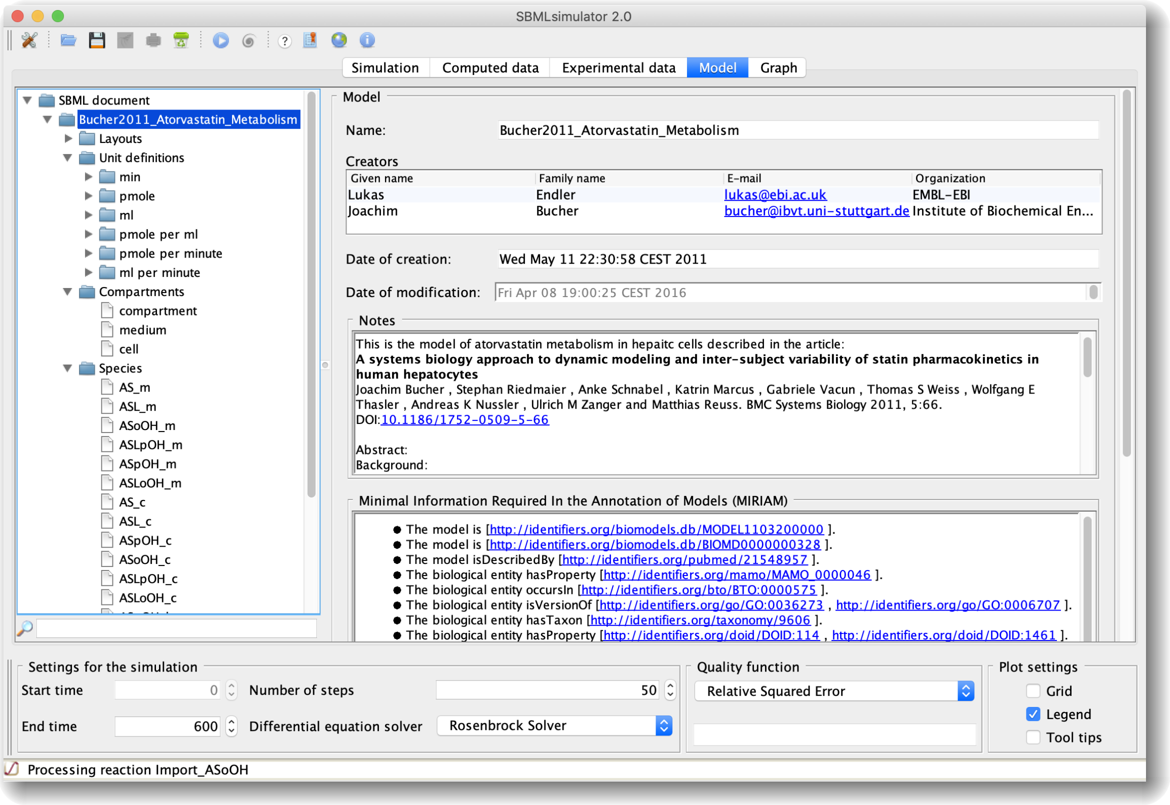


**Figure 3.4** | Display of model tree. The model tree is shown when clicking on the Model tab. We can explore the model by clicking at elements within the tree.

range of models. However, its high preciseness comes at the cost of a generally higher run time compared to the other solvers. Table 3.1 lists all solvers that SBMLsimulator provides together with a short description and a reference for more information. When clicking the run button at the top, as fig. 3.6 shows, the calculation starts.

Once the computation has finished, we can see all selected model components in the plot area. On the left panel in the Simulation tab, we can select the model components whose simulation results should be added to the plot diagram. To this end, we click at the individual checkboxes for model components of interest. The buttons Select and Deselect give us a choice to visualize or remove an entire group of elements from the plot, such as all species or all fluxes. In fig. 3.5, for instance, we select to plot all species values. Figure 3.7 shows us the result of this model simulation. Selecting model components is necessary because SBMLsimulator does not know which model components we like to display in the plot area.

When finishing a simulation run, the tab Computed data becomes active, where we can see the simulated time course from the plot in tabular format.

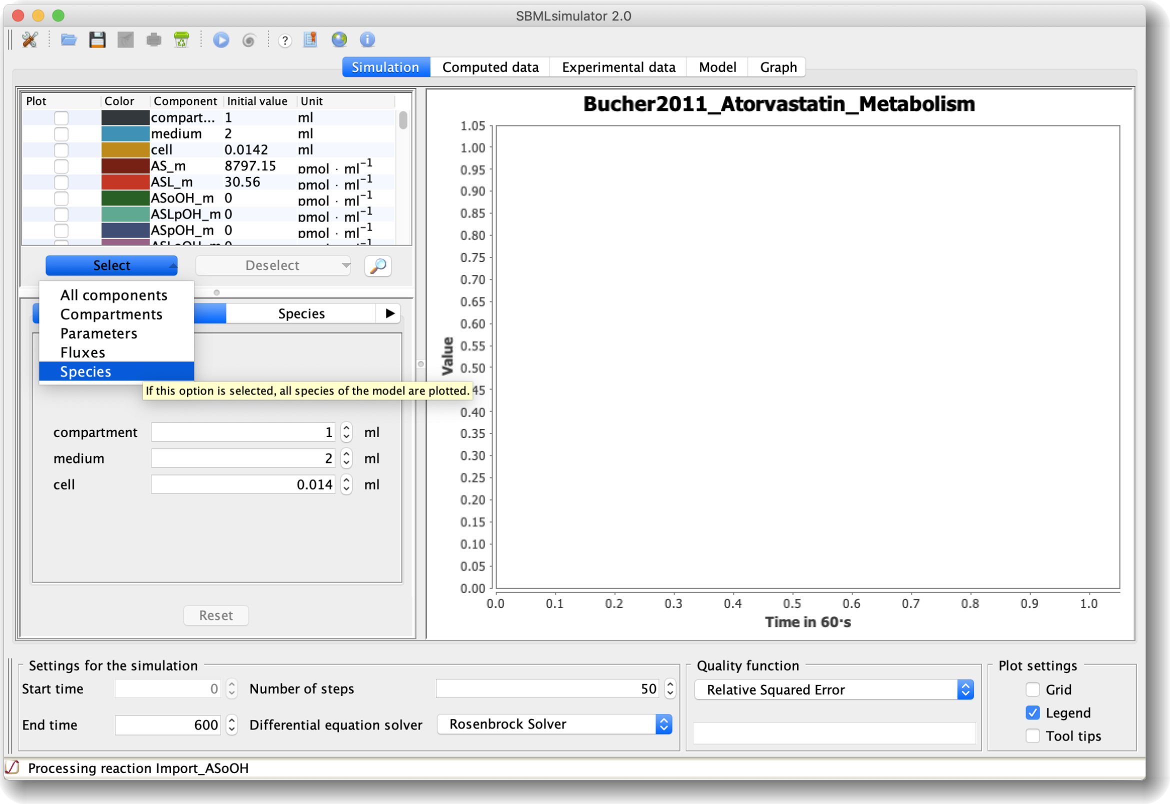


**Figure 3.5** | Selection of quantities for plotting. We can, for instance, click on Select › Species to choose all species for the diagram.

With the availability of this data set, the graph view in the Graph has now also updated and automatically maps the simulated data onto the generated network. We can play these data as a movie, zoom in, and pan the system around while it is running. The controls at the bottom adjust the properties of this animation. Chapter 6 gives more details of this feature.

The view in Simulation also allows us to play with the initial values or parameters in the model in the panel to the left. For instance, we might be interested in setting a new value for some Michaelis parameter ($K_M$ value), and see how the results of the simulation change after rerunning the model. If we have a time-course data set from laboratory experiments available, we can load it into SBMLsimulator and apply a plethora of heuristic optimization methods to automatically estimate optimal values of such model parameters. Chapter 4 explains this is a topic in detail.

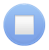

If in some cases, a simulation run consumes too much time, we can always interrupt it by clicking at the stop button in the toolbar (see image at the side of this text), which is activated whenever we launch a simulation.

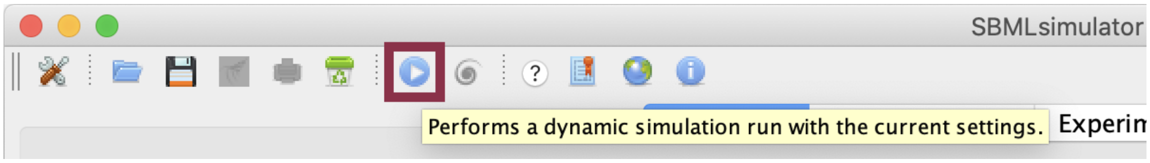


**Figure 3.6** | Starting a simulation in SBMLsimulator. By clicking on the simulation button, the simulation of the model is started.

**Table 3.1** | Available Ordinary Differential Equation (ODE) solver implementations

| Solver | Description |
|---|---|
| Adams-Bashforth Apache Software Foundation (2013) | This is an explicit multi-step integration method. The solver uses a step-size adaptation. It is faster than Rosenbrock, but not applicable to very stiff differential equation systems. This solver is provided by Apache Commons. |
| Adams-Moulton (Apache Software Foundation, 2013) | The method by Adams and Moulton is an implicit multi-step integration method, which uses a step-size adaptation. It is also faster than Rosenbrock, but not applicable to all models. This solver is provided by Apache Commons. |
| Dormand-Prince 54 (Apache Software Foundation, 2013) | The Dormand Prince in an explicit integration method of order 5, which belongs to the family of Runge-Kutta methods. Step-size adaptation is included in the routine. This solver works for most models but can have problems with extremely stiff differential equation systems. It is provided by Apache Commons. |
| Dormand-Prince 853 (Apache Software Foundation, 2013) | This solver is similar to Dormand-Prince 54, but it is of order 8. It comprises more function evaluations than Dormand-Prince 54 and is, therefore, more precise, but also slower. It is provided by Apache Commons. |
| Euler (Press et al., 1992) | The explicit Euler method. This is a very fast and straightforward solver, that lacks a step-size adaptation and might, therefore, be imprecise. |
| Gragg-Bulirsch-Stoer (Apache Software Foundation, 2013) | This method belongs to the most efficient and accurate methods with step-size adaptation for non-stiff differential equations. Its use is not recommended for stiff differential equations. This solver is provided by Apache Commons. |
| Higham-Hall 54 (Apache Software Foundation, 2013) | The method by Higham and Hall is a Runge-Kutta method of order 5 with step-size control. The solver is similar to the Dormand-Prince 54 solver. It is provided by Apache Commons. |

Continued on the next page. . .

**Table 3.1** | Available Ordinary Differential Equation (ODE) solver implementations (continued)

| Solver | Description |
|---|---|
| Rosenbrock (Press et al., 1992) | This adapted solver comprises Rosenbrock's method, which includes an adaptation of step-size. This implementation is specifically dedicated to the precise simulation of SBML models and is applicable for very stiff differential equation systems. But it might be significantly slower for some models than the other solvers. |
| Runge-Kutta (Press et al., 1992) | The classical fourth-order Runge-Kutta method. It is fast, but imprecise for many models, as it does not contain a step-size adaptation. |

### 3.3.1 Save simulation data

Saving information from the GUI is context-sensitive in SBMLsimulator: Depending on which tab is selected, we can save different pieces of information. If we want to save computed data from a simulation, we need to select the tab Computed data first (see fig. 3.8). Then we can save the simulation data under File › Save as. Just like for the plot data, we can also use keystroke combinations to save our simulation data. To this end, we just select the tab Computed data and hit the keys ⌘+⇧+S if we are working under macOS, or Ctrl+⇧+S for Linux and Microsoft Windows.

### 3.3.2 Save plot image

If we like to save the current plot as an image, just select the tab Simulation. We can then save the image of the results plot by clicking at File › Save as. For users who prefer keystroke combinations, it is also possible to save the plot by hitting the keys ⌘+⇧+S if we are working under macOS, or Ctrl+⇧+S for Linux and Microsoft Windows. By clicking at the printer icon (displayed next to this text), we can print the current plot or save it as a Portable Document Format (PDF). The print function can also be used with the keystroke combination ⌘+P (macOS) or Ctrl+P (Microsoft Windows and Linux).

## 3.4 Next steps

We have now walked through how to load a model and run a simulation using SBMLsimulator. In Chapter 4, we will explore how to automatically estimate parameter values. In other words, this will explain how a model can be calibrated to experimentally obtained time-course data. After discussing how to embed manually created network layouts in SBML files in Chapter 5, chapter 6 will then focus on how to create dynamic videos of metabolic networks that you can use for visual analytics or embed within scientific presentations.

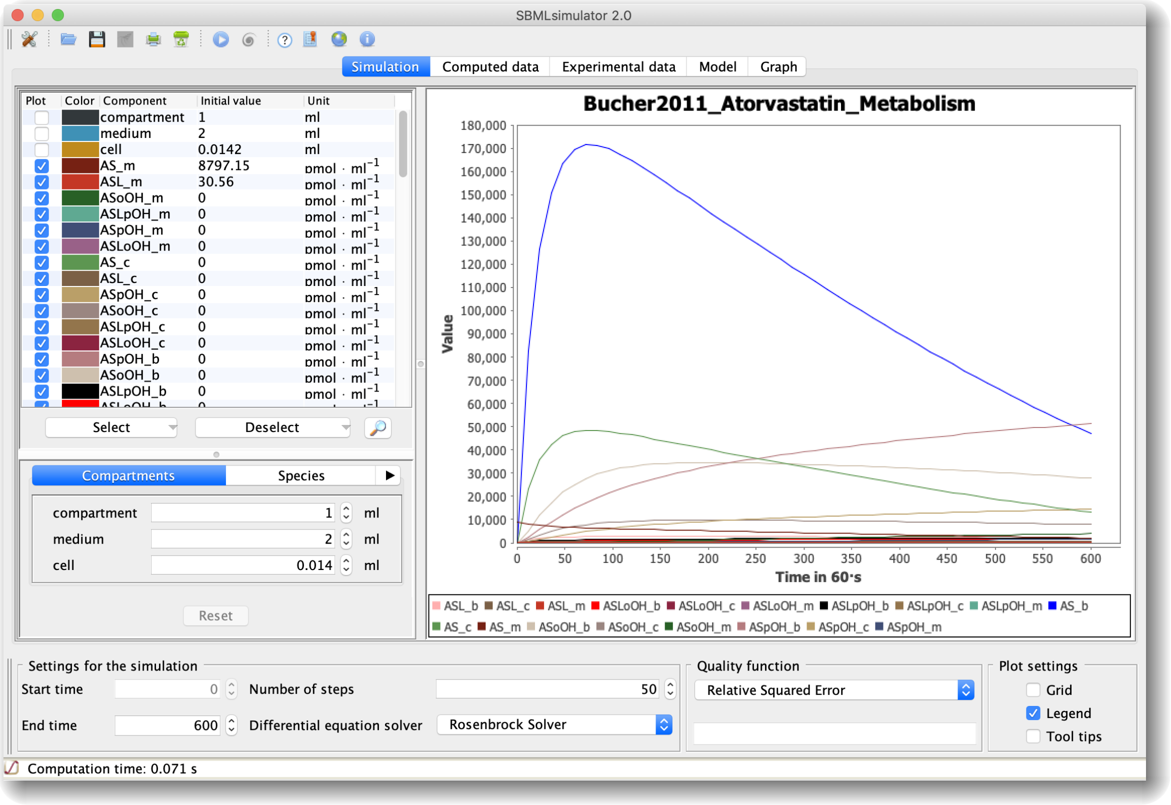


**Figure 3.7** | Simulation results plotted in the right panel of the window

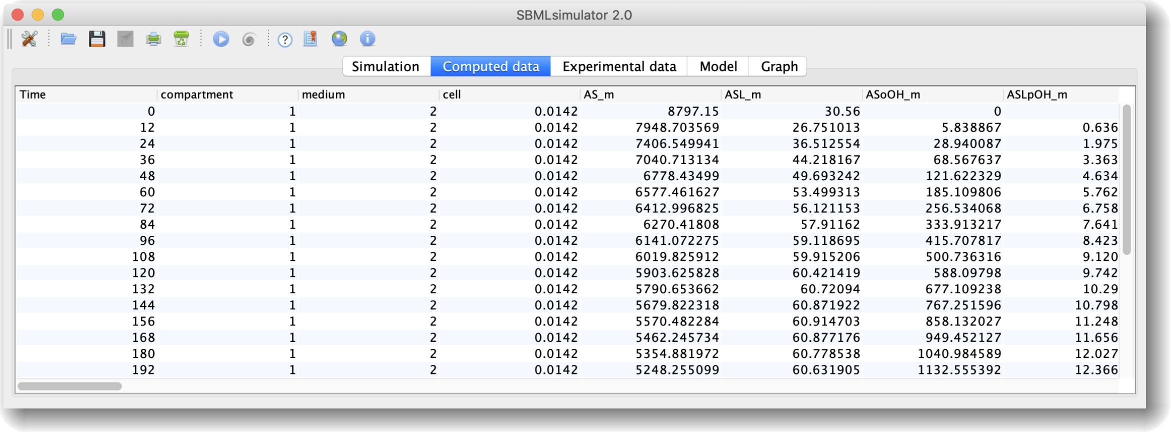

| Time | compartment | medium | cell | AS_m | ASL_m | ASoOH_m | ASLpOH_m |
|---|---|---|---|---|---|---|---|
| 0 | 1 | 2 | 0.0142 | 8797.15 | 30.56 | 0 | |
| 12 | 1 | 2 | 0.0142 | 7948.703569 | 26.751013 | 5.838867 | 0.636 |
| 24 | 1 | 2 | 0.0142 | 7406.549941 | 36.512554 | 28.940087 | 1.975 |
| 36 | 1 | 2 | 0.0142 | 7040.713134 | 44.218167 | 68.567637 | 3.363 |
| 48 | 1 | 2 | 0.0142 | 6778.43499 | 49.693242 | 121.622329 | 4.634 |
| 60 | 1 | 2 | 0.0142 | 6577.461627 | 53.499313 | 185.109806 | 5.762 |
| 72 | 1 | 2 | 0.0142 | 6412.996825 | 56.121153 | 256.534068 | 6.758 |
| 84 | 1 | 2 | 0.0142 | 6270.41808 | 57.91162 | 333.913217 | 7.641 |
| 96 | 1 | 2 | 0.0142 | 6141.072275 | 59.118695 | 415.707817 | 8.423 |
| 108 | 1 | 2 | 0.0142 | 6019.825912 | 59.915206 | 500.736316 | 9.120 |
| 120 | 1 | 2 | 0.0142 | 5903.625828 | 60.421419 | 588.09798 | 9.742 |
| 132 | 1 | 2 | 0.0142 | 5790.653662 | 60.72094 | 677.109238 | 10.29 |
| 144 | 1 | 2 | 0.0142 | 5679.822318 | 60.871922 | 767.251596 | 10.798 |
| 156 | 1 | 2 | 0.0142 | 5570.482284 | 60.914703 | 858.132027 | 11.248 |
| 168 | 1 | 2 | 0.0142 | 5462.245734 | 60.877176 | 949.452127 | 11.656 |
| 180 | 1 | 2 | 0.0142 | 5354.881972 | 60.778538 | 1040.984589 | 12.027 |
| 192 | 1 | 2 | 0.0142 | 5248.255099 | 60.631905 | 1132.555392 | 12.366 |

**Figure 3.8** | Table with simulation data. Under the tab Computed data, we can see the simulation results in a table. They can be saved under File ⟩ Save as, by clicking the floppy icon in the toolbar, or by using an operating system-dependent keystroke combination.

# 4 Parameter estimation

In many cases, biological models contain quantities with uncertain values. These values must be estimated by calibrating the model to given experimental data. To facilitate this complicated procedure, SBMLsimulator comes with the optimization toolbox EvA2 that provides an extensive collection of nature-inspired heuristic optimization procedures, e.g., Evolution Strategies (ES) (Rechenberg, 1973; Schwefel, 1975), Genetic Algorithms (GA) (Holland, 1975), Differential Evolution (DE) (Storn, 1996), or Particle Swarm Optimization (PSO) (Clerc, 2006a; Clerc and Kennedy, 2002) and Tribes (Clerc, 2006b), as well as niche-based methods that can be be particularly useful for applications in systems biology (Kronfeld et al., 2009).

## 4.1 Preparing and loading measured data

First of all, we will need to load a time-course data set into the SBMLsimulator that we can use for calibrating our model of interest. The idea is that for every measured quantity from the data set, the distance of the simulation results to the measured values should be minimized. To load the data into SBMLsimulator, we need to make sure that our experimentally measured data set conforms to the required file format. The application reads CSV files, which are mostly tab- or comma-separated files of data.

### 4.1.1 Exporting CSV files from spreadsheet software

Modern spreadsheet software, such as Microsoft Excel, Google Sheets, LibreOffice Calc, Apple Numbers, etc., supports exporting data to CSV. We are here exemplarily walking through the process for the most widely used software, Microsoft Excel. In other tools, the procedure follows similar steps.

To use data from Microsoft Excel, we can simply open our Microsoft Excel spreadsheet, click File › Save as and select "Tab-separated text file." For all files, the application requires one column called `time` to be present. This column should contain for each row the time point it refers to. Note that the headline of the file must be indicated with a leading pound symbol (`#`). The other columns should be called with the Id of the respective quantity in the model and should contain the measured values at the time points, or Not a Number (NaN) if the measurement for a time point is missing.

Hence, we need a column with the time points and multiple columns that state the values of each quantity at each time point. The corresponding SBML file defines the units of these quantities so

**Listing 4.1** | Input file example for experimental data

```
#time    s1           s2           s3
0        32.456E-12   7.02         12E32
4        5E-54        179.05E-14   0.005
...      ...          ...          ...
```

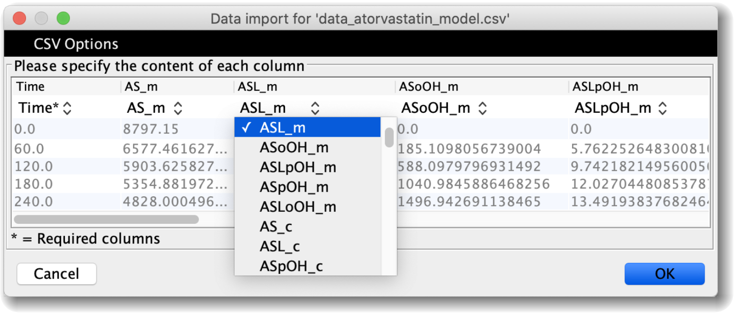


**Figure 4.1** | Example of the data import dialog. The "CSV Options" panel can be expanded to correct auto-detected input file properties. Where needed, the table at the bottom of the import dialog allows us to specify the matching of each column from the data file to a corresponding model component.

that we do not have to make units explicit in our CSV file. The listing 4.1 displays an example of how an input file may look like.

### 4.1.2 Importing the measured data from a file

In general, all data must be processed time-course data in tabular text files (see section 4.1.1 for descriptions and examples of the input file formats). Just like in the case of model files, we now have several different ways how to open one or more of these datasets in SBMLsimulator: We can

- drag & drop the data file(s) into the application.
- select File » Open » Load experimental data to open one or more multiple files.
- use the keystroke combination ⌘ + O or Ctrl + O, depending on our operating system.

Please note that we cannot open experimental data before loading a model. This is because an SBML file can contain various meta-data about model components (such as units), whereas the simple CSV format might not suffice to build the complex data structure for the display. If we open multiple data files, median values will be used for parameter estimation in each time point.

Figure 4.1 shows an example of the file input dialog. Here, we open the simulation data saved in section 4.4. The displayed data set has been restricted to the quantities that Bucher et al. experimentally measured for their parameter estimation. SBMLsimulator tries to automatically determine which column from our data file belongs to which model component based on the Ids. Only in case of Id mismatches, we may need to specify the content of each column in the table at the bottom of the import dialog. In these cases, we have to click on the combo box below the captions.

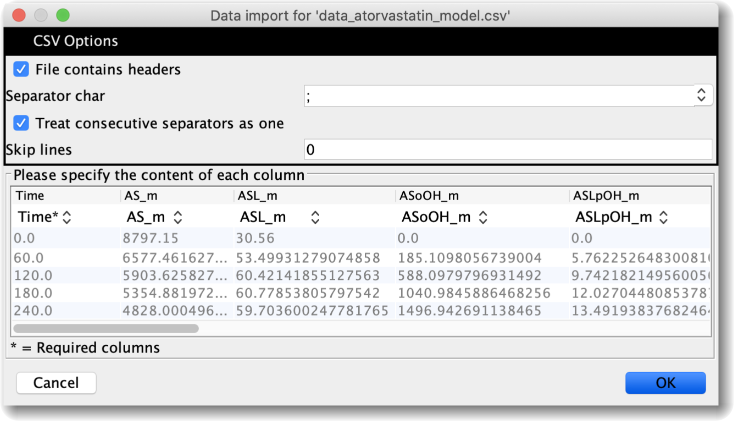


**Figure 4.2** | Expanded "CSV Options" allow giving details for the input file. These properties are auto-detected and only need to be changed if the auto-detection failed to correctly infer those properties.

SBMLsimulator also tries to automatically infer the format of the data file, e.g., what character is used to separate the columns, the character encoding of the file, etc. If the input file format cannot be automatically inferred (which is not the case in our example here), we may click on the black "CSV Options" label to specify further options (like "column separator char" or, if the file contains headers, see fig. 4.2). After the experimental data are imported, the plot shows the data points as dots (see fig. 4.3), or in the form of box plots, in the case, we opened more than one data file (not shown).

## 4.2 Defining a quality function

Before we can calibrate our model to our time-course data set(s), we need to briefly discuss what we consider an optimal solution. The main idea is that when simulating a calibrated model, we should obtain curves that are as close as possible to experimentally observed values. In other words, the distance between the calculated behavior of every model component and the corresponding data should be minimal. For instance, if we have measurement data available for the overall intracellular citric acid concentration, the model should yield very similar curves for this reactive species as well.

Model calibration now means that we have to choose values for parameters or initial values for unknown model components such that this difference becomes minimal. Minimal here means that we have to minimize a distance function. SBMLsimulator brings with it a selection of distance functions to choose from, which are in analogy to biological evolution also called *fitness* functions: (i) (Relative) Euclidean distance, (ii) (Relative) Manhattan distance, (iii) (Relative) N-Metric, (iv) Pearson Correlation, and (v) Relative Squared Error (RSE). For first attempts or without prior knowledge about the structure of the solution space, it is recommendable to use either the RSE. The RSE has the advantage that higher concentrated substances in the cell do not dominate the quality of the solution because this formula weights the contributions of all model components based on their abundance. Care must be taken if some model component is known to take a value of precisely zero at any time because, in this case, the RSE would not be defined. To circumvent

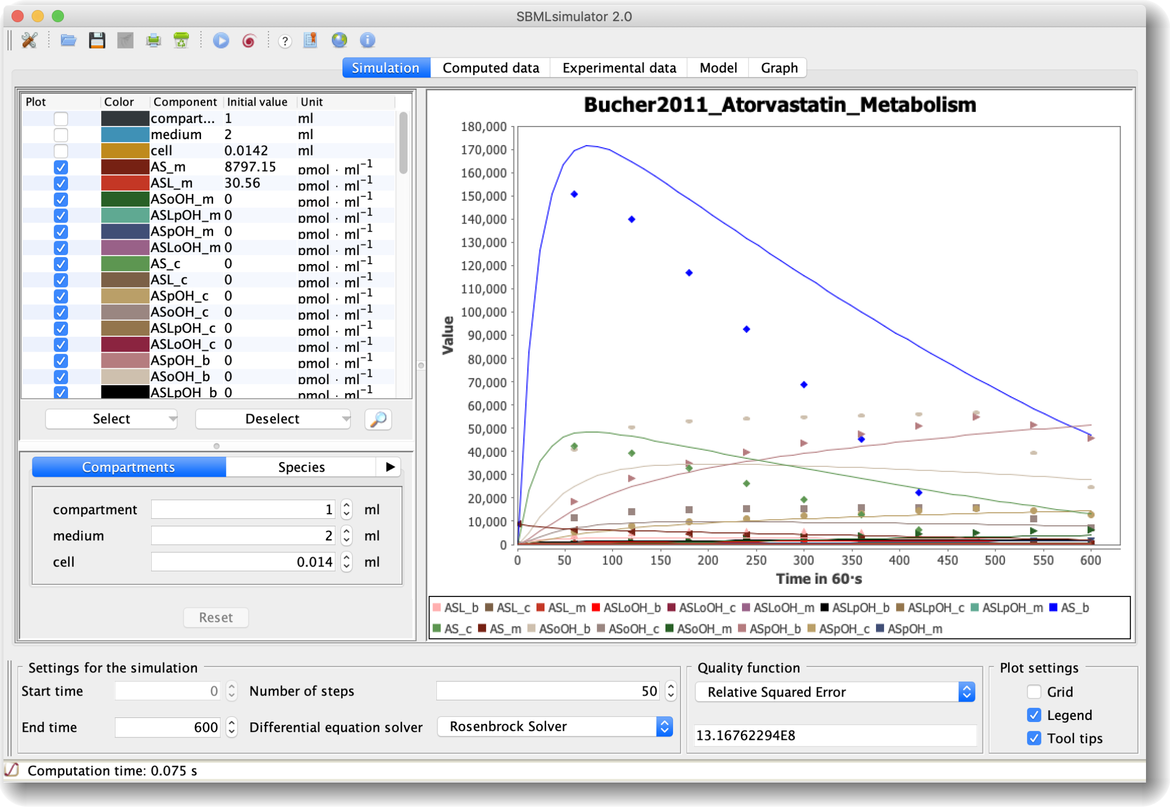


**Figure 4.3** | SBMLsimulator after simulation and loading of measured data. The plot shows the simulation results and the experimentally measured data points as dots.

this problem, SBMLsimulator provides a sufficiently high default value, which we can adjust to our needs within the Preferences menu.

The panel at the bottom in the Simulation tab contains a drop-down menu where we can select an appropriate fitness function. The RSE is selected by default.

## 4.3 Launching the heuristic optimization workbench EvA2

Now that we have loaded a model and corresponding time-course data into SBMLsimulator, the estimation button becomes active in the toolbar (see the icon next to this text) and also does the corresponding entry in the menu: Edit > Optimization.

After clicking either a window like the one shown in see fig. 4.4 appears, in which we can select which quantities from the model to estimate. Since computational models may contain large numbers of potential optimization targets, the dialog presents them in the four tabs Compartments, Species, Global Parameters, and Local Parameters. Each of these tabs has two buttons at the bottom

that allow us to select all quantities within this tab or to deselect them all at once. If we like to choose just a few variables as estimation targets, a search function at the bottom allows us to narrow down the content of the table. For every estimation target, we can also define their allowable ranges. Note that we here distinguish between a range for initialization and boundaries for the entire search space. This distinction can help to generate stable initial solutions and to find allowable values more efficiently.

Typically, model calibration has to be repeatedly performed. Selecting optimization targets manually each time can be quite cumbersome. For this reason, SBMLsimulator allows us to save a selection to a configuration file in CSV format and to also read in such a file within this dialog. To load a configuration from such a file, we click the Open button. We can also save our configuration by clicking the Save button.

Listing 4.2 shows an example of such a configuration file. It is also possible to write such a file from scratch (or even algorithmically) and hence to skip the entire process of selecting any optimization targets from the dialog window. The configuration file has to be in CSV format with precisely the following five columns:

[`Id`] the unique identifier of the model component whose (initial) value is uncertain and needs to be estimated.

[`initialMinimum`] the minimum allowable value for the initialization of (more or less) random values for the quantity in this row. This means that when the optimization algorithm makes its first guess about a possible value for this quantity, the value in this column must not be surpassed.

[`initialMaximum`] the maximal allowable value for the initialization of (more or less) random values for the quantity in this row. This means that when the optimization algorithm makes its first guess about a possible value for this quantity, the value in this column must not be exceeded.

[`minimum`] The value in this column must not be surpassed at any time during the optimization.

[`maximum`] This is the absolute maximum value that this variable can possibly take on.

It is recommended to pick a relatively narrow initialization range (possibly around plausible comparable values from other studies or from online databases) and a broad full range. As soon as we are satisfied with our selected quantities, we click the OK button.

Now, EvA2 starts in a separate window as fig. 4.5 shows. There we have the option to select the define the termination criterion for the optimization (“terminator”) and the estimation method (“optimizer”), each of which may have specific settings. Figure 4.7 shows the configuration of differential evolution (Storn, 1996) as an example. For first attempts, the default settings should already yield results of sufficient quality. In a large-scale comparative study, Dräger et al. (2009) investigated how to best tweak the settings of specific optimization methods. Their publication

**Listing 4.2** | Input file example for parameter estimation file

```
[Id]               [initialMinimum]   [initialMaximum]   [minimum]   [maximum]
Import_ASLpOH_k    1E-7               0.1                1E-7        0.1
Import_ASLoOH_k    1E-7               0.1                1E-7        0.1
Import_ASL_k       1E-7               1                  1E-7        1
...                ...                ...                ...         ...
```

contains a table with recommended settings that we can apply to the optimization methods available in EvA2.

When we click the Start button, the estimation begins. Please note:

- If there are initial values (i.e., values at time point 0) for some model quantities given in the data, SBMLsimulator takes these values as initial values for the simulations necessary during the estimation.
- In the case of multiple experimental datasets loaded, the medians of the initial values are taken.
- For the quantities without initial values given in the data, the initial values defined in the model are chosen.

While the model calibration is running, EvA2 continuously plots the current best solution for the quality function we chose. It also outputs the current best set of estimated values (fig. 4.6). In each iteration, the main window of SBMLsimulator updates the plot and draws the simulation result for the current best solution as fig. 4.8 shows.

## 4.4 Save modifications of the model

To save a model, for instance, after manually changing the values of some model variables, such as parameters, species, or compartment, or after calibration with EvA2, we first have to select the tab Model. Then we can save the simulation data under File › Save as. This function will save the model in its current state to a file of choice, including all of our modifications, such as the newly estimated values of all uncertain model components. We can either save the model to a new file or overwrite an existing one. Again, also for this purpose, we can use keystroke the combinations ⌘+⇧+S (for macOS) or Ctrl+⇧+S (for Linux and Microsoft Windows) when the Model tab is selected. At any time, the program also accepts the operating system-dependent keystroke ⌘+S or Ctrl+S to overwrite the loaded model with the current modifications. The same effect can be obtained by choosing File › Save from the menubar—we just need to make sure that the tab Model is selected.

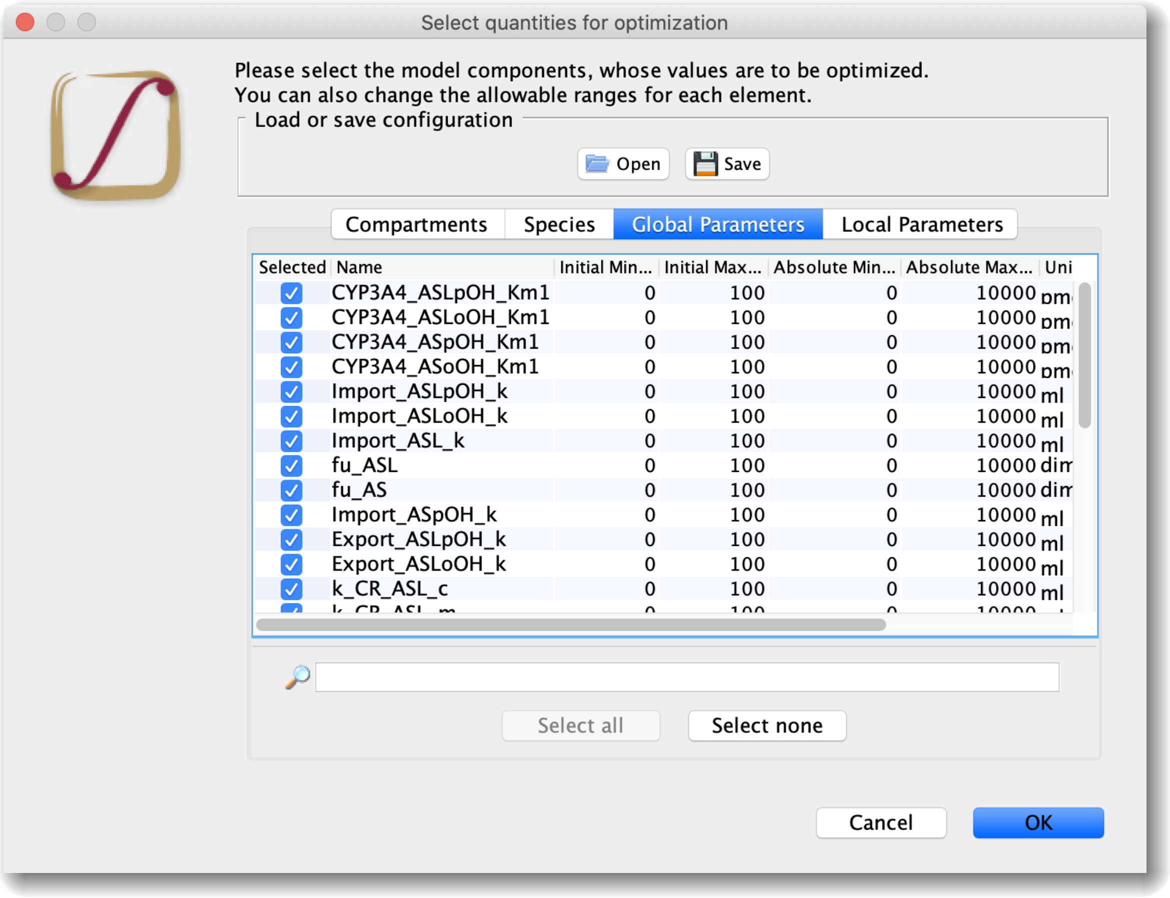


**Figure 4.4** | Selection of quantities to estimate. We can manually select the variables to be determined together with their ranges by modifying the displayed tables. Alternatively, we may open a CSV file by clicking on the Open button. The current configuration of quantities and their ranges (for initialization as well as for the entire estimation process) can be saved by clicking the Save button. The search function at the bottom helps us to navigate through the names or identifiers that are potential optimization targets. When we type a name in the text field, the above table will be reduced to elements that contain this name while typing. The buttons Select all and Select none are also helpful to choose all or none of the items within one tab as optimization targets. Note that each tab has these buttons separately, so our choice in one tab does not affect any other tabs. By default, SBMLsimulator assumes that all global and local parameters in the model are potential optimization targets.

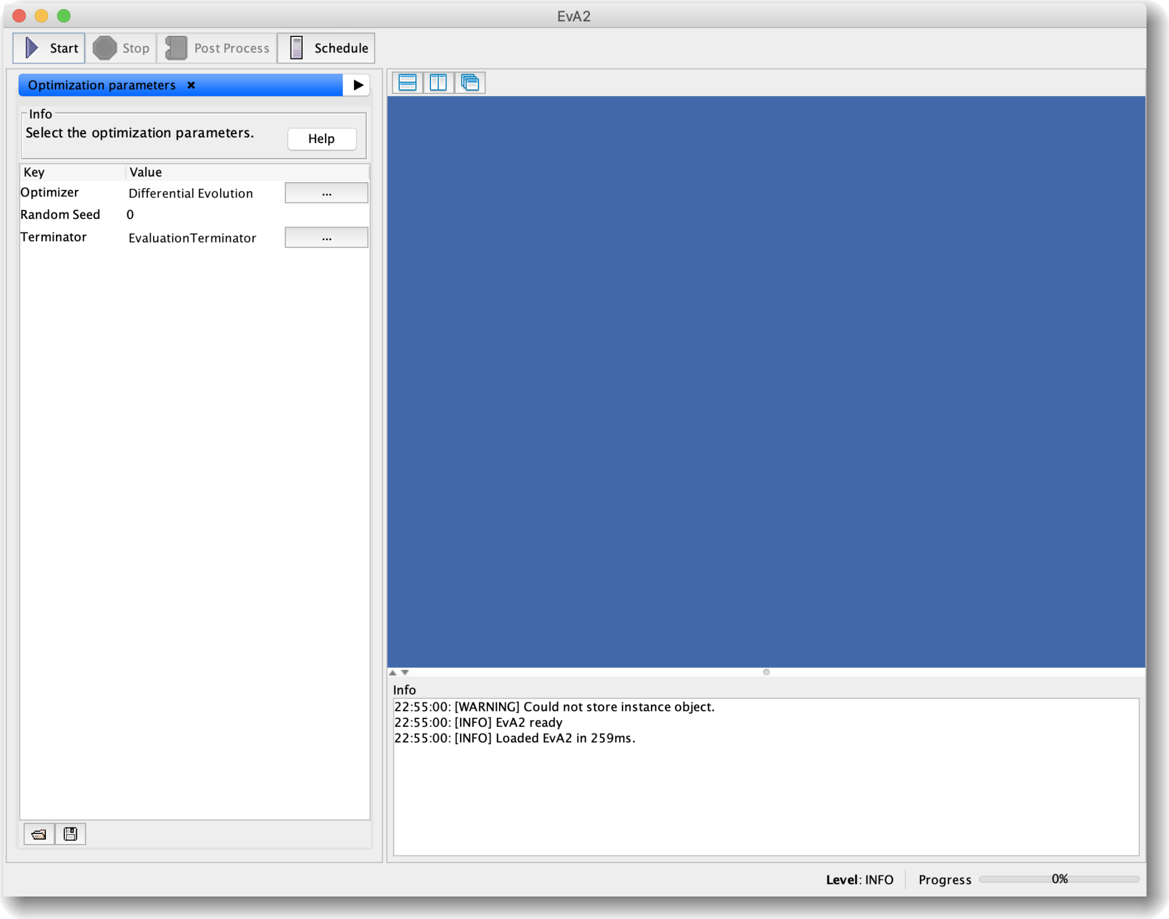


**Figure 4.5** | EvA2 settings window. EvA2 lets us choose the parameter estimation method and the specific settings: We just click at the text field next to the label "optimizer." A separate dialog window will appear that offers a large number of optimization procedures (fig. 4.7 shows this dialog for the example of differential evolution). In the same way, an appropriate termination criterion can also be set by clicking at the text field that is labeled "terminator." After clicking the Start button, the estimation begins. During the optimization, a window will appear that displays the quality improvement of the solution, the so-called *fitness*. This is the distance between our experimental data and the simulation result for the currently best parameter set that we defined in section 4.2. If the optimization is successful, the fitness will decrease as time proceeds. We can interrupt the optimization at any time by hitting the Stop button. When our selected termination criterion is reached, the optimization will stop, and we can continue working with our optimized model in the main window of SBMLsimulator.

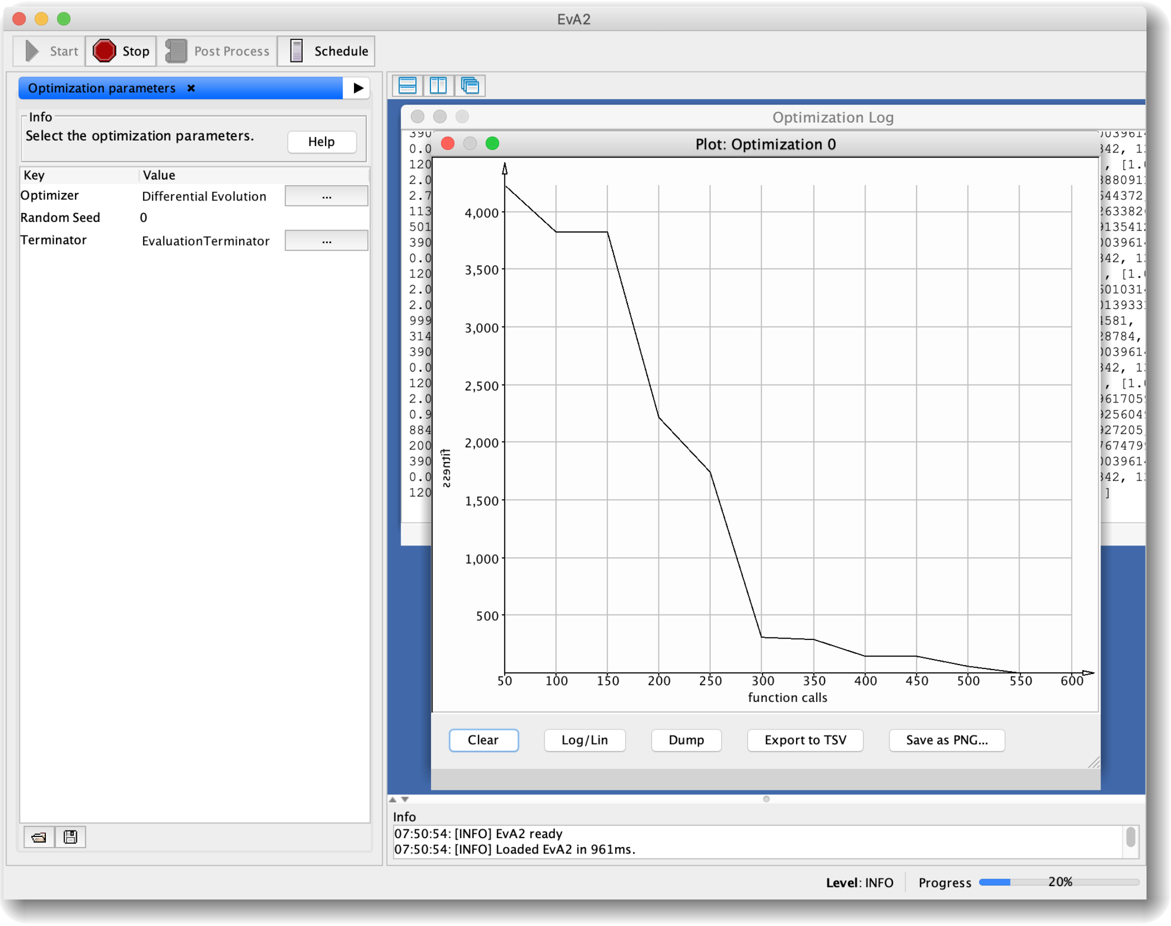


**Figure 4.6** | EvA2 calibrating a model. During the calibration, EvA2 displays the current best fitness in an interior window as a function of the number of model evaluations. Another interior window displays details about the current set of possible solutions for the optimization problem. Whenever EvA2 finds a new optimum, it forwards this solution to the Simulation tab of SBMLsimulator, which then plots the new solution as depicted in fig. 4.8.

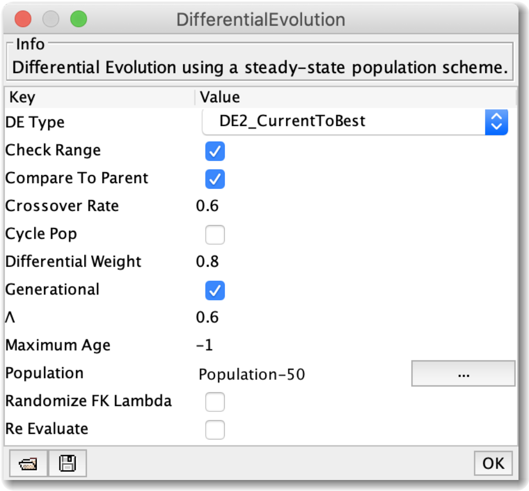


**Figure 4.7** | Configuring the optimization procedure. We can select an optimization procedure and define its specific settings in this window. Apart from DE (Storn, 1996), EvA2 provides many other nature-inspired heuristic optimization routines, such as Particle Swarm Optimization (PSO) (Clerc, 2006a; Clerc and Kennedy, 2002). The settings of the chosen method can be changed by clicking in the text field right of it and typing the new value or by clicking in the checkbox, respectively.

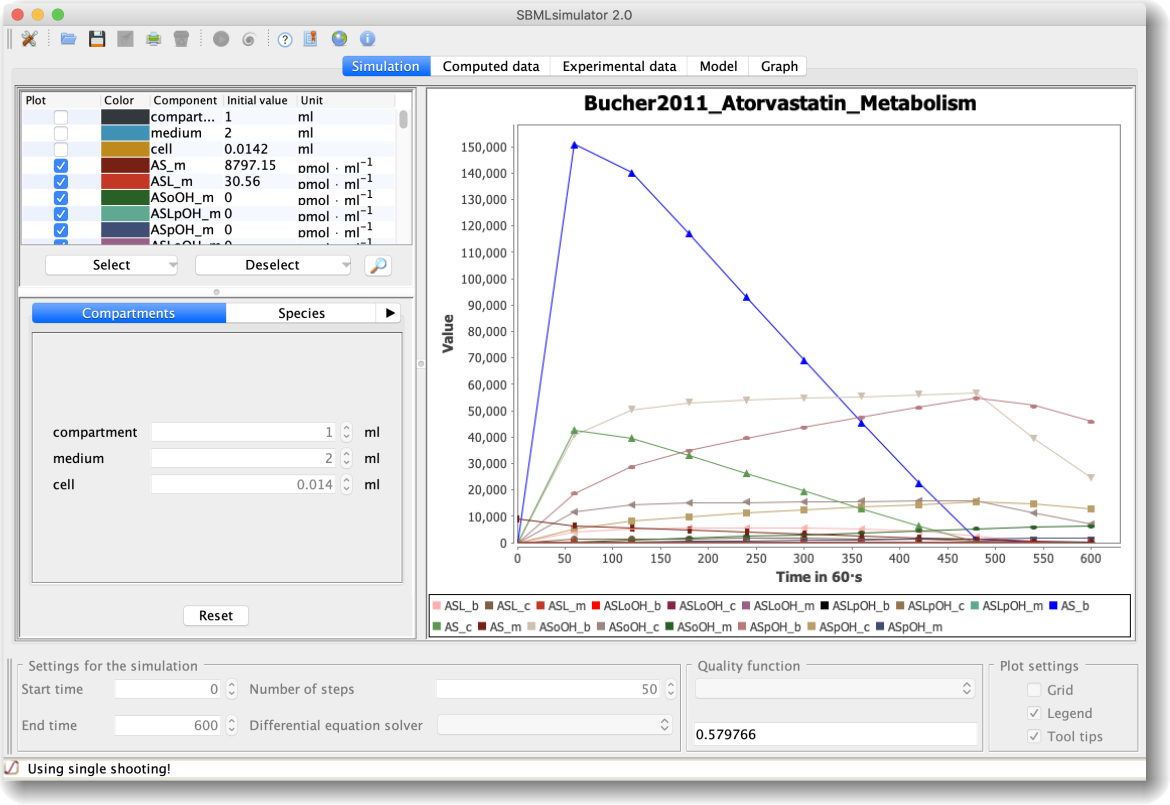


**Figure 4.8** | Intermediate estimation result. After each generation of solutions by EvA2, the simulation result with the current best parameter estimation is plotted. The current value of the quality function is also shown.

# 5 Embedding a model layout in an SBML file and preparation of experimental data

Until now, we explored how simulated data is calculated from a dynamic model, how to estimate uncertain values of variables within the model, and briefly mentioned that simulated data can be mapped to a graphical display of the model, which SBMLsimulator automatically generated for us. Next, we will explore how to embed a manually drawn map within an SBML file. We will then discuss how to prepare a published time-course data set for visual analysis with SBMLsimulator.

☞ Watch this example online at /Coe0h2sFFSQ.

## 5.1 Embedding a graph layout within a constraints-based model

The BiGG Models Database provides genome-scale metabolic models for various organisms. Let us search for a Genome-scale Metabolic Model (GEM) of yeast, or *Saccharomyces cerevisiae*, among them. From the two yeast models currently in BiGG Models Database, we pick iMM904 that Mo et al. created in 2009 for a publication in *BMC Systems Biology* and download the model in compressed SBML format. At the bottom of the page, we can find a map of the central carbon metabolism. Let us download that map JavaScript Object Notation (JSON) format. We can convert this map to SBML with layout information using the software EscherConverter (King et al., 2015a) that is available at /draeger-lab/EscherConverter. To this end, we download the executable JAR file of EscherConverter, run it with a double click, drag the JavaScript Object Notation (JSON) file into EscherConverter and click the disk symbol to safe it in SBML format. The documentation of EscherConverter[1] has more details about this conversion procedure.

The SBML file we obtain from EscherConverter, however, only contains those parts of the model with relevance for the graphical display (listing 5.1). Information not relevant for the depiction of the map is lacking, such as further reactions and metabolites from iMM904 that are not shown in the network diagram as well as any Minimal Information Required In the Annotation of Models (MIRIAM) annotations (Juty et al., 2012) from the GEM. The reason is that the original Escher map does also not provide the full model.

We hence need to merge the core model we downloaded from BiGG Models Database with this SBML layout model (Gauges et al., 2015). To this end, we need to update all references from the layout components to the so-called SBML core components. For small models, we could do this manually, but for a model of this size, it can be conducive to write a script that does it for us.

[1] https://draeger-lab.github.io/EscherConverter/

**Listing 5.1** | Extract from the converted SBML layout file for iMM904

```
<?xml version='1.0' encoding='utf-8' standalone='no'?>
<!-- Created by EscherConverter version 1.2.0 on 2019-08-09 at 14:15:08
    MESZ with JSBML version 1.4. -->
<sbml xmlns="http://www.sbml.org/sbml/level3/version1/core"
    layout:required="false" level="3" version="1"
    xmlns:layout="http://www.sbml.org/sbml/level3/version1/layout/version1">
  <model id="_78086bfdab8ac8a8150cf4cd5dada037" name="iMM904.Central␣
      carbon␣metabolism">
      ...
      <layout:textGlyph layout:id="tg_269" layout:text="TCA␣Cycle">
        <layout:boundingBox>
          <layout:position layout:x="1763.683654492188"
              layout:y="5946.125330570312" layout:z="0"/>
          <layout:dimensions layout:depth="1" layout:height="50"
              layout:width="160"/>
        </layout:boundingBox>
      </layout:textGlyph>
    </layout:listOfTextGlyphs>
  </layout:layout>
</layout:listOfLayouts>
...
  <species boundaryCondition="false" compartment="m" constant="false"
      hasOnlySubstanceUnits="true" id="ac_m" name="Acetate"
      sboTerm="SBO:0000247"/>
  <species boundaryCondition="false" compartment="c" constant="false"
      hasOnlySubstanceUnits="true" id="ac_c" name="Acetate"
      sboTerm="SBO:0000247"/>
  <species boundaryCondition="false" compartment="c" constant="false"
      hasOnlySubstanceUnits="true" id="g6p_c" name="D-Glucose␣6-phosphate"
      sboTerm="SBO:0000247"/>
  <species boundaryCondition="false" compartment="m" constant="false"
      hasOnlySubstanceUnits="true" id="fadh2_m" name="Flavin␣adenine␣
      dinucleotide␣reduced" sboTerm="SBO:0000247"/>
</listOfSpecies>
<listOfReactions>
  <reaction compartment="e" fast="false" id="ACALDt" name="Acetaldehyde␣
      reversible␣transport" reversible="true" sboTerm="SBO:0000375">
    <listOfReactants>
      <speciesReference constant="true" id="ACALDt_reactant_1"
          sboTerm="SBO:0000010" species="acald_e" stoichiometry="1"/>
    </listOfReactants>
    <listOfProducts>
      <speciesReference constant="true" id="ACALDt_product_1"
          species="acald_c" stoichiometry="1"/>
    </listOfProducts>
  </reaction>
  <reaction compartment="m" fast="false" id="SUCD1m" name="Succinate␣
      dehydrogenase" reversible="true" sboTerm="SBO:0000375">
    <listOfReactants>
      ...
```

Listing 5.2 shows an example of such a script using JSBML (Rodriguez et al., 2015). The result is an SBML file of the full model with links to layout information.

**Listing 5.2** | Example script for merging an SBML layout into a model

```
/**
 * This script takes care of differences in the ID naming conventions
 * between Escher and BiGG by adding prefixes ({@code M_},{@code R_}, or
 * {@code G_}) where needed and also correcting other crossreferences.
 *
 * @param args
 *  This method requires as input the paths to three files:
 *  1) The result from a conversion Escher JSON to SBML
 *  2) The current implementation requires as 2nd argument a file
 *     containing already the layout pasted into an annotated model that
 *     previously did not have a layout. In a future version, it would be
 *     better to take the layout from the Escher conversion and to create
 *     a clone that is then added to the next file.
 *  3) The output file where to write the merged SBML document.
 * @throws IOException
 * @throws XMLStreamException
 */
public static void main(String[] args) throws XMLStreamException,
    IOException {
  SBMLDocument layoutDoc = SBMLReader.read(new File(args[0]));
  SBMLDocument doc = SBMLReader.read(new File(args[1]));
  Model m = doc.getModel();
  Layout l = ((LayoutModelPlugin)
      m.getPlugin("layout")).getListOfLayouts().get(0);
  int count = 0;
  for (SpeciesGlyph sg : l.getListOfSpeciesGlyphs()) {
    String ref = sg.getSpecies();
    char pref = 'M';
    if (!ref.startsWith(pref + "_")) {
      sg.setSpecies(createNewReference(ref, pref));
      System.out.println(++count + ".\t" + ref + "␣->␣" +
          sg.getSpecies());
    }
  }
  count = 0;
  for (TextGlyph tg : l.getListOfTextGlyphs()) {
    if (tg.isSetOriginOfText()) {
      if (tg.getOriginOfTextInstance() == null) {
        String ref = tg.getOriginOfText();
        char prefixes[] = {'M', 'R', 'G'};
        for (char pref : prefixes) {
          NamedSBase sbase = m.findNamedSBase(createNewReference(ref,
              pref));
          if (sbase != null) {
            tg.setOriginOfText(sbase.getId());
            System.out.println(++count + ".\t" + ref + "␣->␣" +
```

```
                  sbase.getId());
              break;
            }
          }
        }
      }
    }
    count = 0;
    for (ReactionGlyph rg : l.getListOfReactionGlyphs()) {
      if (rg.isSetReaction() && (rg.getReactionInstance() == null)) {
        String ref = rg.getReaction();
        NamedSBase sbase = m.findNamedSBase(createNewReference(ref, 'R'));
        if (sbase != null) {
          rg.setReaction(sbase.getId());
          System.out.println(++count + ".\t" + ref + "␣->␣" +
              sbase.getId());
        }
      }
      if (rg.isSetListOfSpeciesReferenceGlyphs()) {
        for (SpeciesReferenceGlyph srg :
            rg.getListOfSpeciesReferenceGlyphs()) {
          if (srg.isSetSpeciesReference()) {
            String ref = srg.getSpeciesReference();
            NamedSBase sbase = m.findNamedSBase(ref);
            if (sbase == null) {
              Reaction r = (Reaction) rg.getReactionInstance();
              NamedSBase other = layoutDoc.getModel().findNamedSBase(ref);
              if ((other != null) && (other instanceof SpeciesReference)) {
                SpeciesReference sr = (SpeciesReference) other;
                if (sr.isSetSpecies()) {
                  if (ref.contains("_reactant_")) {
                    setId(r.getListOfReactants(), sr.getSpecies(), ref);
                  } else if (ref.contains("_product_")) {
                    setId(r.getListOfProducts(), sr.getSpecies(), ref);
                  }
                }
              }
            }
          }
        }
      }
    }
    System.out.println(args[1]);
    TidySBMLWriter.write(doc, new File(args[2]), '␣', (short) 2);
  }

  /**
   *
   * @param listOfParticipants
   * @param species
```

```
   * @param id
   */
  private static void setId(ListOf<SpeciesReference> listOfParticipants,
    String species, String id) {
    species = createNewReference(species, 'M');
    for (SpeciesReference sr : listOfParticipants) {
      if (sr.getSpecies().equals(species)) {
        sr.setId(id);
        System.out.println("Setting id to " + id + " for speciesReference to " + species);
        break;
      }
    }
  }

  /**
   *
   * @param ref
   * @param pref
   * @return
   */
  private static String createNewReference(String ref, char pref) {
    String newRef;
    if (ref.charAt(0) == '_') {
      newRef = pref + ref;
    } else {
      newRef = pref + "_" + ref;
    }
    return newRef;
  }
```

## 5.2 Preparation of experimental data

In 2012, Bergdahl et al. published time-course metabolite profiling in yeast as part of their work in *Biotechnology for Biofuels*. Let us see what happens when we load this laboratory data set onto our network. First, we need to adjust the data a bit so that SBMLsimulator can read them in. The original dataset is a Microsoft Excel spreadsheet that contains several tabs. Let us go to the tab of raw intracellular concentrations. There are eleven repeated time points with measurements taken for several compounds, some of which are so-called NaN values, indicating a missing value. For our purposes, we need precisely one value per time point. To this end, we create a new table below, where we calculate the median value of every measurement. Thereby, we skip all NaN values. As the table head, we use the compound identifier from above but prefixed with `M_`. In this way, the ids fit those in the SBML model and make it easier to map the data to our network graph. There is one compound in the data set for which the model does not have any counterpart. So, we can skip it in our new table, but SBMLsimulator would also automatically ignore a column without a

**Listing 5.3** | First reaction from the merged SBML file with a layout for iMM904

```
<listOfReactions>
  <reaction fast="false" fbc:lowerFluxBound="cobra_0_bound"
      fbc:upperFluxBound="cobra_default_ub" id="R_13BGH" metaid="R_13BGH"
      name="Endo␣1␣3␣beta␣glucan␣glucohydrase" reversible="false"
      sboTerm="SBO:0000375">
    <annotation>
      <rdf:RDF xmlns:rdf="http://www.w3.org/1999/02/22-rdf-syntax-ns#"
          xmlns:bqbiol="http://biomodels.net/biology-qualifiers/">
        <rdf:Description rdf:about="#R_13BGH"><bqbiol:is><rdf:Bag><rdf:li
          rdf:resource="http://identifiers.org/bigg.reaction/13BGH"/><rdf:li
          rdf:resource="http://identifiers.org/metanetx.reaction/MNXR94686"/>
        </rdf:Bag></bqbiol:is></rdf:Description>
      </rdf:RDF>
    </annotation>
    <fbc:geneProductAssociation
        xmlns:fbc="http://www.sbml.org/sbml/level3/version1/fbc/version2">
      <fbc:geneProductRef fbc:geneProduct="G_YGR282C"/>
    </fbc:geneProductAssociation>
    <listOfReactants>
      <speciesReference constant="true" sboTerm="SBO:0000010"
          species="M_13BDglcn_c" stoichiometry="1"/>
      <speciesReference constant="true" sboTerm="SBO:0000010"
          species="M_h2o_c" stoichiometry="1"/>
    </listOfReactants>
    <listOfProducts>
      <speciesReference constant="true" sboTerm="SBO:0000011"
          species="M_glc__D_c" stoichiometry="1"/>
    </listOfProducts>
  </reaction>
```

corresponding model component. Since SBML assumes initial time points to be 0, we also shift all times values in our data set by 14½ time-units. We now save this new table in a CSV file that matches the description of the expected format from listing 4.1. Listing 5.4 shows a part of the resulting yeast data set.

**Listing 5.4** | Part of the example yeast data set in CSV format

```
#time   M_g6p_c      M_r5p_c      M_f6p_c      M_glyc3p_c   ...
0       2.665226007  0.126552935  0.499107017  0.574605606  ...
1.5     1.613476335  0.102134032  0.362049424  0.321757941  ...
3       1.425163287  0.184214787  0.281096754  0.312816984  ...
...     ...          ...          ...          ...          ...
24      0.452652433  0.073917444  0.162222695  0.143999990  ...
```

## 5.3 Summary

We briefly discussed how to convert different model file formats. A few of these steps might still seem a bit complicated and require some degree of code writing. But we can expect that more models with embedded layouts will become available as also their software support is steadily improving. We also discussed how to transform experimental data using Microsoft Excel. In the next chapter, we will import both generated files to SBMLsimulator and fully explore its visualization capabilities.

# 6 Visualization of manually created layouts

In the previous example, we embedded a manually created metabolic map of the central carbon metabolism of the baker's yeast in the SBML file iMM904 (Mo et al., 2009). Next, we prepared a character-separated value file from the time-course data set that Bergdahl et al. published in 2012. We can now visually analyze a model that comes with embedded layout information and explore a real data set from laboratory experiments in the context of this model.

☞ Watch this example online at /qv3qPyzofhI.

## 6.1 Opening a constraint-based model with a graph layout

Let us load this new model into SBMLsimulator. We now see the warning dialog depicted in fig. 6.1 that many model components lack initial values. This only matters when running a dynamic simulation. Since we plan to use SBMLsimulator to create an animation of experimentally measured data, we do not need to run an Ordinary Differential Equation (ODE)-based simulation and can safely ignore this warning.

The Graph tab now shows us the same neat display that we already saw on the BiGG Models Database (King et al., 2015b; Norsigian et al., 2019). Now, let us find out how to create a dynamically animated network of yeast.

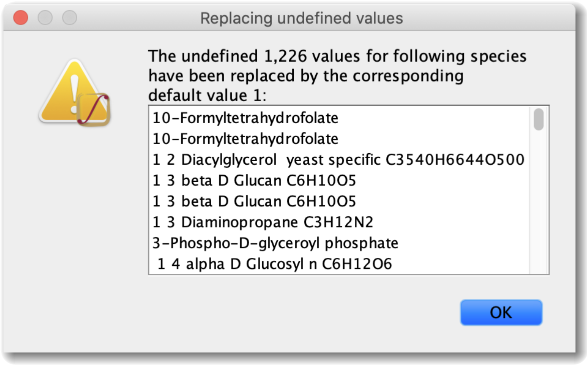


**Figure 6.1** | Warning when loading a model with undefined initial values. Constraint-based models, such as iMM904, can be simulated in a flux-balance framework that does not require initial amounts to be known for reactive species. This warning indicates that the model cannot directly be simulated in an Ordinary Differential Equation (ODE) framework. SBMLsimulator automatically assigns the value 1 where missing. It is also possible to estimate missing values using EvA2.

## 6.2 Preparation of experimentally measured data

When we now drop our time-series data file into SBMLsimulator as described in section 4.1.2, it displays an import dialog asking us to confirm the matching of data columns to model components (fig. 4.2). Since we prepared everything already, there is nothing left to do. In case our model identifiers do not precisely match those in the CSV file, we have here the option to adjust the mapping. The tab for Experimental Data is now enabled where we can see the same table that we had prepared (with a display similar to fig. 3.8).

## 6.3 Interactive visualization of data on a network map

Let us switch off the status bar, and controls in the view menu View > Show options and View > Show Status Bar because we do not need them for graph analysis. Now we will have more space for the display of the network diagram.

The Graph tab has automatically loaded the data set and mapped the values to the nodes. We can select our favorite visualization of the data. Available are node sizes or colors in absolute values or relative to minimum and maximum in the data set. Displaying the relative fill level in combination with absolute color values as fig. 6.2 shows is a special recommendation.

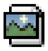

By clicking the icon next to this text, we can create an image file of the current state of the graph in the vector format Scalable Vector Graphics (SVG), or in Portable Network Graphics (PNG) format that we can save to a file. We can customize the color scheme and other visual characteristics, such as the minimal and maximal node sizes, in the settings dialog. Since we can adjust many visual attributes while the animation is still ongoing, this really is a seamless animation.

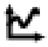

When we click the button with the icon shown next to this text, we see a diagram of all variables within our dataset and a red vertical bar indicating the current position moving in sync with our animation (fig. 6.3). This view allows us to compare different visual representations and to directly observe the most active spots in the network.

Another exciting feature is the sliding camera animation (also known as *Ken Burns effect*) that we can activate by selecting a speed from this drop-down box. To design such an animation, we need to create a CSV file that contains in one line the zoom level followed by a tab-separated list of corner points. These are the *x*- and *y*-coordinates of the top-left corners that define the path of the visible sliding view-port window. In other words, these comma-separated coordinates define where the camera makes a turn on its way. Listing 6.1 gives us an example of such a file for an animation using the *Ken Burns effect*. To enable the camera animation, we go to settings and navigate to the bottom of the tab Network dynamics.

**Listing 6.1** | Definition of a moving camera animation for a *Ken Burns effect*

```
0.25	0,0	200,50	800,350	800,800	800,2600
```

Finally, we can export the animation that we just created to a movie file by clicking the button with the icon shown next to this text. This gives us first a warning because of the high resolution and demanding computation. Hence, the export may take some time and consume some space on our hard drive. The export supports a wide range of common movie file types, including Audio Video Interleave (AVI), QuickTime File Format (MOV), Moving Picture Experts Group (MPG), and others.

## 6.4 Summary

In this tutorial, we visualized manually created layouts of experimental data. The examples demonstrated in this text are also available online at `/systemsbiology/` together with videos that were created using these features. There you can also get insides into the analysis of actual biological data sets and explore other fascinating examples of dynamic metabolic networks.

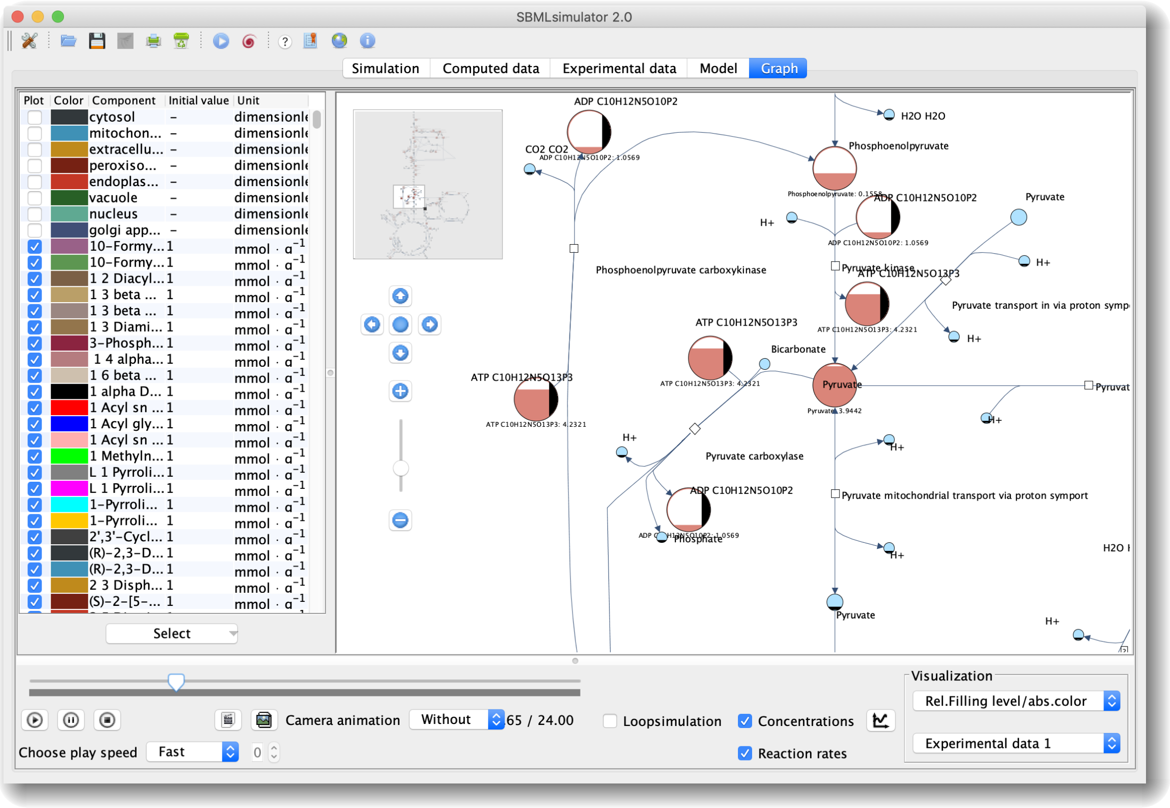


**Figure 6.2** | Graph animation. We can zoom in and use the slider at the bottom to move to time points during the animation that we are most interested in. The check-box labeled "Loopsimulation" allows us to create a repeated animation. While the animation is running, and we can pan and zoom with the graph or change its visualization style. The drop-down list "Choose play speed" allows us to adjust how fast the animation should run in three different levels: normal, fast, or slow. If we want to display the exact values of metabolite concentrations and so forth, we just need to check the boxes to the right that are labeled "Concentrations" or "Reaction rates."

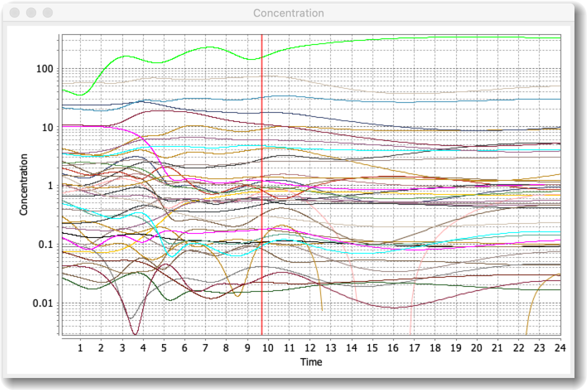


**Figure 6.3** | Animated plot. This view appears when clicking the diagram icon in the panel at the bottom of fig. 6.2. It displays all variables in the network for which data are available in the form of a conventional plot, here shown for the data set by Bergdahl et al. applied to iMM904 (Mo et al., 2009). The red vertical bar is moving with the animation and indicates the current time point.

# 7 Command-line arguments and preferences

It now follows a short overview of all possible command-line options of SBMLsimulator. All primary functions of the program are also available from the command line. This means that we can also run SBMLsimulator on a cluster and use it for large-scale model calibration. Furthermore, we can use the command-line options in combination with the GUI. One possible use-case scenario would be to launch the program with our desired configuration with a customized start script, e.g., to directly open a specific model file when launching the application.

## 7.1 Simulator input/output options

### 7.1.1 Select input files

These options are used to select all input files for the simulation.

`--sbml-input-file[ |=]<File>` Select a model in SBML format that is to be simulated. Accepts SBML files (`*.sbml`, `*.xml`). Default value: none

`--time-series-file[ |=]<File>` Path to a file with a time series of species, compartment, or parameter values. Accepts CSV files (`*.csv`). Default value: none

### 7.1.2 Select output files

Select output files for the results of simulation and parameter estimation.

`--sbml-output-file[ |=]<File>` Select a file where to store the model in SBML format after parameter optimization. Accepts SBML files (`*.sbml`, `*.xml`). Default value: none

`--simulation-output-file[ |=]<File>` Select a file where to store the results of a simulation. Accepts CSV files (`*.csv`). Default value: none

## 7.2 Simulation options

### 7.2.1 Missing values

Decide how to treat the values of compartments, species, and parameters if no initial value has been defined in the model. A missing initial value is to be distinguished from invalid, i.e., NaN values. Furthermore, the elements may have very diverse units associated with them. Here it is only possible to define one numeric value for each element group, irrespective of any unit.

--default-init-compartment-size[ |=]<Double> If not specified, the value corresponding to this argument will be used to initialize the size of compartments. Arguments must fit into the range $(0.0, 9 \cdot 10^9]$. Default value: `1.0`

--default-init-species-value[ |=]<Double> If not specified, the value corresponding to this argument will be used to initialize species depending on their `hasOnlySubstanceUnits` property as the initial amount or initial concentration. Arguments must fit into the range $(0.0, 9 \cdot 10^9]$. Default value: `1.0`

--default-init-parameter-value[ |=]<Double> The default initial-value that is set for parameters with an undefined value. Arguments must fit into the range $(0.0, 9 \cdot 10^9]$. Default value: `1.0`

### 7.2.2 Settings for the simulation

Here we can specify parameters for the simulation of the model.

--ode-solver[ |=]<Class> This gives the class name of the default solver for Ordinary Differential Equation (ODE) systems. The associated class must implement `AbstractDESSolver` and must have a constructor without any parameters. All possible values for type `<Class>` are:

- `org.simulator.math.odes.AdamsBashforthSolver`,
- `org.simulator.math.odes.AdamsMoultonSolver`,
- `org.simulator.math.odes.DormandPrince54Solver`,
- `org.simulator.math.odes.DormandPrince853Solver`,
- `org.simulator.math.odes.EulerMethod`,
- `org.simulator.math.odes.GraggBulirschStoerSolver`,
- `org.simulator.math.odes.HighamHall54Solver`,
- `org.simulator.math.odes.RosenbrockSolver`, and
- `org.simulator.math.odes.RungeKutta_EventSolver`.

Default value: `org.simulator.math.odes.RungeKutta_EventSolver`

--abs-tol[ |=]<Double> Allowed absolute vectorial error. Default value: `1.0E-10`

--rel-tol[ |=]<Double> Allowed relative vectorial error. Default value: `1.0E-6`

--sim-start-time[ |=]<Double> The double value associated with this key must, in case of SBML, equal to zero. Generally, any start time would be possible. This is why this key exists. But SBML is defined to start its simulation at the time zero. Arguments must fit into the range $[0, 10^5]$. Default value: `0.0`

`--sim-end-time[ |=]<Double>` With the associated non-negative double number that has to be greater than 0 when simulating SBML models, it is possible to perform a simulation. Arguments must fit into the range $(0, 10^5]$. Default value: `5.0`

`--sim-step-size[ |=]<Double>` The greater this value, the longer the computation time, but the more accurate the result will be. Arguments must fit into the range $(0, 10^5]$. Default value: `0.01`

## 7.3 Estimation Options

### 7.3.1 Spline approximation

These options allow us to estimate parameters to spline interpolation values between given measurement data and to configure how to calculate these splines.

`--fit-to-splines` If this is selected, splines will be calculated from given experimental data, and the parameter estimation procedure will fit the system to the splines instead of the original values. The advantage of this procedure is that the amount of available data is increased due to this form of interpolation, also ensuring that the shape of the resulting curves comes close to what could be expected. The disadvantage is that the influence of potential outliers on the overall fitness is increased. Default value: `false`

`--number-of-spline-samples[ |=]<Integer>` This defines the number of additional spline sampling points between the measurement data. If we select zero, only the real sampling points will be used. Default value: `50`

### 7.3.2 Default optimization targets

Select the default optimization targets.

`--est-all-compartments` Decide whether or not by default, all compartments in a model should be considered the target of optimization, i.e., value estimation. Default value: `false`

`--est-all-global-parameters[ |=]<Boolean>` Decide whether or not by default, all global parameters in a model should be considered the target of optimization, i.e., value estimation. Default value: `true`

`--est-all-local-parameters[ |=]<Boolean>` Decide whether or not by default, all local parameters in a model should be considered the target of optimization, i.e., value estimation. Default value: `true`

`--est-all-species` Decide whether or not by default, all species in a model should be considered the target of optimization, i.e., value estimation. Default value: `false`

`--est-all-undefined-quantities` Estimates the values for all those quantities in the model whose values are either undefined or set to NaN. Default value: `false`

### 7.3.3 Ranges of all optimization targets

Define the initial and absolute ranges of all optimization targets.

`--est-init-min-value[ |=]<Double>` The minimal value of the initialization range in a parameter estimation procedure. Default value: `0.0`

`--est-init-max-value[ |=]<Double>` The maximal value of the initialization range in a parameter estimation procedure. Default value: `10.0`

`--est-min-value[ |=]<Double>` The minimal value in the full allowable range in a parameter estimation procedure. Default value: `0.0`

`--est-max-value[ |=]<Double>` The maximal value in the full allowable range in a parameter estimation procedure. Default value: `1000.0`

### 7.3.4 Integration strategy

Select whether or not to apply a multiple shooting strategy.

`--est-multi-shoot[ |=]<Boolean>` Decide whether a model calibration should be done using multiple shoot technique. This should be the default. The other possibility is the so-called single shoot technique. This means that only one initial value is taken to integrate the ordinary differential equation system. In contrast, the multiple shoot technique restarts the integration in each time step, given the values in this step. The aim is then to come as close as possible to the start value in the next time step. In many cases, the fitness landscape becomes much more friendly when using a multiple shoot strategy. Default value: `true`

`--use-existing-solution` Select whether or not to use the parameters in the existing model for post-optimization. Default value: `false`

### 7.3.5 Quality function

Here we can specify how to evaluate the quality of a parameter set to given experimentally measured data.

`--quality-measure[ |=]<Class>` This specifies the class name of the default quality function that evaluates the quality of the simulation for provided (experimental) data. All possible values for type <Class> are:

- `org.simulator.math.EuclideanDistance`,
- `org.simulator.math.ManhattanDistance`,

- org.simulator.math.N_Metric,
- org.simulator.math.PearsonCorrelation,
- org.simulator.math.RelativeEuclideanDistance,
- org.simulator.math.RelativeManhattanDistance,
- org.simulator.math.RelativeSquaredError, and
- org.simulator.math.Relative_N_Metric.

Default value: org.simulator.math.RelativeSquaredError

--quality-default-value[ |=]<Double> The default return value of a quality function that can be used if, for some reason, the quality cannot be computed. For example, this might avoid a division by zero. Default value: 1000.0

--quality-n-metric-root[ |=]<Double> The root parameter in the distance function for *n*-metrics: in the case of the *n*-norm, this is at the same time also the exponent. For instance, the Euclidean distance has a root value of two, whereas the Manhattan norm has a root of one. In the RSE, the default root is also two, but this value may be changed. Default value: 3.0

### 7.3.6 Additional options

--est-targets[ |=]<String> The file with the values to estimate and their initial setting.

## 7.4 Options for the graphical user interface

--check-for-updates[ |=]<Boolean> Decide whether or not this program should search for updates at start-up. Default value: true

--gui If this option is given, the program will display its GUI. Default value: false

--log-level[ |=]<String> Change the log-level of this application. This option will influence how fine-grained error and other log messages will be that we receive while executing this program. All possible values for type <String> are: ALL, CONFIG, FINE, FINER, FINEST, INFO, OFF, SEVERE, and WARNING. Default value: INFO

## 7.5 Plot Options

### 7.5.1 Plotting panel options

Options for the visible rectangle of the plot panel.

--show-plot-grid Decide whether or not to draw a grid in the plot area. Default value: false

--show-plot-legend[ |=]<Boolean> Add or remove a legend in the plot. Default value: true

--show-plot-tooltips Let the plot display tooltips for each curve. Default value: false

### 7.5.2 Appearance of the plot

Options for the appearance of the plot area.

--plot-background-color[ |=]<Color> The background color of the plot Default value: java.awt.Color[r=255,g=255,b=255] (white)

--plot-grid-color[ |=]<Color> The color of the plot's grid Default value: java.awt.Color[r=64,g=64,b=64] (anthracite)

## 7.6 CSV options

### 7.6.1 CSV file selection

Select the character-separated file to be opened.

--csv-file[ |=]<File> A CSV file to be opened. Default value: none

### 7.6.2 CSV file characters

Define the special characters in CSV files that separate values or that quote strings of characters.

--csv-files-separator-char[ |=]<Character> The separator character that is written between the entries of a character separated value file. Not that actually any Unicode Transformation Format (UTF)-8 character can be used as a separator, not only commas. All possible values for type <Character> are: ⇥, ␣, ,, /, ;, and |. Default value: ,

--csv-files-quote-char[ |=]<Character> The character that is used to quote strings inside of CSV files to prevent a miss-interpretation of white spaces with separator characters. Default value: "

## 7.7 Garuda options

Settings for the communication with a Garuda Core and hence access to other Garuda gadgets.

--connect-to-garuda[ |=]<Boolean> Decides whether or not the current application should attempt to connect to the Garuda Core (Ghosh et al., 2011). Default value: true

# 8 License

SBMLsimulator is free software: You can redistribute it and/or modify it under the terms of the GNU Lesser General Public License as published by the Free Software Foundation, either version 3 of the License, or (at your option) any later version.

This program is distributed in the hope that it will be useful, but **WITHOUT ANY WARRANTY**; without even the implied warranty of **MERCHANTABILITY** or **FITNESS FOR A PARTICULAR PURPOSE**. See the GNU General Public License for more details.

You should have received a copy of the GNU Lesser General Public License along with this program. If not, see `http://www.gnu.org/licenses/lgpl-3.0-standalone.html`.

## 8.1 Included third-party libraries and packages

SBMLsimulator includes and redistributes the following software packages:

- JSBML (Dräger et al., 2011; Rodriguez et al., 2015), which is freely available under the terms of the GNU Lesser General Public License (LGPL)version 2.1, see `http://sbml.org/Software/JSBML`.
- the Systems Biology Simulation Core Library (SBSCL) (Keller et al., 2013), which is licensed under the LGPLversion 3.1, see `http://simulation-core.sourceforge.net`.
- the nature-inspired heuristic optimization framework EvA2 (LGPLversion 3, Kronfeld et al., 2010), see `http://www.cogsys.cs.uni-tuebingen.de/software/EvA2`.
- Apache Commons-math under the terms of the Apache Software License, Version 2.0, see `http://commons.apache.org/math/`.
- JCommon, licensed under LGPLversion 2.1, see `http://jfree.org/jcommon`.
- JFreeChart, licensed under LGPLVersion 3.1, see `http://www.jfree.org/jfreechart`.
- Icons: `www.pixel-mixer.com`.
- macOS support: Quaqua Look and Feel, license LGPLversion 2.1, see `http://www.randelshofer.ch/quaqua/`.

# 9 FAQ / Troubleshooting

**Where can I get help for a certain component/ option/ check-box/ etc.?**
Most elements in SBMLsimulator have tool-tips. If you do not understand an option, you can get help in the first place by just pointing the mouse cursor over it and wait for the tool-tip to show up ($\sim$ 3 seconds).

**I'm getting the message** `java.lang.OutOfMemoryError: Java heap space`
Some operations need a lot of memory. If you simply start SBMLsimulator, without any Java™ Virtual Machine (JVM) parameters, only 64 MB of memory is available. Please append the argument `-Xmx1024M` to start the application with 1 GB of main memory. See section 2.2 for a more detailed description of how to start the application with additional memory. If possible, you should give the application 2 GB of main memory. A minimum of 1 GB main memory should be available to the application.

**Is an internet connection required to run SBMLsimulator?**
An internet connection is not required for simulations, but for some other operations, like the online-update feature.

**Where can I get the latest version?**
Go to `/draeger-lab/SBMLsimulator/`.

**Which Java™ version must be installed on my computer to launch SBMLsimulator?**
SBMLsimulator requires at least Java™ 8. Please see `https://jdk.java.net` to download the latest Java™ version.

**Why does SBMLsimulator not start on my Mac with macOS before 10.6 Update 3?**
If you try to launch SBMLsimulator, but the application does not start, and you receive the following error message on the command-line or Java™ console of your Mac, you need to update your Java™ installation:

```
Exception in thread "AWT-EventQueue-0" java.lang.NoClassDefFoundError:
    com/apple/eawt/AboutHandler
    at java.lang.ClassLoader.defineClass1(Native Method)
    at java.lang.ClassLoader.defineClass(ClassLoader.java:703)
    ...
```

The interface `com.apple.eawt.AboutHandler` was introduced to Java™ for macOS 10.6 Update 3. If you have an earlier version of macOS or Java™ , please update your operating system or Java™ installation. Also, see the macOS documentation about the `AboutHandler` for more information. On a Mac, you can update your Java™ installation through the Software Update menu item in the main Apple menu.

**How can I report bugs or get help?**
The best way to get in touch is by using the issue tracker at `/draeger-lab/SBMLsimulator/issues`.

# Appendix A

# Acknowledgments

Many people and organizations contributed to the project or funded our work over the years. We would like to thank all those whose effort has been put into this endeavor.

## A.1 Alumni

Lea F. Buchweitz[1] Julian Heinrich[1], Christoph M. Blessing[1], Veronika Kohler[1], Alexander Dörr[1], Roland Keller[1], Marcel Kronfeld[1], Hannes Planatscher[2], Adrian Schröder[1], Philip Stevens[1], Dieudonné Motsou Wouamba[1], Max Zwießele[1] Meike Aichele[1], Robin Fähnrich[1], Fabian Becker[1], Stefan Fischer[1], Simon Schäfer[1], Hannes Borch[1]

## A.2 Collaborators

James T. Yurkovich[3], Fabian Schwarzkopf[4], Zachary A. King[5], Laurence Yang[6], Freyr Jóhannsson[7], Ólafur E. Sigurjónsson[8][9], Óttar Rolfsson[7]

## A.3 Contributors

Stephanie Hoffmann[1], Mike Cooling[10], Akira Funahashi[11], Nicolas Rodriguez[12], Akito Tabira[11], Noriko Hiroi[11] Michael J. Ziller[13]

[1]University of Tübingen, Tübingen, Germany
[2]Signatope GmbH, Reutlingen, Germany
[3]Institute for Systems Biology, Seattle, WA, United States
[4]yWorks GmbH, Tübingen, Germany
[5]Department of Bioengineering, University of California, San Diego, La Jolla, CA, United States
[6]Queen's University, Kingston ON, Canada
[7]Center for Systems Biology, University of Iceland, Reykjavík, Iceland
[8]The Blood Bank, Landspítali-University Hospital, Reykjavík, Iceland
[9]School of Science and Engineering, Reykjavík University
[10]Auckland Bioengineering Institute, University of Auckland, New Zealand
[11]Keio University Graduate School of Science and Technology, Yokohama, Japan
[12]European Bioinformatics Institute, Wellcome Trust Genome Campus, Hinxton, United Kingdom
[13]Translational Research in Psychiatry, Max Planck Institute of Psychiatry

## A.4 Special thanks

We like to thank Bernhard Ø. Palsson[5] and Andreas Zell[1] together with their research groups for supporting this work.

For testing and using SBMLsimulator, we thank Benjamin Kandel[14], Marcus Klein[14], Ute Hofmann[14], Maria Thomas[14], Nicolas Le Novère[15],

For friendly help, we thank Beky Kotcon[16], Samantha Mesuro[16], Daniel Rozenfeld[16], Anak Yodpinyanee[16], Andres Perez[16], Eric Doi[16], Richard Mehlinger[16], Steven Ehrlich[16], Martin Hunt[16], George Tucker[16], Peter Scherpelz[16], Aaron Becker[16], Eric Harley[16], and Chris Moore[16].

Thanks for support also to the former team from the Center for Bioinformatics Tuebingen (ZBIT): Finja Wrzodek[1], Florian Mittag[1], Sebastian Nagel[1], and Clemens Wrzodek[1].

## A.5 Funding

This work was funded by the National Institutes of Health (NIH, US, grants № R01-GM070923 and U01-GM102098), the Institute for Systems Biology's Translational Research Fellowship (to James T. Yurkovich), the Landspítali University Hospital Research Fund, the University of Iceland Research Fund, and the Novo Nordisk Foundation through the Center for Biosustainability at the Technical University of Denmark (grant № NNF10CC1016517).

This work was made possible by the friendly support of yWorks GmbH[17], who provided their diagram visualization library yFiles for Java™[18] and assistance during the implementation phase.

We also thank the Google Summer of Code program[19] for supporting open-source software development for this project.

Early versions of this software were funded by the Federal Ministry of Education and Research (BMBF, Germany) in the project Virtual Liver Network (grant № 0315756) and the European Commission as part of a Marie Curie International Outgoing Fellowship within the EU 7th Framework Program for Research and Technological Development (project AMBiCon, grant № 332020).

[14]Dr. Margarethe Fischer-Bosch-Institute for Clinical Pharmacology (IKP), Stuttgart, Germany
[15]The Babraham Institute, Cambridge, UK
[16]Harvey Mudd College, Claremont, California, United States
[17]`https://www.yworks.com`
[18]`https://www.yworks.com/products/yfiles-for-java`
[19]`https://summerofcode.withgoogle.com`

# Appendix B

# Release notes

## B.1 Version 1.0

This is the first release from March 1$^{st}$ 2012.

## B.2 Version 1.1

This version was released at April 25$^{th}$ 2013.

### B.2.1 New features

Includes version 1.3 of the Systems Biology Simulation Core Library (SBSCL) (Keller et al., 2013).

### B.2.2 Bug fixes

All issues related to the simulation of SBML files that were fixed in the Systems Biology Simulation Core Library (SBSCL).

## B.3 Version 1.2

This third release was announced at April 24$^{th}$ 2014.

### B.3.1 New features

- ✔ The estimation targets and their ranges can now be saved into a file and loaded again.
- ✔ Spline interpolation can now be used before optimization.
- ✔ The program has been updated and is now based on the new version 1.4 of the Systems Biology Simulation Core Library (SBSCL) and JSBML version 1.0β1.
- ✔ Support for Garuda has been added.

- ✔ Improved support for macOS
- ✔ Program update to Java™ version 1.6

### B.3.2 Bug fixes

- ✖ Some errors in the command line mode have been corrected.
- ✖ There was an error in optimization when time point 0 is not given.

## B.4 Version 1.2.1

This is a minor bug-fix release from July 18th 2014.

### B.4.1 New features

- ✔ Now a new model can be selected to be opened without closing the current model. The active model can still be saved before being closed.

### B.4.2 Bug fixes

- ✖ There was an error in the data importer: columns were not correctly skipped, if chosen by the user.

## B.5 Version 2.0

### B.5.1 New features

- ✔ Support for SBML files up to the latest release of Level 2 Version 5 (Hucka et al., 2015) and Level 3 Version 2 (Hucka et al., 2019) thanks to the updated JSBML library (Dräger et al., 2011; Rodriguez et al., 2015): The graphical user interface was extended to support new model components, including various SBML extension packages, such as Groups (Hucka and Smith, 2016), Layout (Gauges et al., 2015, see further description below), Flux Balance Constraints (Olivier and Bergmann, 2015), etc.
- ✔ Improved simulation capabilities using release 1.5 of the simulation backend Systems Biology Simulation Core Library (SBSCL) (Keller et al., 2013).
- ✔ Garuda (Ghosh et al., 2011) support: SBMLsimulator 2.0 comes with a Garuda archive file and can, therefore, be directly used from the Garuda workbench.

- ✔ Support for model displays as SBGN Process Diagrams (Rougny et al., 2019) using yFiles[1] for Java™ :
  - If a model contains a diagram (in the format of the SBML Layout Extension Gauges et al., 2015), SBMLsimulator 2.0 can now display this layout in an interactive map.
  - SBMLsimulator automatically generates a map representation for models without graphical information.
  - In either case, the new tab `Graph` can be clicked to access the model's display.
- ✔ Mapping of experimental or simulation data to an interactive map: The model display (be it defined in the model or automatically generated) takes simulation data (the result of a numerical calculation) or loaded experimental data and maps it to the nodes and arrows.
  - Several different ways of data mapping have been implemented and are accessible from the graphical user interface: node size, node color, absolute size and relative color, relative size and absolute color values, relative filling levels with absolute color gradients. Further details are described in the article by Buchweitz et al. (2018).
  - An export function allows users to save the display as an image, e.g., for publication or analysis in scalable vector format (Scalable Vector Graphics (SVG)) or as a portable network graphics file (Portable Network Graphics (PNG)).
  - Time-series data can be visualized as a dynamically animated video that can be played in several speeds. While the video is playing, users can zoom and pan on the map and analyze areas of interest. To this end, the animation can play in an ongoing loop. Optionally, the exact values of fluxes and concentrations can be displayed directly on the graph.
  - An animated sliding camera window can automatically move the viewport through the graph while the animation is playing. To this end, users can define coordinates in a simple text file format. The user can also adjust the speed of the sliding window.
  - Optionally, a diagram with a moving red line can be displayed while the animation is running to indicate where in the diagram the animation is.
  - An export function saves the generated animations to a variety of standard video file formats, including MOV, AVI, MPG, Windows Media Video (WMV), Flash Video (FLV).

### B.5.2 Bug fixes

- ✖ The application has generally become more stable and sophisticated, thanks to updated third-party apps and minor improvements.

[1] `https://yworks.com`

# Appendix C

# Acronyms

**API** Application Programming Interface . . . iii
**AVI** Audio Video Interleave . . . 33
**BiGG** Biochemically, Genomically, and Genetically structured
**CSV** Character-Separated Value . . . iii
**DE** Differential Evolution . . . 14
**ES** Evolution Strategies . . . 14
**FLV** Flash Video . . . 48
**GA** Genetic Algorithms . . . 14
**GEM** Genome-scale Metabolic Model . . . 24
**GUI** Graphical User Interface . . . iii
**Id** Identifier . . . 5
**JAR** Java™ Archive . . . 3
**JDK** Java™ Development Kit . . . 3
**JVM** Java™ Virtual Machine . . . iii
**JSON** JavaScript Object Notation . . . 24
**LGPL** GNU Lesser General Public License . . . 41
**MIRIAM** Minimal Information Required In the Annotation of Models . . . 24
**MOV** QuickTime File Format . . . 33
**MPG** Moving Picture Experts Group . . . 33
**NaN** Not a Number . . . 14
**ODE** Ordinary Differential Equation . . . vi
**PDF** Portable Document Format . . . 12
**PNG** Portable Network Graphics . . . 32
**PSO** Particle Swarm Optimization . . . 14
**RAM** Random-Access Memory . . . 4
**RSE** Relative Squared Error . . . 16
**SBGN** Systems Biology Graphical Notation . . . iii
**SBML** Systems Biology Markup Language . . . iii
**SBSCL** Systems Biology Simulation Core Library . . . iii

| | | |
|---|---|---|
| **SVG** | Scalable Vector Graphics | 32 |
| **UTF** | Unicode Transformation Format | 40 |
| **WMV** | Windows Media Video | 48 |

# Bibliography


Apache Software Foundation. Commons Math: The Apache Commons Mathematics Library. http://commons.apache.org/proper/commons-math/, June 2013. URL http://commons.apache.org/proper/commons-math/. Accessed: 2013-06-24.

Basti Bergdahl, Dominik Heer, Uwe Sauer, Bärbel Hahn-Hägerdal, and Ed Wj van Niel. Dynamic metabolomics differentiates between carbon and energy starvation in recombinant saccharomyces cerevisiae fermenting xylose. *Biotechnology for Biofuels*, 2012. ISSN 17546834. doi:10.1186/1754-6834-5-34.

Joachim Bucher, Stephan Riedmaier, Anke Schnabel, Katrin Marcus, Gabriele Vacun, Thomas S. Weiss, Wolfgang E. Thasler, Andreas K. Nüssler, Ulrich M. Zanger, and Matthias Reuss. A systems biology approach to dynamic modeling and inter-subject variability of statin pharmacokinetics in human hepatocytes. *BMC Systems Biology*, 5(1):66, May 2011. ISSN 1752-0509. doi:10.1186/1752-0509-5-66. URL http://dx.doi.org/10.1186/1752-0509-5-66.

Lea F. Buchweitz, Christoph Yurkovich, James T. Blessing, Veronika Kohler, Fabian Schwarzkopf, Zachary A. King, Laurence Yang, Freyr Jóhannsson, Ólafur Sigurjónsson, Óttar Rolfsson, Julian Heinrich, and Andreas Dräger. Visualizing metabolic network dynamics through time-series metabolomics data. *bioRxiv*, September 2018. doi:10.1101/426106.

Maurice Clerc. *Particle Swarm Optimization*. ISTE Ltd, London, UK, January 2006a. ISBN 9781905209040. doi:10.1002/9780470612163. URL https://onlinelibrary.wiley.com/doi/book/10.1002/9780470612163.

Maurice Clerc. *TRIBES or Cooperation of Tribes*, pages 139–149. ISTE Ltd, January 2006b. doi:10.1002/9780470612163.ch11. URL https://onlinelibrary.wiley.com/doi/book/10.1002/9780470612163.

Maurice Clerc and James Kennedy. The Particle Swarm—Explosion, Stability, and Convergence in a Multidimensional Complex Space. *IEEE Transactions on Evolutionary Computation*, 6(1):58–73, 2002.

Alexander Dörr, Roland Keller, Andreas Zell, and Andreas Dräger. SBMLsimulator: a Java tool for model simulation and parameter estimation in systems biology. *Computation*, 2(4):246–257, December 2014. ISSN 2079-3197. doi:10.3390/computation2040246. URL http://www.mdpi.com/2079-3197/2/4/246.

Andreas Dräger, Marcel Kronfeld, Michael J. Ziller, Jochen Supper, Hannes Planatscher, Jørgen B. Magnus, Marco Oldiges, Oliver Kohlbacher, and Andreas Zell. Modeling metabolic networks in *C. glutamicum*: a comparison of rate laws in combination with various parameter optimization strategies. *BMC Systems Biology*, 3(5):5, January 2009. doi:10.1186/1752-0509-3-5. URL `http://www.biomedcentral.com/1752-0509/3/5`.

Andreas Dräger, Nicolas Rodriguez, Marine Dumousseau, Alexander Dörr, Clemens Wrzodek, Nicolas Le Novère, Andreas Zell, and Michael Hucka. JSBML: a flexible Java library for working with SBML. *Bioinformatics*, 27(15):2167–2168, August 2011. doi:10.1093/bioinformatics/btr361. URL `http://dx.doi.org/10.1093/bioinformatics/btr361`.

Akira Funahashi, Yukiko Matsuoka, Akiya Jouraku, Mineo Morohashi, Norihiro Kikuchi, and Hiroaki Kitano. CellDesigner 3.5: A versatile modeling tool for biochemical networks. *Proceedings of the IEEE*, 96(8):1254–1265, July 2008. ISSN 00189219. doi:10.1109/JPROC.2008.925458.

Ralph Gauges, Ursula Rost, Sven Sahle, Katja Wengler, and Frank T. Bergmann. The Systems Biology Markup Language (SBML) Level 3 Package: Layout, Version 1 Core. *Journal of Integrative Bioinformatics*, 12(2):267, September 2015. doi:10.2390/biecoll-jib-2015-267.

Samik Ghosh, Yukiko Matsuoka, Yoshiyuki Asai, Kun-Yi Hsin, and Hiroaki Kitano. Software for systems biology: from tools to integrated platforms. *Nature Reviews Genetics*, 12(12):821–832, December 2011. URL `http://www.nature.com/nrg/journal/v12/n12/abs/nrg3096.html`.

John H. Holland. *Adaptation in Natural and Artificial Systems*. The University of Michigan Press, Cambridge, MA, USA, 1975. ISBN 0-262-58111-6.

Michael Hucka and Lucian P Smith. SBML Level 3 package: Groups, Version 1 Release 1. *Journal of Integrative Bioinformatics*, 13(3):8–29, September 2016. doi:10.1515/jib-2016-290. URL `https://doi.org/10.1515/jib-2016-290`.

Michael Hucka, Andrew Finney, Herbert M. Sauro, Hamid Bolouri, John C. Doyle, Hiroaki Kitano, Adam P. Arkin, Benjamin J. Bornstein, Dennis Bray, Athel Cornish-Bowden, Autumn A. Cuellar, Sergey Dronov, Ernst Dieter Gilles, Martin Ginkel, Victoria Gor, Igor I. Goryanin, Warren J. Hedley, T. Charles Hodgman, Jan-Hendrik S. Hofmeyr, Peter J. Hunter, Nick S. Juty, Jay L. Kasberger, Andreas Kremling, Ursula Kummer, Nicolas Le Novère, Leslie M. Loew, Daniel Lucio, Pedro Mendes, Eric Minch, Eric D. Mjolsness, Yoichi Nakayama, Melanie R. Nelson, Poul F. Nielsen, Takeshi Sakurada, James C. Schaff, Bruce E. Shapiro, Thomas Simon Shimizu, Hugh D. Spence, Jörg Stelling, Koichi Takahashi, Masaru Tomita, John M. Wagner, Jian Wang, and the rest of the SBML Forum. The systems biology markup language (SBML): a medium for representation and exchange of biochemical network mod-

els. *Bioinformatics*, 19(4):524–531, March 2003. doi:10.1093/bioinformatics/btg015. URL http://bioinformatics.oxfordjournals.org/cgi/content/abstract/19/4/524.

Michael Hucka, Frank T. Bergmann, Andreas Dräger, Stefan Hoops, Sarah M. Keating, Nicolas Le Novère, Chris J. Myers, Brett G. Olivier, Sven Sahle, James C. Schaff, Lucian P. Smith, Dagmar Waltemath, and Darren J. Wilkinson. Systems Biology Markup Language (SBML) Level 2 Version 5: Structures and Facilities for Model Definitions. *Journal of Integrative Bioinformatics*, 12(2):271, September 2015. doi:10.2390/biecoll-jib-2015-271. URL http://journal.imbio.de/article.php?aid=271.

Michael Hucka, Frank T. Bergmann, Claudine Chaouiya, Andreas Dräger, Stefan Hoops, Sarah M. Keating, Matthias König, Nicolas Le Novère, Chris J. Myers, Brett G. Olivier, Sven Sahle, James C. Schaff, Rahuman Sheriff, Lucian P. Smith, Dagmar Waltemath, Darren J. Wilkinson, and Fengkai Zhang. Systems Biology Markup Language (SBML) Level 3 Version 2 Core release 2. *Journal of Integrative Bioinformatics*, 16(2):1, June 2019. doi:10.1515/jib-2019-0021. URL https://www.degruyter.com/view/j/jib.ahead-of-print/jib-2019-0021/jib-2019-0021.xml.

Nick Juty, Nicolas Le Novère, and Camille Laibe. Identifiers. org and MIRIAM Registry: community resources to provide persistent identification. *Nucleic acids research*, 40(D1):D580–D586, November 2012. URL http://nar.oxfordjournals.org/content/40/D1/D580.short.

Roland Keller, Alexander Dörr, Akito Tabira, Akira Funahashi, Michael J. Ziller, Richard Adams, Nicolas Rodriguez, Nicolas Le Novère, Noriko Hiroi, Hannes Planatscher, Andreas Zell, and Andreas Dräger. The systems biology simulation core algorithm. *BMC Syst Biol*, 2013. doi:10.1186/1752-0509-7-55. URL http://www.biomedcentral.com/1752-0509/7/55/abstract.

Zachary A. King, Andreas Dräger, Ali Ebrahim, Nikolaus Sonnenschein, Nathan E. Lewis, and Bernhard O. Palsson. Escher: A web application for building, sharing, and embedding data-rich visualizations of biological pathways. *PLoS Computational Biology*, 11(8):e1004321, August 2015a. doi:10.1371/journal.pcbi.1004321. URL http://dx.doi.org/10.1371%2Fjournal.pcbi.1004321.

Zachary A. King, Justin S. Lu, Andreas Dräger, Philip C. Miller, Stephen Federowicz, Joshua A. Lerman, Ali Ebrahim, Bernhard O. Palsson, and Nathan E. Lewis. BiGG Models: A platform for integrating, standardizing, and sharing genome-scale models. *Nucleic Acids Research*, October 2015b. doi:10.1093/nar/gkv1049. URL http://nar.oxfordjournals.org/content/44/D1/D515.

Marcel Kronfeld, Andreas Dräger, Moritz Aschoff, and Andreas Zell. On the Benefits of Multimodal Optimization for Metabolic Network Modeling. In Ivo Grosse, Steffen Neumann, Stefan Posch, Falk Schreiber, and Peter Stadler, editors, *German Conference on Bioinformatics*

*(GCB 2009)*, volume P-157 of *Lecture Notes in Informatics*, pages 191–200, Halle (Saale), Germany, September 2009. German Informatics society. ISBN 978-3-88579-251-2. URL http://www.gcb2009.de/program.php.

Marcel Kronfeld, Hannes Planatscher, and Andreas Zell. The EvA2 Optimization Framework. In Christian Blum and Roberto Battiti, editors, *Learning and Intelligent Optimization*, volume 6073 of *Lecture Notes in Computer Science, LNCS*, pages 247–250. Springer, Berlin, Heidelberg, Venice, Italy, lion 2010 edition, January 2010. ISBN 978-3-642-13799-0. doi:10.1007/978-3-642-13800-3_27. URL https://link.springer.com/chapter/10.1007/978-3-642-13800-3_27. Special Session on Software for Optimization (LION-SWOP).

Rahuman S. Malik-Sheriff, Mihai Glont, Tung V. N. Nguyen, Krishna Tiwari, Matthew G. Roberts, Ashley Xavier, Manh T. Vu, Jinghao Men, Matthieu Maire, Sarubini Kananathan, Emma L. Fairbanks, Johannes P. Meyer, Chinmay Arankalle, Thawfeek M. Varusai, Vincent Knight-Schrijver, Lu. Li, Corina Dueñas Roca, Gaurhari Dass, Sarah M. Keating, Young M. Park, Nicola Buso, Nicolas Rodriguez, Michael Hucka, and Henning Hermjakob. BioModels—15 years of sharing computational models in life science. *Nucleic Acids Research*, November 2019. ISSN 0305-1048. doi:10.1093/nar/gkz1055. URL https://doi.org/10.1093/nar/gkz1055. gkz1055.

Monica L. Mo, Bernhard ØPalsson, and Markus J. Herrgård. Connecting extracellular metabolomic measurements to intracellular flux states in yeast. *BMC Systems Biology*, 3(1):37, 2009. ISSN 1752-0509. URL https://doi.org/10.1186/1752-0509-3-37.

Charles J. Norsigian, Neha Pusarla, John Luke McConn, James T. Yurkovich, Andreas Dräger, Bernhard O. Palsson, and Zachary A. King. BiGG Models 2020: multi-strain genome-scale models and expansion across the phylogenetic tree. *Nucleic Acids Research*, 11 2019. ISSN 0305-1048. doi:10.1093/nar/gkz1054. URL https://doi.org/10.1093/nar/gkz1054. gkz1054.

Brett G Olivier and Frank T. Bergmann. The Systems Biology Markup Language (SBML) Level 3 Package: Flux Balance Constraints. *Journal of Integrative Bioinformatics*, 12(2):269, September 2015. doi:10.2390/biecoll-jib-2015-269.

William H. Press, Brian P. Flannery, Saul A. Teukolsky, and William T. Vetterling. *Numerical Recipes in Fortran: The Art of Scientific Computing*. Cambridge University Press, 2 edition, September 1992. ISBN 052143064X. URL https://smile.amazon.com/dp/052143064X.

Ingo Rechenberg. *Evolutionsstrategie: Optimierung technischer Systeme nach Prinzipien der biologischen Evolution*. Fromman-Holzboog, Stuttgart, 1973.

Nicolas Rodriguez, Alex Thomas, Leandro Watanabe, Ibrahim Y. Vazirabad, Victor Kofia, Harold F. Gómez, Florian Mittag, Jakob Matthes, Jan D. Rudolph, Finja Wrzodek, Eugen Netz, Alexander Diamantikos, Johannes Eichner, Roland Keller, Clemens Wrzodek, Sebastian

Fröhlich, Nathan E. Lewis, Chris J. Myers, Nicolas Le Novère, Bernhard Ø. Palsson, Michael Hucka, and Andreas Dräger. JSBML 1.0: providing a smorgasbord of options to encode systems biology models. *Bioinformatics*, June 2015. doi:10.1093/bioinformatics/btv341. URL `http://bioinformatics.oxfordjournals.org/content/31/20/3383`.

Adrien Rougny, Vasundra Touré, Stuart Moodie, Irina Balaur, Tobias Czauderna, Hanna Borlinghaus, Ugur Dogrusoz, Alexander Mazein, Andreas Dräger, Michael L. Blinov, Alice C. Villéger, Robin Haw, Emek Demir, Huaiyu Mi, Anatoly Sorokin, Falk Schreiber, and Augustin Luna. Systems Biology Graphical Notation: Process Description language Level 1 Version 2.0. *Journal of Integrative Bioinformatics*, 16(2), June 2019. ISSN 1613-4516. doi:10.1515/jib-2019-0022. URL `https://www.degruyter.com/view/j/jib.ahead-of-print/jib-2019-0022/jib-2019-0022.xml`.

Hans-Paul Schwefel. *Evolutionsstrategie und numerische Optimierung*. Dr.-Ing. Thesis, Technical University of Berlin, Department of Process Engineering, 1975.

Rainer Storn. On the Usage of Differential Evolution for Function Optimization. In *1996 Biennial Conference of the North American Fuzzy Information Processing Society*, pages 519–523, Berkeley, CA, USA, 1996. IEEE, New York, USA. doi:10.1109/NAFIPS.1996.534789. URL `http://ieeexplore.ieee.org/xpl/freeabs_all.jsp?arnumber=534789`.

# Index


**Symbols**

*Application Programming Interface* (API) iii, 2
*Audio Video Interleave* (AVI) . . . . . . . . 33, 48
*Character-Separated Value* (CSV) . . iii, 2, 6, 14–16, 18, 20, 29, 32, 35, 40
*Differential Evolution* (DE) . . . . . . . . . . 14, 23
*Evolution Strategies* (ES) . . . . . . . . . . . . . . . 14
*Flash Video* (FLV) . . . . . . . . . . . . . . . . . . . . . 48
*Genetic Algorithms* (GA) . . . . . . . . . . . . . . . 14
*Genome-scale Metabolic Model* (GEM) . . 24
*Graphical User Interface* (GUI) . iii, 1, 2, 12, 35, 39
*Java™ Archive* (JAR) . . . . . . . . . . . . . . 3, 4, 24
*Java™ Development Kit* (JDK) . . . . . . . . . . . 3
*JavaScript Object Notation* (JSON) . . . . . . 24
*Java™ Virtual Machine* (JVM) . . iii, 3, 4, 42
*GNU Lesser General Public License* (LGPL) 41
*Minimal Information Required In the Annotation of Models* (MIRIAM) . . . . 24
*QuickTime File Format* (MOV) . . . . . . 33, 48
*Moving Picture Experts Group* (MPG) 33, 48
*Not a Number* (NaN) . . . . . . . . . 14, 28, 35, 38
*Ordinary Differential Equation* (ODE) . 2, 8, 11, 12, 31, 36
*Portable Document Format* (PDF) . . . . . . . 12
*Portable Network Graphics* (PNG) . . . 32, 48
*Particle Swarm Optimization* (PSO) . . 14, 23
*Random-Access Memory* (RAM) . . . . . . . . . 4
*Relative Squared Error* (RSE) . . . . 16, 17, 39
*Systems Biology Simulation Core Library* (SBSCL) iii, 1, 41, 46, 47
*Scalable Vector Graphics* (SVG) . . . . . 32, 48
*Unicode Transformation Format* (UTF) . . 40
*Windows Media Video* (WMV) . . . . . . . . . . 48

**A**

AbstractDESSolver . . . . . . . . . . . . . . . . . . . . 36
Apple Numbers . . . . . . . . . . . . . . . . . . . . . . . . 14

**B**

BiGG Models Database . . . . . . . . . . . . . 24, 31
BioModels Database . . . . . . . . . . . . . . . . . iii, 5

**C**

CellDesigner . . . . . . . . . . . . . . . . . . . . . . . . . . 5

**E**

EvA2 . . . . . . . iii, 1, 14, 17–19, 21–23, 31, 41
Excel . . . . . . . . . . . . . . . . . . . . . . . . . . 14, 28, 30

**F**

Fitness . . . . . . . . . . . . . . . . . . . . . . 16, 17, 21, 22

**G**

Garuda . . . . . . . . . . . . . . . . . . . . . 2, 3, 40, 46, 47
Google Sheets . . . . . . . . . . . . . . . . . . . . . . . . . 14

**H**

hasOnlySubstanceUnits . . . . . . . . . . . . . . . . . 36

**I**

iMM904 . . . . . . . . . . . . . . . . . 24, 25, 29, 31, 34

**J**

Java™ . . . . . . . . . . . . . . 1, 3, 4, 42, 43, 47, 48
JSBML . . . . . . . . . . . . . . . . . . . iii, 1, 26, 41, 46

**K**

Ken Burns effect . . . . . . . . . . . . . . . . . . . . . 2, 32

**L**

Language pack
    English . . . . . . . . . . . . . . . . . . . . . . . . . . . . 4
    German . . . . . . . . . . . . . . . . . . . . . . . . . . . . 4
LibreOffice Calc . . . . . . . . . . . . . . . . . . . . . . . 14

**O**

Operating System . . . . . 3, 4, 6, 13, 15, 19, 43
    Linux . . . . . . . . . . . . . . . . . . . . . 3, 6, 12, 19
    macOS . . . . . . . . . 3, 6, 12, 19, 41–43, 47
    MS Windows . . . . . . . . . . . . . . 3, 6, 12, 19

**S**

SBGN . . . . . . . . . . . . . . . . . . . . . . . iii, 1, 2, 7, 8
SBML . . iii, 1, 2, 5–8, 12, 14, 15, 24–26, 28, 29, 31, 35–37, 46
SBML Test Suite . . . . . . . . . . . . . . . . . . . . . . . iii

**T**

Tribes . . . . . . . . . . . . . . . . . . . . . . . . . . . . . . . 14
